# Metronomic Administration of Topotecan Alone and in Combination with Docetaxel Inhibits Epithelial–mesenchymal Transition in Aggressive Variant Prostate Cancers

**DOI:** 10.1158/2767-9764.CRC-22-0427

**Published:** 2023-07-19

**Authors:** Taraswi Mitra Ghosh, Suman Mazumder, Joshua Davis, Jyoti Yadav, Ayuba Akinpelu, Ahmed Alnaim, Harish Kumar, Razan Waliagha, Allison E. Church Bird, Soroush Rais-Bahrami, R. Curtis Bird, Panagiotis Mistriotis, Amarjit Mishra, Clayton C. Yates, Amit K. Mitra, Robert D. Arnold

**Affiliations:** 1 Department of Drug Discovery and Development, Harrison College of Pharmacy, Auburn University, Auburn, Alabama.; 2 Division of Urology, Department of Surgery, Mass General Brigham and Women's Hospital and Harvard Medical School, Boston, Massachusetts.; 3 Center for Pharmacogenomics and Single-Cell Omics (AUPharmGx), Harrison College of Pharmacy, Auburn University, Auburn, Alabama.; 4 Department of Pathobiology, College of Veterinary Medicine, Auburn University, Auburn, Alabama.; 5 Department of Chemical Engineering, Samuel Ginn College of Engineering, Auburn University, Auburn, Alabama.; 6 Department of Biology and Canter for Cancer Research, Tuskegee University, Tuskegee, Alabama.; 7 Flow Cytometry and High-Speed Cell Sorting Laboratory, Auburn University, Auburn, Alabama.; 8 UAB O'Neal Comprehensive Cancer Center, University of Alabama at Birmingham Heersink School of Medicine, Birmingham, Alabama.; 9 Department of Urology, University of Alabama at Birmingham Heersink School of Medicine, Birmingham, Alabama.; 10 Department of Radiology, University of Alabama at Birmingham Heersink School of Medicine, Birmingham, Alabama.; 11 Department of Pathology, University of Alabama at Birmingham Heersink School of Medicine, Birmingham, Alabama.; 12 Department of Pathology, Johns Hopkins University School of Medicine, Baltimore, Maryland.; 13 Sidney Kimmel Comprehensive Cancer Center, Johns Hopkins University School of Medicine, Baltimore, Maryland.; 14 Department of Urology, Johns Hopkins University School of Medicine, Baltimore, Maryland.

## Abstract

Prostate cancer is the second leading cause of noncutaneous cancer-related deaths in American men. Androgen deprivation therapy (ADT), radical prostatectomy, and radiotherapy remain the primary treatment for patients with early-stage prostate cancer (castration-sensitive prostate cancer). Following ADT, many patients ultimately develop metastatic castration-resistant prostate cancer (mCRPC). Standard chemotherapy options for CRPC are docetaxel (DTX) and cabazitaxel, which increase median survival, although the development of resistance is common. Cancer stem-like cells possess mesenchymal phenotypes [epithelial-to-mesenchymal transition (EMT)] and play crucial roles in tumor initiation and progression of mCRPC. We have shown that low-dose continuous administration of topotecan (METRO-TOPO) inhibits prostate cancer growth by interfering with key cancer pathway genes. This study utilized bulk and single-cell or whole-transcriptome analysis [(RNA sequencing (RNA-seq) and single-cell RNA sequencing (scRNA-seq)], and we observed greater expression of several EMT markers, including *Vimentin*, *hyaluronan synthase-3*, *S100 calcium binding protein A6*, *TGFB1*, *CD44*, *CD55*, and *CD109* in European American and African American aggressive variant prostate cancer (AVPC) subtypes—mCRPC, neuroendocrine variant (NEPC), and taxane-resistant. The taxane-resistant gene *FSCN1* was also expressed highly in single-cell subclonal populations in mCRPC. Furthermore, metronomic-topotecan single agent and combinations with DTX downregulated these EMT markers as well as CD44^+^ and CD44^+^/CD133^+^ “stem-like” cell populations. A microfluidic chip-based cell invasion assay revealed that METRO-TOPO treatment as a single agent or in combination with DTX was potentially effective against invasive prostate cancer spread. Our RNA-seq and scRNA-seq analysis were supported by *in silico* and *in vitro* studies, suggesting METRO-TOPO combined with DTX may inhibit oncogenic progression by reducing cancer stemness in AVPC through the inhibition of EMT markers and multiple oncogenic factors/pathways.

**Significance:**

The utilization of metronomic-like dosing regimens of topotecan alone and in combination with DTX resulted in the suppression of makers associated with EMT and stem-like cell populations in AVPC models. The identification of molecular signatures and their potential to serve as novel biomarkers for monitoring treatment efficacy and disease progression response to treatment efficacy and disease progression were achieved using bulk RNA-seq and single-cell-omics methodologies.

## Introduction

Prostate cancer is the second leading cause of noncutaneous cancer-related deaths in American men (www.cancer.org). The androgen receptor (AR) signaling pathway plays a pivotal role in prostate development and homeostasis, as well as in the progression of prostate cancer (1). Androgen deprivation therapy (ADT), following radical prostatectomy or radiotherapy, remains the main treatment for more advanced cases of castration-sensitive prostate cancer (CSPC). In many patients, ADT effectively suppresses prostate cancer during the first 12–24 months (1–3). However, many patients ultimately develop resistance and show metastatic spread, that is*,* metastatic castration-resistant prostate cancer (mCRPC) disease (4). The 2020 estimates of transition from non-castrate to mCRPC are approximately 15%, with a mortality rate of 19.5% (4). Neuroendocrine differentiation following ADT to aggressive treatment-resistant neuroendocrine prostate cancer (NEPC) has been estimated at >25% (5). Standard treatment options for CRPC/NEPC include sipuleucel-T, abiraterone acetate plus prednisone (AA/P), or chemotherapy with docetaxel (DTX; refs. 6–8). Cabazitaxel, AA/P, enzalutamide, and radium-223 are approved treatments of CRPC, often following DTX (6, 7). These combination treatments can increase median overall survival (OS) by approximately 1 year (9). Resistance development, characterized by increased PSA levels, is almost universal, resulting in a progression-free survival rate of around 0% after 3 years, often accompanied by significant side effects (10–12). Furthermore, African American (AA) men are more likely to be diagnosed or progress more rapidly to aggressive forms of mCRPC compared with other ethnicities (seer.cancer.gov). Moreover, overall treatment options remain limited, and survival is poor.

Several groups, including ours, have demonstrated that the presence of cancer stem-like cells (CSC) in tumors displaying mesenchymal phenotypes (epithelial-to-mesenchymal transdifferentiation, EMT) and CD44^+^/CD133^+^ cells, with self-renewal and differentiation capacities, play crucial roles in tumor progression and development of mCRPC (13–16). Furthermore, tumor heterogeneity and host-tumor microenvironments with divergent genetic profiles and molecular signatures represent a challenge for current therapies (17). Therefore, it is necessary to develop new therapeutic approaches to overcome drug resistance in European American (EA) and AA patients with prostate cancer, specifically for mCRPC/NEPC, to improve efficacy and increased OS.

Low-dose continuous drug exposure using metronomic-like (METRO) chemotherapy involves frequent administration of chemotherapeutic agents at low or fractionated doses at close intervals over prolonged periods of time (18, 19). METRO is an emerging treatment option that has shown promise for various cancer types, including prostate cancer (20–23). In our previous study, we reported that metronomic topotecan treatment (METRO-TOPO) was 2.4- to 18-fold more potent (*P* < 0.05) compared with conventional topotecan (CONV-TOPO) treatment in prostate cancer cell lines (22). We then performed animal study with METRO-TOPO versus CONV-TOPO and demonstrated that by day 17, METRO-TOPO treatment resulted in significantly (*P* ≤ 0.05) smaller tumor volume (65.4% ± 11.2%) compared with control (136% ± 14%) and CONV-TOPO–treated animals (138% ± 10%) in an aggressive xenograft tumor model of human prostate cancer implanted in male NCr athymic mice (22). This overall antitumor activity of METRO-TOPO was maintained through the end of the study, animals receiving METRO-TOPO dosing regimens had significantly (*P* ≤ 0.05) smaller tumor volumes (54.8% ± 16.5%) than animals receiving CONV-TOPO dosing (144% ± 11%) or the control group (207% ± 26%; ref. 22). To further investigate the impact of low-dose therapy, the effect of METRO-TOPO, CONV-TOPO intravenous administration on tumor volume was compared with implantation of ALZET micro-osmotic pumps in nude mice after tumor xenografts reached 200–300 mm^3^ (22). ALZET pumps were primed to deliver 2.45 mg/kg/day to achieve plasma concentrations at the experimentally determined IC_50_, that is, 4–5 ng/mL and 0.10 mg/kg/day (4% of the IC_50_ concentration; ref. 22). After 21 days of treatment, animals receiving “metro-like” dosing at 2.45 and 0.1 mg TOPO/kg/day had significantly (*P* ≤ 0.05) smaller tumor volumes compared with ALZET control animals with no observable toxicities (22). Molecular analysis revealed METRO-TOPO treatment antitumor activity was associated with the inhibition of major cancer pathway genes, angiogenesis, increasing tumor hypoxia, or normalizing the tumor vasculature to improve blood flow and drug delivery (21, 22). Several other studies have reported that low-dose oral TOPO is potent for patients with cancer (24–26). However, the clinical benefit of METRO-TOPO and a comprehensive understanding of its mechanism of action are not fully known.

Therefore, we performed pretreatment versus posttreatment bulk and single-cell RNA sequencing (scRNA-seq) to identify differentially expressed genes (DEG) for prostate cancer subtypes of EA and AA origin and potential molecular pathways associated with METRO-TOPO activity in aggressive variant prostate cancer (AVPC; mCRPC, NEPC, and EMT) at the tumor and subclonal levels and to gain molecular insights into METRO-TOPO activity. Furthermore, our RNA-seq and scRNA-seq data showed that METRO-TOPO treatment was potentially effective against the development of prostate cancer subclones with a reduction of treatment-resistant stem-like genes. Using *in vitro* model systems of treatment-refractory and treatment-emergent AVPC, we demonstrated that the METRO-TOPO showed efficacy as a single agent, as shown previously. We showed synergistic activity in combination with conventional taxanes (CONV-DTX) and METRO-TOPO. A microfluidic chip-based confined cell invasion assay was performed. The assay recapitulates the dimensionality of pores and longitudinal channel-like tracks encountered by cancer cells during migration to investigate the effect of METRO-TOPO on cancer cell invasion and stemness. Flow cytometry and cell sorting with prostate cancer stemness-specific antibodies (CD44, CD133) were accomplished to identify differential percentages of stem-like cell populations (CD44^+^, CD133^+^, and CD++) in all prostate cancer subtypes, including EA and AA, to evaluate the impact of METRO-TOPO in eroding “stem-like” (CD44^+^/CD133^+^) cell subpopulations. The effect of METRO-TOPO treatment on EMT gene expression (GE) and protein expression was determined, and a comparative analysis with whole-genome transcriptomics data and reverse matching DEGs from patients with prostate cancer were used to examine the potential clinical significance of METRO-TOPO treatments.

Therefore, using an innovative approach that integrated advanced molecular techniques [next-generation mRNA sequencing (mRNA-seq) and single-cell-omics technologies] with microfluidics, flow cytometry, MoFlo XPD Flow high speed-based cell sorting, cytotoxicity profiling, immunoblotting as *in vitro* studies, and patient databases (The Cancer Genome Atlas/TCGA for *in silico* validation), we conclude that METRO-TOPO has the potential to improve the clinical outcome in AVPC chemotherapy by enhancing the therapeutic efficacy of standard-of-care drugs and abrogating the possibilities of development of drug resistance. Such an evidence-based approach promises to minimize the chances of trial failures and improve the probability of clinical success.

## Materials and Methods

Chemicals and Reagents, details are provided in the Supplementary Materials and Methods.

### Human Prostate Cancer Cell Lines

AR^Low^ mCRPC/NEPC (PC3, PC3M, DU145), AR^High^ EA mCSPC (LNCaP, 22Rv1, VCaP), AR^High^ EA mCRPC (C4-2B), AR^High^ AA mCSPC (MDA-Pca-2b), and normal prostate (RWPE1, RWPE2) cell lines were obtained from ATCC. AA-AR^High^ CSPC (RC77T, RC165T) and AA-AR^Low^ mCRPC (RC43T) were obtained from our collaborator's lab (Dr. Clayton Yates, Sidney Kimmel Comprehensive Cancer Center, Johns Hopkins University School of Medicine, Baltimore, MD; ref. 27). The taxane-resistant prostate cancer cell lines PC3-TXR and DUTXR were obtained from our collaborator's lab (Dr. Amit K. Mitra, Department of Drug Discovery and Development, Harrison College of Pharmacy, Auburn University, Aurburn, AL). The taxane-resistant cell lines PC3-TXR and DUTXR were generated using dose escalation with taxanes over time (28). Cell lines were authenticated at the source and tested randomly at regular intervals for provenance and cell lineage at the Center for Pharmacogenomics and Single-Cell Omics (AUPharmGx) using GenePrint 24 System. All cell lines were *Mycoplasma* negative and cultured with appropriate media (details provided in Supplementary Materials and Methods) at 37°C, 21% O_2_, and 5% CO_2_ in a humidified cell incubator (Thermo Fisher Scientific). Cells were subcultured when they reached approximately 80%–90% confluence. Cell lines were used until passage 30. After removing from the cryo-storage (passage 2–3), cells were cultured and passaged for 2–3 weeks before beginning studies.

### Treatment Schedules for mRNA-seq and scRNA-seq

Three-dimensional (3D) spheroids were harvested and transferred into a 96-well plate in 100 μL of recommended media. After 2 days of acclimation, 100 μL of media ± drug/well were added to each well containing a spheroid. On days 3 and 5, 100 μL of media/well were removed and replaced with 100 μL of fresh media with/without (±) drug/well. On off-media exchange days, 10 μL of media/well was removed and replaced with 10 μL of media ± drug. METRO-TOPO dosing was simulated by using 20x TOPO directly spiked into the wells at 10 μL in 190 μL of media. The CONV-TOPO was added as a bolus dose on day 0. METRO/extended exposure (EE)-TOPO treatment was given daily as a fractionated dose at 1/7th the CONV-TOPO. Total topotecan dosing was 100 nmol/L during each week of therapy. Control and treated spheroid were collected and stored in RNAlaterer (Qiagen) for RNA-seq. Fresh/live samples were used for scRNA-seq.

### Pretreatment and Posttreatment Tumor mRNA-seq

GE of all prostate cancer and normal prostate cell lines at baseline (no treatment) was assessed by RNA-seq. Furthermore, the effects of METRO-TOPO and CONV-TOPO exposure for 6 weeks on AR^Low^ mCRPC/NEPC PC-3 tumor model (3D spheroid) were assessed using RNA-seq. Pre- and post-drug exposure, as described above, tumor cells were harvested, and high-quality RNA was extracted using QIAshredder and RNeasy kit (Qiagen). RNA concentration and integrity were assessed using a Nanodrop-8000 spectrophotometer and Agilent 2100 Bioanalyzer. An RNA integrity number threshold >8 was applied, and RNA-seq libraries were constructed using Illumina TruSeq RNA Sample Preparation kit v2. Libraries were then size-selected to generate inserts of approximately 200 bp. RNA-seq was performed on Illumina's NovaSeq platform using a 150 bp paired-end protocol with a depth of >20 million reads per sample. Average quality scores were above Q30 for all libraries in both R1 and R2 (29). AA cell line RNA was isolated from cultured cells using TRIzol Reagent (Sigma Life Sciences) following the manufacturer's protocol (27). Library preparation, quality control, and sequencing of extracted RNA were performed by the Center for Pharmacogenomics and Single-Cell Omics (AUPharmGX).

### RNA-seq Data Analysis

RNA-seq data analysis was performed using a command line–based analysis pipeline (DEseq2 and edgeR) and Partek Flow software. Briefly, reads were preprocessed and mapped to the hg38 human genome build using the STAR Aligner tool. Next, mapped read counts were counts per million (CPM) normalized, and differential Gene Expression Profile (GEP) analysis was performed. Genes with mean fold change >|1| and *P* < 0.05 were considered the threshold for reporting significant DEGs. Heat maps were generated using unsupervised hierarchical clustering (HC) analysis based on top DEGs. Sequencing data on AA cell lines were compiled as FastQ files for downstream analysis (27).

### scRNA-seq

scRNA-seq analysis was assessed for prostate cancer cell lines at baseline and TOPO-METRO (EE vs. CONV for 6 weeks, as described above) in the PC-3 tumor model (3D spheroid). Automated single-cell capture and cDNA synthesis were performed using the 10X Genomics Chromium platform. scRNA-seq–based GE analysis was performed on an Illumina HiSeq 2500 next-generation sequencing (NGS) platform by paired-end sequencing technique at 2*125 bp and 100 cycles using v3 chemistry.

### scRNA-seq Data Analysis

scRNA-seq datasets were obtained as matrices in the Hierarchical Data Format (HDF5 or H5). CellRanger, Seurat, and Partek Flow software packages were used to preprocess the data analysis. Highly variable genes were selected for clustering analysis based on a graph-based cluster approach. The visualization of cell populations was performed by t-distributed stochastic neighbor embedding (t-SNE) and Uniform Manifold Approximation and Projection (UMAP) for dimension reduction analysis for biomarker-based identification of subclones representing CSC-EMT, taxane-resistant cells, potential METRO-TOPO targeted subclones, and METRO-TOPO treatment-induced erosion of these subclones.

### Ingenuity Pathway Analysis

Ingenuity Pathway Analysis (IPA; Qiagen) was performed using top DEGs to reveal molecular pathways/mechanisms, upstream regulator molecules, downstream effects, biological processes, and predicted causal networks governing prostate cancer subtypes and METRO-TOPO functions in AVPC (30).

### Patient Samples

In this study, the interactive web portals UALCAN and Gene Expression Profiling Interactive Analysis (GEPIA) were used to compare transcriptome data on target candidate pathway genes with tumor metastasis and patient survival from the prostate expression data matrix (31, 32). GE profiles for patients with prostate cancer included in TCGA database were used. Furthermore, GE data on deidentified patients with prostate cancer were extracted from the Genomic Data Commons server of TCGA database (Genomic Data Commons server/cancergenome.nih.gov). UALCAN (http://ualcan.path.uab.edu/index.html), a web-based tool for analyzing TCGA RNA-seq and clinical data, was used to evaluate the association of GE and patient survival in 31 types of cancers, including prostate cancer (30). Furthermore, GEPIA (http://gepia.cancer-pku.cn/), an interactive web-based tool for survival analysis based on GE, was also used. According to the characteristics of gene normalization, GEPIA allowed two different genes to be input at the same time for survival analysis (32).

### Flow Cytometry Detection of CD44^+^, CD133^+^, and CD44^+^/CD133^+^ Cells

Prostate cancer cells were labeled with a binding buffer containing stemness markers CD44, CD133, or both antibodies. Cells were collected and quantified as CD44^+^, CD133^+^, and double-positive cells using a Beckman Coulter Analytical Flow Cytometer—a CytoFLEX LX flow cytometer at 50,000 events/measurement. “Stem-like” cell populations were measured by assessing the shift in the mean fluorescence intensity by flow cytometry. Data were analyzed (gates were set), normalizing to unstained cells of each prostate cancer subtype. Ghost Dye Red 780 was used to detect live cells in each cell population. Furthermore, AR^Low^ cells were seeded in 6-well plates and exposed to CONV-TOPO, METRO-TOPO, and CONV-DTX+METRO-TOPO as combination treatments calculated on the basis of our earlier studies (22, 29). We have determined IC_50_ for MERO-TOPO, CONV-TOPO, and CONV-DTX as single agents for 48 and 72 hours for all prostate cancer cell lines (21, 22, 28). Plated cells were exposed to TOPO and DTX at the estimated 3-(4, 5-Dimethylthiazol-2-yl)-2,5-diphenyltetrazolium bromide (MTT) IC_50/2_ for each treatment protocol (CONV and METRO) for flow cytometry. For combination, prostate cancer cell lines were treated with estimated MTT IC_50_, IC_50/2_ for CONV-DTX and MTT IC_50_, IC_50/2_ for METRO-TOPO (Supplementary Table S5). After 72 hours, cells were labeled and quantified for CD44^+^, CD133^+^, and double-positive cells with the protocol above. All Flow Cytometry data were analyzed by using FlowJo software (https://www.flowjo.com/).

### *In Vitro* Cytotoxicity (MTT) Assay

*In vitro* cytotoxicity assays were performed using the MTT assay. Briefly, cells were plated in a 96-well culture plate at 2 × 10^3^ cells/well and incubated for 24 hours at 37°C with 5% CO_2_. We have determined IC_50_ for MERO-TOPO, CONV-TOPO, and CONV-DTX for 48 and 72 hours for all prostate cancer cell lines (21, 28). The specific IC_50_ and IC_50/2_ are provided in Supplementary Table S5. Cells were treated at the IC_50_ and IC_50/2_ estimated by MTT for each treatment protocol (CONV and METRO) for single agent (21, 22, 28). For combination studies, each prostate cancer cell line was treated at IC_50_ and IC_50/2_ estimated by MTT for CONV-DTX and METRO-TOPO. Following 72 hours incubation, MTT was added, and absorbance was measured at 550 nm using a Synergy Neo2 Microplate Reader. All experiments were performed in triplicate with five replicate wells for each concentration. Percent change in MTT staining (a surrogate for cell survival) relative to untreated controls was calculated at each drug concentration.

### Caspase 3/7 Activity Assay

Cell death by apoptosis was measured using a Caspase-Glo 3/7 kit. Briefly, cells were seeded and treated according to the MTT protocol (described above; Supplementary Table S5; refs. 22, 29). Following 72 hours incubation, Caspase-Glo 3/7 reagent was added, and luminescence was measured using a Synergy Neo2 Microplate Reader. The apoptotic level of each treatment group was normalized to the no drug treatment (baseline) caspase 3/7 for each cell line.

### Assessment of Cellular Morphology

Cellular morphology was assessed after cells were seeded at 2.5 × 10^4^ cells (mL/well) in 6-well plates and exposed to TOPO-CONV, TOPO-METRO, CONV-DTX as a single agent and in combination with CONV-DTX+METRO-TOPO according to MTT protocol (described above; Supplementary Table S5; refs. 22, 29). After 72 hours, cells were stained with crystal violet and washed with PBS. Three areas with approximately equal cell densities were identified in each well. Images were captured with an Agilent Cytation5 digital cell imaging system using a 4X objective and Texas Red filter with 559-34 Excitation, 630-69 Emission, and 585 DM. Images were analyzed using ImageJ software (https://imagej.nih.gov/ij/) in a double-blind manner.

### Aldefluor Activity (Aldehyde Dehydrogenase) Assay

Aldehyde dehydrogenase (ALDH1) activity was assessed using an Aldefluor assay kit. Briefly, 1 × 10^4^ prostate cancer cells were harvested and resuspended in Aldefluor assay buffer containing the ALDH substrate, BODIPY-amino acetaldehyde (BAAA). Negative control samples were treated with diethylamino-benzaldehyde (DEAB), an inhibitor of ALDH1 enzymatic activity. Cells were incubated at 37°C and suspended in Aldefluor assay buffer. The brightly fluorescent ALDH^+^ cells were detected by BD LSR II flow cytometry.

### Immunoblotting

Pretreatment and Posttreatment cells were lysed in RIPA Lysis and Extraction Buffer (Thermo Fisher Scientific). Briefly, cells were seeded and treated according to the MTT protocol (described above; Supplementary Table S5; refs. 21, 22, 28). Quantification of proteins was performed using a Bradford assay with a BSA protein standard kit (Bio-Rad). Equal amounts of protein were loaded onto 4%–15% Criterion TGX Stain-Free Precast Gels. Proteins were separated under reducing conditions and then transferred to a polyvinylidene difluoride membrane using a Bio-Rad Semi-dry Blotting Apparatus. Nonspecific binding was limited by incubating the membrane in blocking buffer [2.5% (w/v) casein, pH 7.6, 150 mmol/L NaCl, 10 mmol/L TRIS-HCl, and 0.02% sodium azide]. Membranes were then incubated with primary antibodies for the targeted gene/protein (1:1,000), followed by secondary antibodies (1:10,000). Immunoreactivity was detected using enhanced chemiluminescence Western Blotting substrate. Images were captured on a Gel Doc EZ Gel Documentation System using ImageLab software (Azure Biosystems). Densitometry analysis was performed using ImageJ software (https://imagej.nih.gov/ij/).

### Microfluidic Cell Migration Assay

The fabrication of the polydimethylsiloxane (PDMS)-based microchannel device using standard multilayer photolithography and replica molding has been reported earlier (33). Briefly, cells were seeded and treated according to the MTT protocol (described above; Supplementary Table S5; refs. 22, 23,34). Pretreated and posttreated 1–1.5 × 10^5^ cells were introduced into the cell seeding inlet line of the microfluidic channel via pressure-driven flow and were allowed to adhere for 10 minutes at 37°C, 5% CO_2_. Medium supplemented with 10% FBS was added into the chemoattractant inlet line to trigger cell entry into confining [(*W*) width × (*H*) height = 3 × 10 μm^2^] or partially confining (*W* × *H* = 10 × 10 μm^2^) microchannels. The devices were placed on an automated Nikon Ti2 Inverted Microscope equipped with a Tokai stage-top incubator. Cell entry into the channels was recorded via time-lapse microscopy. Images were recorded every 10 minutes for 8 hours with a 10x/0.45 NA Ph1 objective. The percentage of cell entry into microchannels was calculated by measuring the number of cells entering the channels divided by the total number of cells seeded adjacent to the microchannel entrances.

### Colony Formation Assay

DUTXR cells were seeded in a 6-well plate at 2.5 × 10^4^ cells (mL/well), incubated overnight_,_ and treated with CONV-TOPO, METRO-TOPO, CONV-DTX as a single agent, and CONV-DTX+METRO-TOPO in combination according to the MTT protocol (describe above; Supplementary Table S5; refs. 22, 29). The cells were harvested from a 24-well plate at 1,000 cells/well and incubated for 1–2 weeks. The colonies were fixed with 100% methanol and stained with crystal violet. Images were taken of cell colonies using an EVOS FL digital cell imaging system (Thermo Fisher Scientific). Images were recorded in brightfield and phase-contrast modes at 1X magnification and analyzed using ImageJ software https://imagej.nih.gov/ij/.

### FACS

Cells were stained with stemness markers (CD44), sorted using CD44^+^ versus CD44^−^ and plated for treatment. After overnight incubation, cells were treated with CONV-TOPO, METRO-TOPO, CONV-DTX, and the combination CONV-DTX+METRO-TOPO according to the MTT protocol (described above; Supplementary Table S5; refs. 22, 29)_._ Cell cytotoxicity, cellular morphology, and apoptosis were estimated using MTT, imaging, and caspase 3/7 assays.

### Statistical Analysis

All tests were two sided. Differences with *P* values <0.05 were considered significant. ANOVA was performed for continuous outcomes, and the Benjamini–Hochberg multiple testing methods were used as a *post hoc* test. The two-group *t* test was used to perform differential GE analysis between groups and detect the DEGs. Genes with mean fold changes>|1| and *P* < 0.05 were considered as the threshold for reporting significant differential GE. Finally, heat maps were generated using unsupervised HC analysis based on the top DEGs. Kaplan–Meier curves were generated using survival time and censored data to plot survival against high versus low expression of significantly associated genes. All statistical analyses were performed using R v4.1.0 and GraphPad Prism v9.0.

### Data Availability Statement

The datasets generated during or analyzed during the current study are available at the NCBI Gene Expression Omnibus (https://www.ncbi.nlm.nih.gov/geo/), accession number GSE233341 or from the corresponding author upon reasonable request.

## Results

### GEP Revealed Signatures for Each Prostate Cancer Subtype in EA versus AA Cell Lines

**FIGURE 1:**
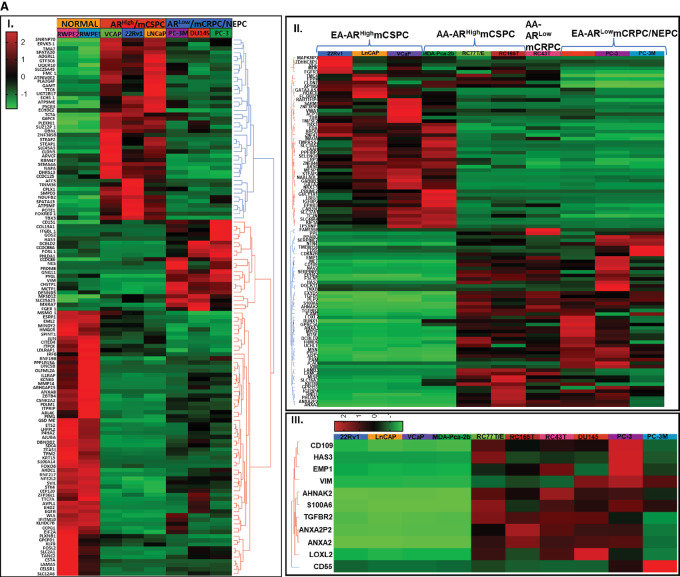

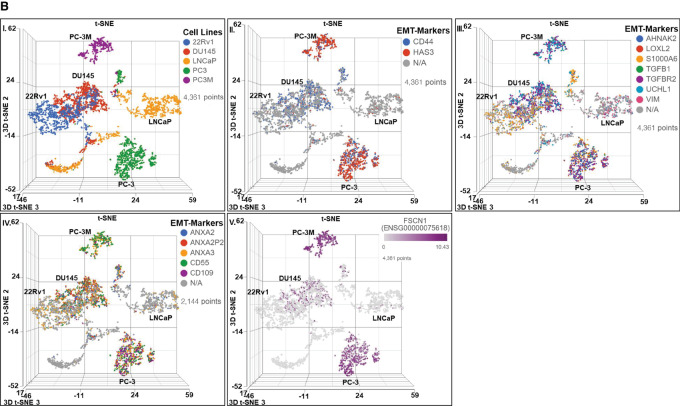

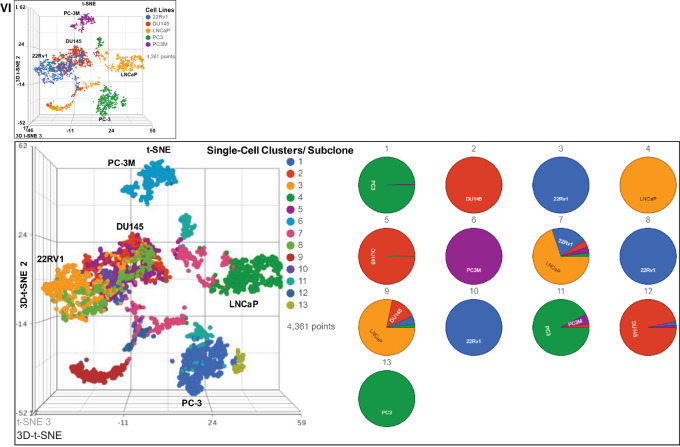

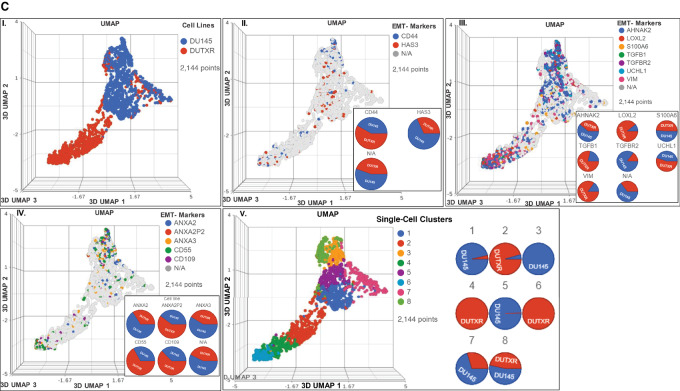
NGS and single-cell transcriptomics identified signatures of EMT markers in AR^High^/mCSPC, AR^Low^/mCRPC/NEPC, AR^Low^/mCSPC/NEPC^taxane resistant^ EA/Caucasian and AA prostate cancer subtypes. **A,** Heat map representing DEG signature from bulk RNA-seq. (**I**) DEGs among AR^High^/mCSPC (LNCaP, VCAP, 22RV1) versus AR^Low^/mCRPC/NEPC (PC-3, PC-3M, DU145) prostate cancer and normal prostate cell lines (RWPE1, RWPE2; *P* < 0.05). (**II**) DEGs among top 100 genes in AR^High^/mCSPC (EA-LNCaP, VCAP, 22RV1 vs. AA-MDA-Pca-2b, RC77T/E, RC165T) and AR^Low^/mCRPC (EA-DU145, PC-3, PC-3M vs. AA-RC43T; *P* < 0.05). (**III**) DEGs among top EMT markers in AR^High^/mCSPC (EA-LNCaP, VCAP, 22RV1 vs. AA-MDA-Pca-2b, RC77T/E, RC165T) and AR^Low^/mCRPC/NEPC (EA-DU145, PC-3, PC-3M vs. AA-RC43T) prostate cancer cell lines (*P* < 0.05). **B,** EMT markers in AR^High^/mCSPC versus AR^Low^/mCRPC (scRNA-seq): Single-cell RNA sequencing using the droplet sequencing method (10X Genomics) was performed on the prostate cancer cell lines. Each dot represents a single cell. Contaminated (doublet) cells were not included. t-SNE plots showing the comparison between the single-cell clusters. (**I**) Subclonal distribution in all cell lines AR^High^/mCSPC (LNCaP, 22RV1) and AR^Low^/mCRPC/NEPC (PC-3, PC-3M, DU145). (**II**) Subclonal distribution of top two EMT markers (*CD44* and *HAS3*) in all prostate cancer cell lines. (**III** and **IV**) Subclonal distribution of major EMT markers (*AHNAK2*, *LOXL2*, *S1000A6*, *TGFB1*, *TGFBR2*, *UCHL1*, *VIM*, *ANXA2*, *ANXA2P2*, *ANXA3*, *CD55*, *CD109*) in all prostate cancer cell lines. (**V**) Subclonal distribution of top taxane resistance marker *FSCN1* in all prostate cancer cell lines. Results exhibiting EMT and taxane resistance marker expression were higher in AR^Low^/mCRPC/NEPC compared with AR^High^/mCSPC. (**VI**) Subclonal populations for all prostate cancer subtypes. The relative representation of each subclone between the prostate cancer cell lines was also provided using comparative pie charts. Results showed the intratumor heterogeneity and several clusters which are unique to each prostate cancer cell line, in addition to the shared subclusters possibly representing gene signatures common to the biology of prostate cancer. **C,** EMT markers in AR^Low^/mCRPC/NEPC versus AR^Low^/mCSPC/NEPC^taxane-resistant^ prostate cancer: UMAP-distributed stochastic neighbor embedded plots showing the comparison between the single-cell clusters. (**I**) Subclonal distribution between AR^Low^/mCRPC/NEPC (DU145) and AR^Low^/mCSPC/NEPC^taxane-resistant^ (DUTXR). (**II**) Subclonal distribution of top two EMT markers *CD44* and *HAS3* in DU145 versus DUTXR. Pie charts represent percentages of subclonal distribution of EMT markers. *CD44* expression is higher in DUTXR and *HAS-3* expression higher in DU145. (**III**) Subclonal distribution of major EMT markers (*AHNAK2*, *LOXL2*, *S1000A6*, *TGFB1*, *TGFBR2*, *UCHL1*, *VIM*. (**IV**) *ANXA2*, *ANXA2P2*, *ANXA3*, *CD55*, *CD109*) in DU145 versus DUTXR. Pie chart represented majority of EMT markers (*LOXL2*, *TGFB1*, *UCHL1*, *VIM*, *ANXA2P2*, *CD55*, *CD109*) expressed higher in DUTXR cell line. Results exhibiting EMT marker expression were high (EMT markets nonexpressing cells were low) in the AR^Low^/mCSPC/NEPC^taxane-resistant^ prostate cancer cell line (DUTXR). **V,** Top genes representing single-cell clusters that were shared and which were unique between DU145 and DUTXR. Common and unique DEGs between RNA-seq and scRNA-seq were also identified for all prostate cancer subtypes. Common genes were EMT markers, for example, *VIM*, *AHNAK2*, *ANXA1*, *ANXA2*, *CD109*, *CD44*, *CD59*, *GPRC5A*, *HAS*, *S100A6*, *TGFBR2* (Supplementary Figs. S4 and S5; Supplementary Table S7).

Baseline GEP identified DEGs for each prostate cancer subtype AR^High^/mCSPC (LNCaP, VCAP, 22RV1), AR^Low^/mCRPC/NEPC (PC-3, PC-3M, DU145) and normal prostate cell lines (RWPE1 and RWPE2; Fig. 1AI; Supplementary Fig. S1A). Among the top 100 (FDR < 0.05, *P* < 0.05) DEGs for AR^Low^/mCRPC/NEPC cell lines were the majority of EMT markers (upregulated) such as Vimentin (*VIM*), hyaluronan synthase-3 (*HAS3*), Laminin subunit beta-3 (*LAMB3*), S100 calcium binding protein A6 (*S100A6*), Serpin-E2 (*SERPINE2*), insulin-like growth factor binding protein-4 (*IGFBP4*), Annexin A2 (*ANXA2*), Annexin A2 pseudogene-2 (*ANXA2P2*), G protein-coupled receptor class C-5A (*GPRC5A*), and *CD55*, whereas downregulated genes were included endothelial transcription factor *GATA2*, nerve growth factor inducible *VGF/VGF*, insulin-like growth factor binding protein-2 (*IGFBP2*), solute carrier family 44-A4 (*SLC44A4*), guanylate cyclase-1 alpha-1 (*GUCY1A1*), NK3 homeobox-1 (*NKX3-1*), kinesin-1A (*KIF1A*), prune homolog-2 with BCH domain (*PRUNE2*), transmembrane serine protease-2 (*TMPRSS2*), and acid phosphatase-3 (*ACP3*; Table 1; Supplementary Fig. S1B). In addition, GEPs between AR^High^/mCSPC-EA (LNCaP, VCAP, 22RV1) and AR^High^/mCSPC-AA (MDA-Pca-2b, RC77T, RC165T, and RC43T) showed discrete DEGs (Fig. 1AII). Top EMT markers *VIM*, *HAS3*, *CD55* were downregulated in all AA cell lines. Furthermore, *ANXA2*, *ANXA2P2F*, *S100A6* were downregulated in MDA-Pca-2b and upregulated in RC77T, RC165T and RC43T. Additional EMT markers *AHNAK2*, *TGFB1*, *TGFBR2*, *UCHL1*, *ANXA3*, and *LOXL2* were also downregulated in MDA-PCa-2b and upregulated in other AA cell lines (RC77T, RC165T, and RC43T; Fig. 1AIII). Fascin actin-bundling protein-1/*FSCN1*, a taxane-resistant gene, was identified as a top DEG among all prostate cancer subtypes (Table 1).

**TABLE 1:** Top DEGs for mCSPC/AR^High^ (LNCaP, VCaP, 22RV1) and mCRPC/NEPC/AR^Low^ (PC-3, PC3M, and DU145) prostate cancer cell lines

GENE	*P* value (mCRPC/NEPC/AR^Low^) vs. mCSPC/AR^High^)	FDR (mCRPC/NEPC/AR^Low^ vs. mCSPC/AR^High^)	Fold change (mCRPC/NEPC/AR^Low^ vs. mCSPC/AR^High^)
*VIM*	2.99328E-05	1.42E-02	5.42
*HAS3*	5.4116E-05	1.80E-02	4.84
*LAMB3*	0.003776629	9.97E-02	4.74
*FSCN1*	2.30966E-06	6.22E-03	4.71
*S100A6*	0.000291078	3.54E-02	4.70
*SERPINE2*	0.001903913	7.08E-02	4.67
*IGFBP4*	0.003936626	1.01E-01	4.65
*ANXA2P2*	4.59682E-05	1.62E-02	4.58
*GPRC5A*	0.000182691	3.08E-02	4.57
*ANXA2*	2.26583E-05	1.35E-02	4.51
*CD55*	0.005292675	1.18E-01	4.46
*MYOF*	0.00010936	2.41E-02	4.29
*AHNAK2*	1.72195E-07	1.52E-03	4.26
*NTN4*	8.16229E-05	2.15E-02	4.20
*EMP1*	0.001951103	7.12E-02	4.07
*PYGL*	4.09496E-07	2.41E-03	4.06
*MET*	0.000155609	2.95E-02	4.05
*NT5E*	0.000998362	5.33E-02	4.05
*ANXA3*	0.000591332	4.57E-02	4.02
*MSN*	0.000401162	3.85E-02	3.99
*CDKN2B*	0.000464697	4.14E-02	3.99
*SERPINB5*	0.002231754	7.74E-02	3.98
*NAV2*	0.000797065	5.09E-02	3.95
*KRT80*	0.025760473	2.26E-01	3.87
*LOXL2*	0.001741083	6.85E-02	3.86
*LAMC2*	0.000306543	3.57E-02	3.78
*UCHL1*	0.000216813	3.24E-02	3.78
*JCAD*	0.000363004	3.75E-02	3.74
*PPARG*	0.022612598	2.14E-01	3.74
*SLC16A3*	1.65115E-05	1.21E-02	3.74
*EPHA2*	0.000139595	2.83E-02	3.73
*GPR153*	0.01229283	1.70E-01	3.70
*ICAM1*	0.010641141	1.59E-01	3.69
*MISP*	0.000710029	4.89E-02	3.67
*FRMD6*	0.000794029	5.09E-02	3.65
*PHLDA1*	0.000184001	3.08E-02	3.64
*TNXB*	0.024638923	2.22E-01	3.56
*DCBLD2*	2.26926E-05	1.35E-02	3.56
*FXYD5*	0.005260462	1.18E-01	3.56
*PLP2*	2.69796E-05	1.42E-02	3.55
*RND3*	0.001883901	7.05E-02	3.54
*RUNX1*	0.00319113	9.19E-02	3.50
*ARSJ*	0.001167731	5.68E-02	3.50
*DOCK11*	0.003935236	1.01E-01	3.48
*PAM*	2.88835E-06	6.22E-03	3.44
*CD109*	0.00210488	7.46E-02	3.43
*TGFB1*	0.015320768	1.84E-01	3.41
*PPL*	0.000901009	5.20E-02	3.38
*TGFBR2*	0.000875356	5.17E-02	3.31
*TMEM158*	0.005920273	1.23E-01	3.31
*VWA1*	0.044596554	2.83E-01	−2.69
*CLDN7*	0.002505984	8.24E-02	−2.70
*GATA2-AS1*	0.00939702	1.50E-01	−2.71
*FAAH*	0.00416822	1.04E-01	−2.71
*EDA*	0.005827322	1.23E-01	−2.71
*TUB*	0.010572981	1.59E-01	−2.72
*LRP3*	0.026139148	2.27E-01	−2.72
*FLJ20021*	0.002050508	7.31E-02	−2.76
*GABRB3*	0.023844573	2.19E-01	−2.77
*EPHB3*	0.003262993	9.26E-02	−2.79
*PPFIBP2*	0.011893063	1.67E-01	−2.80
*EPPK1*	0.001705898	6.75E-02	−2.80
*RAB11FIP4*	0.002994659	8.93E-02	−2.80
*ZNF43*	0.008793452	1.46E-01	−2.83
*ZNF844*	0.036959459	2.63E-01	−2.87
*MAPK8IP2*	0.002778951	8.61E-02	−2.89
*TMC4*	0.000132727	2.76E-02	−3.00
*RHOU*	7.57441E-05	2.15E-02	−3.03
*MDK*	0.030703993	2.44E-01	−3.05
*SLC37A1*	0.010283114	1.57E-01	−3.07
*CAB39L*	0.000989636	5.33E-02	−3.10
*MESP1*	0.005371205	1.18E-01	−3.13
*NAALADL2*	0.008906081	1.46E-01	−3.15
*SELENOP*	0.006901063	1.31E-01	−3.17
*ZDHHC8P1*	0.015350676	1.84E-01	−3.18
*TM7SF2*	0.000409827	3.85E-02	−3.23
*TC2N*	0.000987453	5.33E-02	−3.26
*FAM155B*	0.008037262	1.40E-01	−3.27
*HOXC6*	0.014848508	1.82E-01	−3.29
*TP53INP1*	0.004466901	1.08E-01	−3.29
*LRIG1*	0.012496784	1.71E-01	−3.30
*MAOA*	0.001758383	6.88E-02	−3.31
*ARSD*	0.029445995	2.39E-01	−3.32
*SARM1*	0.000142795	2.86E-02	−3.37
*SLC30A4*	0.008939266	1.46E-01	−3.40
*FGFR3*	0.001088995	5.51E-02	−3.49
*AP1M2*	0.008546189	1.44E-01	−3.55
*STEAP2*	1.38121E-05	1.21E-02	−3.60
*ZNF385B*	0.00540026	1.18E-01	−3.64
*TBX3*	1.14871E-05	1.21E-02	−3.68
*GATA2*	0.001235243	5.75E-02	−3.78
*VGF*	0.029071043	2.38E-01	−3.82
*IGFBP2*	0.010644291	1.59E-01	−3.83
*SLC44A4*	0.019128756	1.99E-01	−4.14
*GUCY1A1*	0.028745507	2.36E-01	−4.32
*NKX3-1*	0.00032971	3.62E-02	−4.34
*KIF1A*	0.007879273	1.39E-01	−4.35
*PRUNE2*	2.97551E-05	1.42E-02	−4.62
*TMPRSS2*	0.004236818	1.04E-01	−4.71
*ACP3*	0.036440605	2.61E-01	−5.80

NOTE: Gene expression was assessed by next-generation RNA sequencing. Fold change cut-off value was > 2. EMT markers *HAS3*, *VIM*, *AHNAK2*, *ANXA2*, *ANXA2P2*, *ANXA3*, *CD109*, *CD55*, *EMP1*, *FSCN1*, *S100A6*, were the top differentially expressed gene in mCSPC versus mCRPC.

IPA based on the DEGs revealed activation of cell mobility, morbidity, mortality, migration, invasion, survival, viability, development of vasculature-vasculogenesis, and angiogenesis were the key pathways (z score 8.175 to −7.005) associated with the development of more aggressive forms (AR^Low^/mCRPC/NEPC) of prostate cancer subtypes (Supplementary Fig. S2A). Furthermore, oxidative phosphorylation, p70S6K, unfolded response, TREM1, NFκB, ERK5, IL8, BAG2, Integrin, and VEGF signaling were identified (z score 5.574 to −4.811) as top canonical pathways for AR^Low^/mCRPC/NEPC prostate cancer (Supplementary Fig. S2B). IPA also predicted HIF1α (*P* < 9.43E-06) and EMT (*P* < 5.63E-06) as key pathways associated with prostate cancer development in AA (Supplementary Fig. S2C).

### GEP Identified Unique DEGs for Taxane-resistant AR^Low^/mCRPC/NEPC Lines (DUTXR and PC3-TXR)

RNA-seq identified DEGs between AR^Low^/mCRPC/NEPC (PC-3, DU145) and its taxane-resistant version AR^Low^/mCRPC/NEPC^taxane-resistant^ (PC-3TXR and DUTXR). GEP data from baseline DUTXR and PC3-TXR indicated 3,289 and 2,215 genes were uniquely expressed in PC-3TXR and DUTXR, respectively. Furthermore, GEP also identified 2,968 commonly expressed genes in both sensitive and TXR cell lines (Supplementary Fig. S3; Supplementary Table S6A and S6B). The top common genes *C1orf116*, *ICAM1*, *KRT7*, *SLC43A3*, *TGM2*, *TUBB2B* between DUTXR versus DU145 and PC-3TXR versus PC-3 were determined using RNA-seq analysis.

### scRNA-seq Identified EMT Markers in AR^Low^ and Acquired Taxane-resistant Prostate Cancer Subtypes

scRNA-seq analysis identified higher expression of CSC markers, EMT markers (*HAS3* and its receptor *CD44*), and “stem-like” subclonal cell populations in AR^Low^/mCRPC/NEPC (PC3, PC3M, and DU145) compared with AR^High^/mCSPC (22RV1, LnCaP) prostate cancer cell lines (Fig. 1BII). A higher expression of additional EMT markers (*AHNAK2*, *LOXL2*, *S100A6*, *TGFB*, *TGFBR2*, *UCHL1*, *VIM*, *ANXA2*, *ANXA2P2*, *ANXA3*, *CD55*, *CD109*) in AR^Low^/mCRPC/NEPC (PC3, PC3M and DU145) compared with AR^High^/mCSPC (22RV1, LnCaP) prostate cancer cell lines was also noted (Fig. 1BIII and IV). There was expression variation of each EMT marker among all AR^Low^/mCRPC/NEPC prostate cancer subtypes, for example, *CD44* expression was greater in PC3 and PC3M compared with DU145 (Fig. 1BII–IV). *CD55* expression was also higher in PC-3, whereas *CD109* was greater in PC-3M (Fig. 1BII–IV). A comparison of other major EMT markers between PC3 and PC-3M revealed *EZH2*, *Snail/SNAI1*, *Slug/SNAI2*, and *TWIST1*, were expressed highly in PC-3M compared with PC-3, yet both are AR^Low^/mCRPC/NEPC prostate cancer subtypes (Fig. 5A). Minute levels of EMT markers were also expressed in AR^High^/mCSPC cell lines (22RV1 and LNCaP; Fig. 1BII–IV). Notably, *FSCN1* associated with taxane-resistant expressed highly in single-cell subclonal populations in AR^Low^/mCRPC/NEPC prostate cancer (PC3, PC3M, and DU145; Fig. 1BV). In addition, the intratumor heterogeneity within various cell lines is revealed by single-cell clusters (representing subclonal populations) determined by t-SNE/UMAP analysis based on the expression of top biomarkers for each subcluster. The relative representation of each subclone between the prostate cancer cell lines was also provided using comparative pie charts. Our results showed several clusters which are unique to each prostate cancer cell line, in addition to the shared subclusters possibly representing gene signatures common to the biology of prostate cancer (Fig. IBVI).

Next, we compared the EMT markers between taxane-sensitive (DU145) and the clonally derived acquired taxane-resistant mCRPC cell line DUTXR. Some common overlap in GE was observed between the DUTXR and DU145 subclones, as expected (Fig.1CI). However, the subclonal analysis showed a greater percentage of cells not expressing EMT markers in taxane-sensitive DU145 cells compared with the taxane-resistant DUTXR cell line (Fig. 1CII–IV). This finding demonstrated that a greater percentage of DUTXR cells exhibited top EMT markers compared with DU145. The expression of *VIM*, *LOXL2*, *TGFB*, *TGFBR2*, *UCHL1*, *ANXA2P2*, *CD55*, and *CD109* was greater in taxane-resistant DUTXR subclonal populations compared with taxane-sensitive DU145 (Fig. 1CII–IV). While the expression of other prostate cancer EMT markers *HAS-3*, *AHNAK2*, *TGFBR2*, *ANXA2*, and *ANXA3* was higher in DU145 compared with DUTXR (Fig. 1CII–IV). Overall, the expression of EMT markers in subclonal cell populations was greatest in DUTXR.

Top genes representing single-cell clusters that were shared and which were unique between DU145 and DUTXR were further investigated using IPA pathway analysis and upstream prediction. The following clusters were unique to DU145: Cluster 1, Cluster 3, Cluster 5, and Cluster 7. Cluster 8 was shared between both the lines (Fig. 1CV). On the other hand, the following clusters were enriched in DUTXR: Cluster 2 (IPA predicted activation of PPARA), Cluster 4 (ERK/MAPK signaling, MSP-RON Signaling, upregulation of FOXM1, ESR1, CEBPB, XBP1, and NPM1), Cluster 6 (ATM signaling and MYC; Fig. 1CV; Supplementary Fig. S3BI and S3BII).

### RNA-seq versus scRNA-seq Analysis Identified Common and Unique DEGs Among all Prostate Cancer Subtypes

The analysis of RNA-seq and scRNA-seq data revealed 70 genes common to all prostate cancer subtypes, while scRNA-seq and RNA-seq analysis identified 582 and 73 unique DEGs, respectively (Supplementary Fig. S4; Supplementary Table S7). Among the common genes were EMT markers, for example, *VIM*, *AHNAK2*, *ANXA1*, *ANXA2*, *CD109*, *CD44*, *CD59*, *GPRC5A*, *HAS*, *S100A6*, *TGFBR2*. Moreover, the taxane-resistant gene *FSCN1* was also found to be a common gene in both cohorts of prostate cancer cell line models (Supplementary Table S7).

The IPA based on the common DEGs between RNA-seq and scRNA-seq predicted cell invasion, movement, neoplasia, migration, transformation, metastatic, and growth as key pathway-associated prostate cancer aggressiveness (Supplementary Fig. S5A). Furthermore, causal network pathway for common DEGs include *LY2109761* (TGFβ receptor inhibitor), *IGF2R* (receptor for both insulin-like growth factor 2), *IL11RA* (cytokine), *SEMG2* (semenogelin proteins), *CCR2* (chemokine receptor type 2), *TRAF3IP3* (mediates cell growth by modulating the c-Jun N-terminal kinase signal transduction pathway), *GGTI-2154* (inhibitor of geranylgeranyltransferase I), *L778123* (inhibitor of FPTase and GGPTase-I), *TGFB1*, and *CXCL5* (Supplementary Fig. S5B). Upstream regulators for common DEGs were also identified, *THBF1*, *RAF1*, *AGT*, *ER receptor*, *OSM*, *SLC15A4*, *JUN*, and *IL1B* (Supplementary Fig. S5C). The S100 family, hepatic fibrosis, Rho family, RHOGDI, and integrin singling pathway signaling were identified as top canonical pathways for prostate cancer (Supplementary Fig. S5D).

### *In Silico* Analysis Using Prostate Cancer Patient Transcriptomes Validated the Clinical Relevance of Top EMT Markers

**FIGURE 2:**
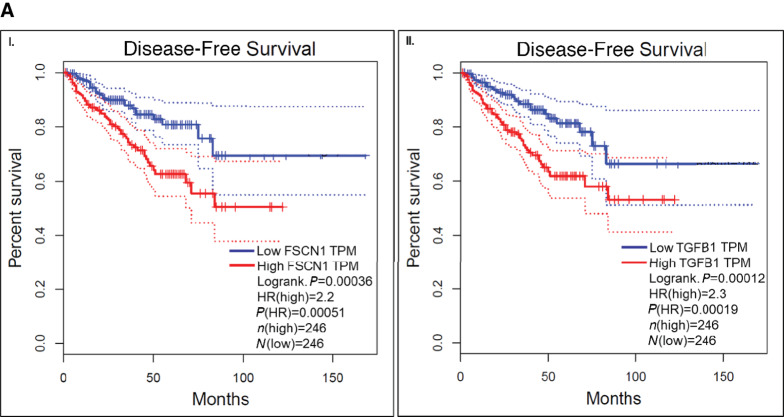

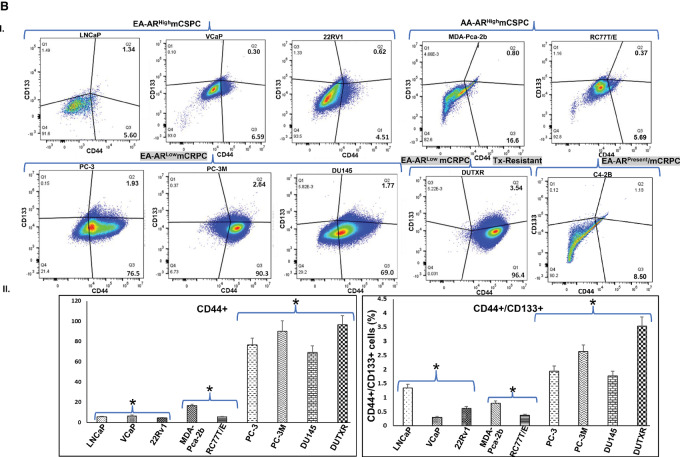

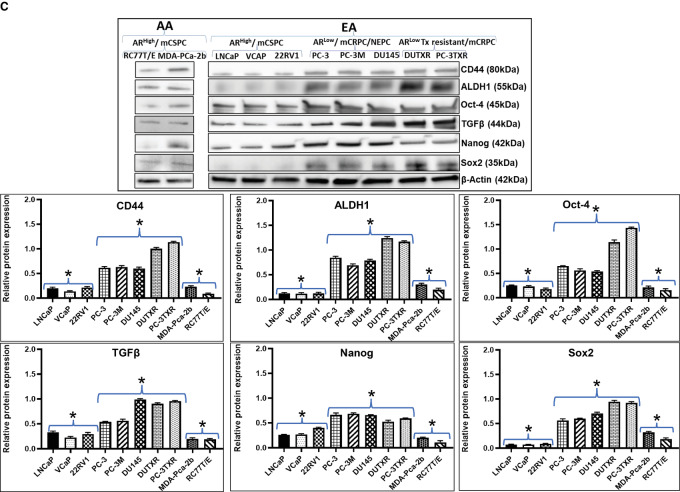
*In silico and in vitro* validation of top EMT marker's association with disease progression, patient survival, and disease aggressiveness. **A,***In silico* correlation of EMT signatures using TCGA database: Top EMT marker *TGFB1* and taxane resistance marker *FSCN1* were associated with DFS of patients with prostate cancer. (**I**) High expression of *FSCN1* was correlated with lower survival compared with the low expression group of patients (*P* < 0.0001). (**II**) Higher expression of *TGFB1* showed less survival compared with the low expression group of patients (*P* < 0.0001). **B,** Assessment of “stem-like” cells (CD44^high^/CD133^high^) population in prostate cancer subtypes by Flowcytometry: Assessing of “stem-like” cell population among all prostate cancer subtypes. (**I**) Baseline (no drug treatment) CD44^+^ cells were higher in EA-AR^Low^/mCRPC (PC-3-76.5%, PC-3M-90.0%, DU145-69.0%) compared with EA-AR^High^/mCSPC (LNCaP-5.60%, VCaP-6.69%, 22RV1-4.51%). Taxane-resistant AR^Low^/mCRPC (DUTXR) cells showed the highest percent of “stem-like” cells (CD44^+^ cells 96.4%). Furthermore, AA-AR^High^ /mCSPC (MDA-PCa-2b) showed higher percent of CD44^+^ (16.6%) cells compared with EA-AR^High^/mCSPC (LNCaP, VCaP, 22RV1). Whereas in contrast, another AA-AR^High^/mCSPC (RC77T/E) showed low percent CD44^+^ cells population (5.69%). EA-AR^Present^/mCRPC (C4-2B) showed CD44^+^ cell population (8.50%). (**II**) and (**III**) Bar graphs represented a comparison among CD44^+^ and CD44^+^/CD133^+^ cells in all prostate cancer subtypes. (*, *P* ≤ 0.05). Results represented higher stemlike cell population in AR^Low^/mCRPC and taxane-resistant mCRPC. **C,** Immunoblotting: Higher expression of EMT proteins (CD44, ALDH1, Oct-4, TGFβ, Nanog, and Sox2) was observed in AR^Low^/mCRPC/NEPC (PC-3, PC-3M, DU145) prostate cancer cell lines compared with AR^High^/mCSPC (LNCaP, VCaP, 22RV1) cell lines. Furthermore, higher expression of EMT proteins (CD44, ALDH1, Oct-4, TGFβ, Nanog, and Sox2) was observed in AA-AR^High^/mCSPC (MDA-PCa-2b, RC77T/E) compared with EA-AR^High^/mCSPC (LNCaP, VCaP, 22RV1) prostate cancer cell lines. Highest amount of EMT protein was expressed in taxane-resistant (DUTXR and PC-3TXR) prostate cancer cell lines. Immunoblotting results corroborated with DEGs. Densitometry plots represented a comparison of protein expression among all prostate cancer subtypes. Actin-β was used as a control housekeeping gene to normalize the protein expression of other genes for immunoblotting experiments (*, *P* ≤ 0.05).

An *in silico* analysis using TCGA dataset showed that the top gene, *FSCN1*, was significantly associated with disease-free survival (DFS), Kaplan–Meier curves (Fig. 2AI), with HR 2.2 (*P* = 0.00051). Low expression of *FSCN1* was associated with longer DFS. In contrast, high expression of *FSCN1* was associated with poor DFS. Another top EMT marker, *TGFB1*, showed similar trends. Low expression was associated with longer DFS, whereas high expression was associated with poor DFS (*P* = 0.00019, HR = 2.3; Fig. 2AII).

### Flow Cytometry Analysis Identified “Stem-like” (CD44^+^, CD133^+^, and CD44^+^/CD133^+^) Cells in All (EA and AA) Prostate Cancer Subtypes

CD44 is a well-known marker for “stem-like” cells in prostate cancer (35). The percentage of CD44^+^ cells was greater in AR^Low^/mCRPC/NEPC compared with AR^High^/mCSPC. The percentage of CD44^+^ cells was 76.5% in PC-3, 90.3% in PC-3M, and 69.0% in DU145, whereas they were 5.60% in LNCaP, 6.59% in VCaP, 4.51% in 22RV1, and 8.50% in C4-2B cell lines (Fig. 2B; Table 2). Interestingly, taxane-resistant DUTXR showed the greatest percentage of “stem-like” cells, 96.4% CD44^+^. Furthermore, CD133 was identified as another marker (nonabundant) for “stem-like” cells in prostate cancer (35). Therefore, we measured CD133 and CD44/133 in all prostate cancer subtypes; the results showed a similar trend (Fig. 2B; Table 2). In the AA prostate cancer subtype (MDA-PCa-2b and RC77T/E), “stem-like” CD44^+^ cell proportions were 16.6% and 5.69%, respectively (Fig. 2B; Table 2).

**TABLE 2:** Flow cytometry: Prostate cancer cell lines stained with stemness markers (CD44, CD133, and CD44/133) to determine the percent of CD44^+^, CD133^+^, and double-positive cells in prostate cancer subtypes

Cell lines	LNCaP	VCaP	22RV1	C4-2B	PC-3	PC-3M	DU145	DUTXR	MDA-Pca-2b	RC77T/E
Ethnicity	Caucasian	Caucasian	Caucasian	Caucasian	Caucasian	Caucasian	Caucasian	Caucasian	African American	African American
Characteristics	AR^High^ mCSPC	AR^High^ mCSPC	AR^High^ mCSPC	AR^High^ mCRPC	AR^Low^ mCRPC/ NEPC	AR^Low^ mCRPC/ NEPC	AR^Low^ mCRPC/ NEPC	AR^Low^ mCRPC/ NEPC^Taxane Resistant^	AR^High^ mCSPC	AR^High^ mCSPC
CD44 (No treatment)	5.60%	6.59%	4.51%	8.50%	76.50%	90.30%	69.00%	96.40%	16.60%	5.69%
CD44 (CONV-TOPO)	NA	NA	NA	NA	30.50%	42.20%	12.30%	81.00%	NA	NA
CD44 (METRO-TOPO)	NA	NA	NA	NA	19.50%	17.50%	8.84%	71.50%	NA	NA
CD44 (CONV-DTX+ METRO-TOPO)	NA	NA	NA	NA	5.97%	8.35%	3.39%	63.40%	NA	NA
CD44^+^CD133 (No treatment)	1.34%	0.30%	0.62%	1.18%	1.93%	2.64%	1.77%	3.54%	0.80%	0.37
CD44^+^CD133 (CONV-TOPO)	NA	NA	NA	NA	1.55%	0.75%	0.56%	1.19%	NA	NA
CD44^+^CD133 (METRO-TOPO)	NA	NA	NA	NA	0.26%	0.25%	0.51%	2.11%	NA	NA
CD44^+^CD133 (CONV-DTX+ METRO-TOPO)	NA	NA	NA	NA	0.50%	0.14%	0.40%	1.15%	NA	NA

NOTE: Baseline (no drug treatment) CD44^+^ is higher in AR-mCRPC/NEPC compared with AR^High^ mCSPC. AR^Low^ taxane-resistant DUTXR showed the highest percent of stemness. Treatments with CONV-TOPO, METRO-TOPO, and CONV-DTX+METRO-TOPO reduced stemness compared to CONV-TOPO and CONV-DTX treatments, whereas combination treatment with CONV-DTX+METRO-TOPO reduced the highest degree of stemness in AR^Low^/mCRPC/NEPC and taxane-resistant cell lines.

Abbreviation: NA, not applicable.

### Immunoblotting Showed Higher EMT Protein Expression in AR^Low^/mCRPC/NEPC and Taxane-resistant AR^Low^/mCRPC/NEPC Compared with AR^High^/mCSPC Prostate Cancer Subtypes

Our RNA-seq DEGs, scRNA-seq, *in silico,* and *in vitro* flow cytometry results indicated that aggressive AR^Low^/mCRPC/NEPC and taxane-resistant AR^Low^/mCRPC/NEPC prostate cancer have greater expression of EMT markers and “stem-like” cell population. On the basis of these findings, we determined the protein expression of the top markers associated with EMT (CD44, ALDH1, Oct-4, TGFB, Nanog, and Sox2) using immunoblotting in all prostate cancer cell lines. These data indicated that the protein expression of these top EMT markers was concurrently higher in AR^Low^/mCRPC/NEPC (PC-3, PC-3M, DU145) compared with AR^High^/mCSPC (LNCaP, VCaP, 22RV1; Fig. 2C). Furthermore, consistent upregulation of these top EMT proteins was observed in taxane-resistant DUTXR and PC-3TXR compared with AR^Low^/mCRPC/NEPC (PC-3, PC-3M, DU145) and AR^High^/mCSPC (LNCaP, VCaP, 22RV1) cells (Fig. 2C). EMT proteins were also expressed in the AA (MDA-PCa-2b and RC77T/E) cell line. However, their expression level was greater compared with AR^High^/mCSPC (LNCaP, VCaP, 22RV1), and lower than AR^Low^/mCRPC/NEPC prostate cancer (PC-3, PC-3M, DU145) and taxane-resistant AR^Low^/mCRPC/NEPC (DUTXR and PC-3TXR; Fig. 2C) prostate cancer cells. Densitometry plots showed significant differential protein expression among all prostate cancer subtypes (*P* values, *P* ≤ 0.05; Fig. 2C).

### Metronomic Topotecan in Combination with DTX Reduced Cell Growth and Cell Density in AR^Low^/mCRPC/NEPC and Acquired Taxane-resistant AR^Low^/mCRPC/NEPC Prostate Cancer Subtypes

**FIGURE 3:**
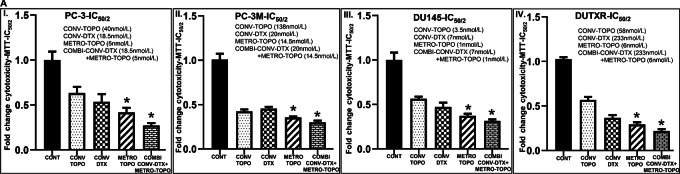

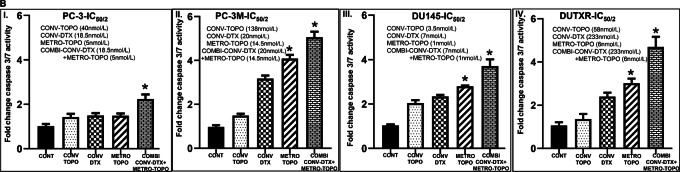

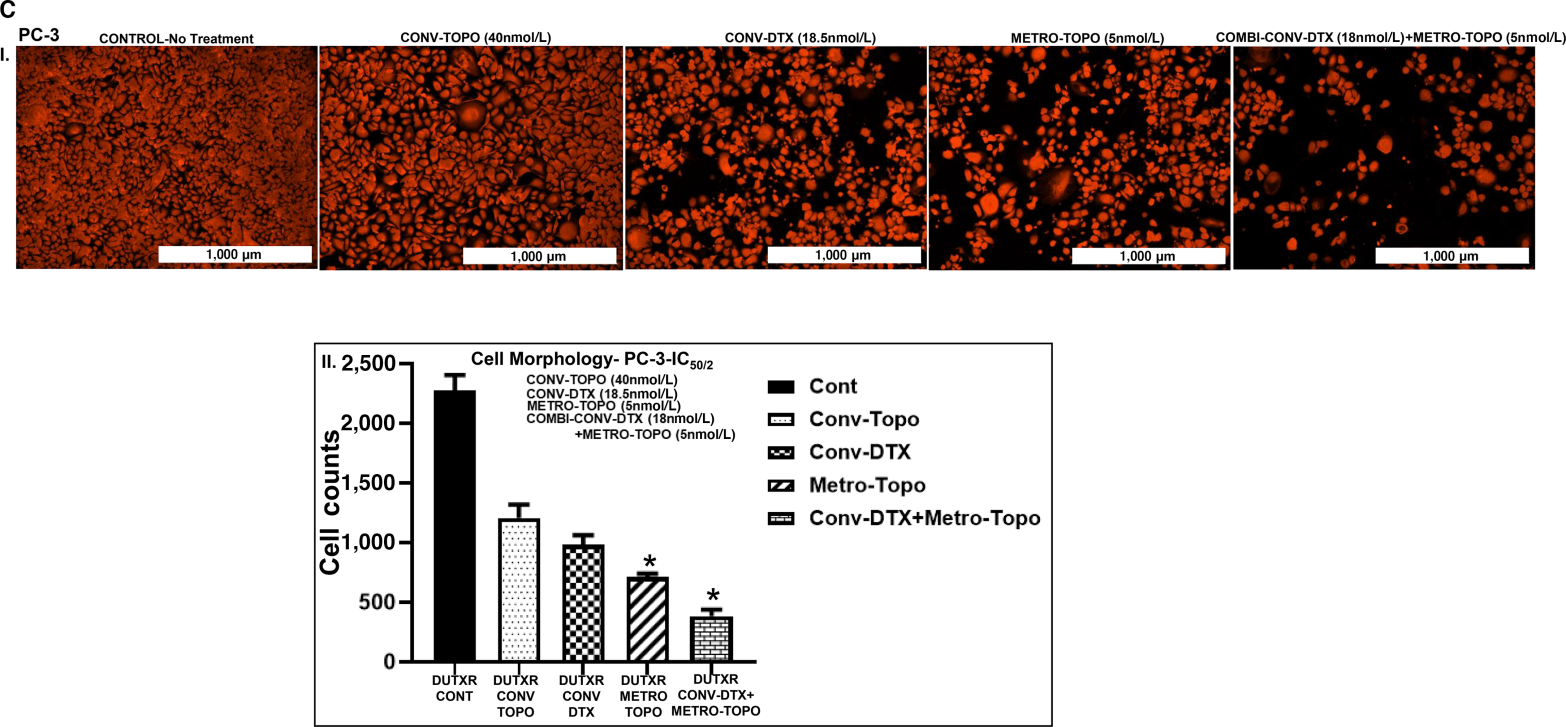
Metronomic topotecan in combination with DTX showed potency against prostate cancer subtypes, AR^Low^/mCRPC/NEPC (PC-3, PC-3M, DU145) and AR^Low^/mCSPC/NEPC^taxane resistant^ (DUTXR) cells. Cytotoxicity: *In vitro* effect of metronomic and conventional administration of topotecan on prostate cancer cell lines was assessed. **A,** MTT assay: Cytotoxicity was measured following 72 hours of CONV-TOPO, CONV-DTX, METRO-TOPO, and the combination of CONV-DTX+METRO-TOPO treatment in PC-3, PC-3M, DU145, DUTXR cell lines using mitochondrial activity [3-(4, 5-Dimethylthiazol-2-yl)-2,5-diphenyltetrazolium bromide] or the MTT assay. In all cell lines, METRO-TOPO (lower dose) reduced cell growth more compared with CONV (high dose) treatment at IC_50/2_. The greatest reduction in cell growth was observed following CONV-DTX + METRO-TOPO combination treatment in all prostate cancer cell lines tested (*, *P* ≤ 0.05). **B,** Apoptosis (caspase 3/7 assay): Levels of caspase 3/7 enzyme (a marker of apoptosis) activities measured followed CONV-TOPO, CONV-DTX, METRO-TOPO, and the combination of CONV-DTX + METRO-TOPO 72 hours treatment. Results showed significant increases in caspase 3/7 activity following each treatment compared with control. Results confirmed significantly greater treatment-induced apoptosis was observed for post-METRO-TOPO treatment compared with CONV-TOPO and DTX. The highest treatment-induced apoptosis was observed in combination with CONV-DTX+METRO-TOPO in all cell lines (*, *P* ≤ 0.05). **C,** Microscope images (Cytation5): Cell morphologic study reported micrographs of prostate cancer cells exposed to all dosing regimens showed decreases in cell density compared with control cells. Results showed that METRO-TOPO reduces higher cell density compared with CONV-TOPO and CONV-DTX. Highest cell death observed in CONV-DTX+METRO-TOPO combination treatment compared with CONV-TOPO, CONV-DTX, and METRO-TOPO single-drug treatment (*, *P* ≤ 0.05).

Previously we reported the cytotoxicity of CONV-TOPO, CONV-DTX, and METRO-TOPO for all cell lines (22, 29). In this study, the effect of CONV-TOPO, CONV-DTX, METRO-TOPO, and CONV-DTX+METRO-TOPO administration on AR^Low^/mCRPC/NEPC (PC-3, PC-3M, DU145) cell lines, were assessed by MTT assay at their estimated IC_50_ and IC_50/2_ drug concentrations (describe in Supplementary Table S5) for each drug at 72 hours. *In vitro* cytotoxicity showed decreases in cell survival or growth after 72 hours of drug treatment in all prostate cancer cell lines. In all cell lines, METRO-TOPO (lower dose) was more potent at reducing cell growth compared with CONV (high dose) treatments at IC_50_ and IC_50/2_ (Fig. 3A; Supplementary Fig. S6A). Furthermore, the greatest reduction in cell growth was observed following CONV-DTX + METRO-TOPO combination treatment in all prostate cancer cell lines tested (Fig. 3A; Supplementary Fig. S6A). The effect of CONV-TOPO, CONV-DTX, METRO-TOPO, and CONV-DTX+METRO-TOPO administration on taxane-resistant AR^Low^/mCRPC/NEPC (DURXR) prostate cancer subtypes. Results showed similar trends, greater potency was observed in METRO-TOPO and combination treatments.

The effect of METRO-TOPO on cell density and cellular morphology was determined. In agreement with MTT assays, micrographs of prostate cancer cells exposed to all dosing regimens showed decreases in cell density compared with control cells (Fig. 3C; Supplementary Fig. S6C). Treatment with METRO-TOPO resulted in a greater reduction of cell density compared with CONV-TOPO and CONV-DTX (Fig. 3C; Supplementary Fig. S6C). Importantly, we observed greater cell death in combination treatment, CONV-DTX+METRO-TOPO, compared with single drug treatments with CONV-TOPO, CONV-DTX, or METRO-TOPO (Fig. 3C; Supplementary Fig. S6C).

### Metronomic Topotecan in Combination with DTX Induced Apoptosis in AR^Low^/mCRPC/NEPC and Acquired Taxane-resistant AR^Low^/mCRPC/NEPC Prostate Cancer Subtypes

The effect of CONV-TOPO, CONV-DTX, METRO-TOPO, and CONV-DTX+METRO-TOPO administration on apoptosis was determined by assessing caspase 3/7 (a marker of apoptosis) activity. The level of apoptosis was estimated at the IC_50_ and IC_50/2_ (dose described in Supplementary Table S5) in all cell lines following 72 hours exposure in AR^Low^/mCRPC/NEPC (PC-3, PC-3M, and DU145) and acquired taxane-resistant AR^Low^/mCRPC/NEPC (DUTXR). A significant increase in caspase 3/7 activity following each treatment compared with controls (Fig. 3BI–IV). A greater increase in apoptosis was observed post-METRO-TOPO treatment (at 72 hours IC_50_ and IC_50/2_ dose for metronomic topotecan) compared with CONV-TOPO and DTX (at 72 hours IC_50_ and IC_50/2_ dose for conventional topotecan and DTX). The greatest treatment-induced apoptosis was observed in combination with CONV-DTX+METRO-TOPO (at 72 hours IC_50_ and IC_50/2_ dose for conventional DTX and IC_50/2_ dose for metronomic topotecan) in all cell lines (Fig. 3BI–IV; Supplementary Fig. S6B). The relative increase (fold change) in caspase 3/7 activity following each treatment reinforced our findings (Supplementary Table S1).

### scRNA-seq and RNA-seq Analysis Indicated Downregulation of EMT Markers in AR^Low^/mCRPC/NEPC Prostate Cancer Tumor Models Following Metronomic Topotecan Treatment

**FIGURE 4:**
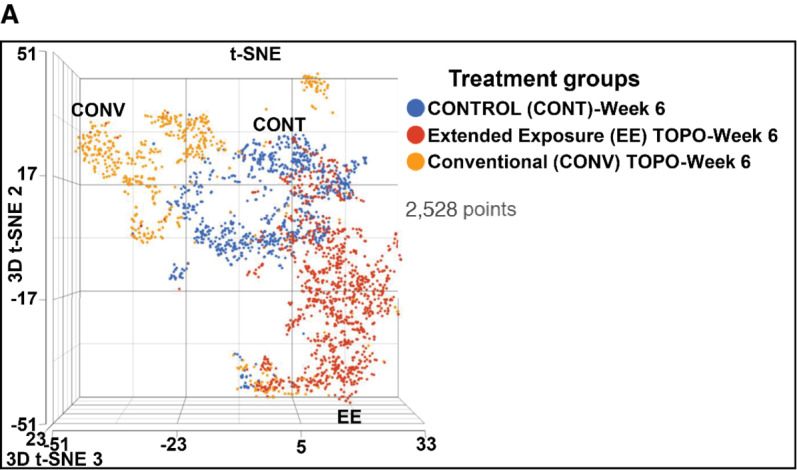

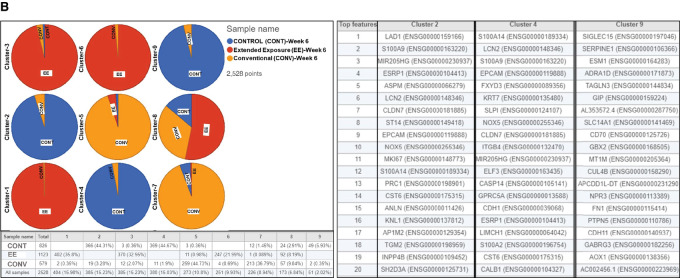

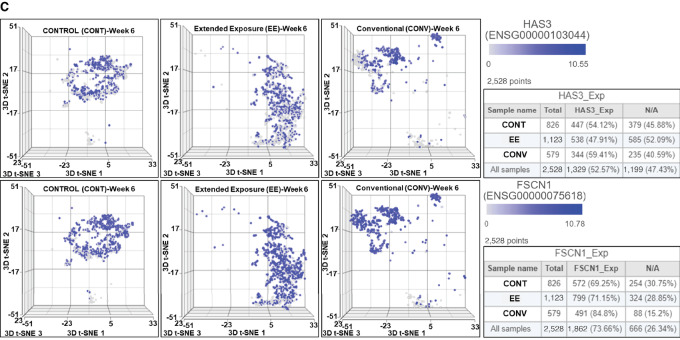

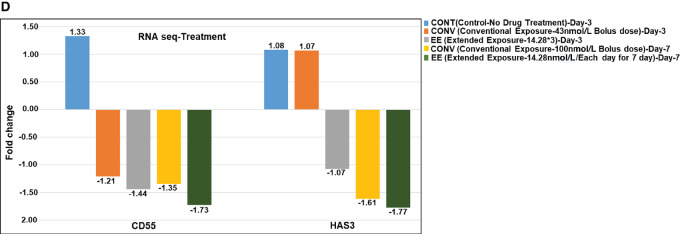
Pretreatment versus posttreatment single-cell transcriptomics and RNA-seq analysis showed extended exposure of metronomic topotecan (EE) downregulates signatures of EMT and taxane-resistant markers in AR^Low^/mCRPC/NEPC prostate cancer tumor model. scRNA-seq using the droplet sequencing method (10X Genomics) was performed on the tumor model (PC-3; 3D spheroid). Each dot represented a single cell. Doublet cells were not included. **A,** METRO-TOPO treatment effects on subclonal population distribution of AR^Low^/mCRPC tumor based on EMT markers: t-SNE plots represented the comparison between the single-cell clusters representing control (no drug treatment), 6-week EE-TOPO (extended exposure metronomic TOPO treatment) and 6-week CONV-TOPO treatment. Single-cell population for each treatment well segregated from each other, because they represented differential scRNA expression profiles. **B,** TOPO-METRO treatment reduces EMT-specific subclonal population distribution in AR^Low^/mCRPC tumors: Results exhibited nine subclonal populations (clusters) based on scRNA expression profiling in AR^Low^/mCRPC tumors. Furthermore, 6 weeks of EE-TOPO (extended exposure) reduced subclonal populations (clusters) from nine to six. Clusters two, four, and nine disappeared after 6 weeks of EE-TOPO (extended exposure) treatment, which includes EMT and drug resistance markers (*S100A9*, *ESRP1*, *ASPM*, *EPCAM*, *CLDN7*, *CDH1*, *INPP4B*, *CD70*, *FN1*, *CDH11*, *SERPINE1*, *ESM1*, *TOP2A*). **C,** METRO-TOPO treatment reduces EMT markers in AR^Low^/mCRPC single-cell subclonal populations: t-SNE plots showing the comparison in single-cell clusters among all treatment groups in AR^Low^/mCRPC/NEPC (PC-3) tumor model. Top EMT and taxane-resistant marker *HAS3* expression was downregulated by 6-week EE-TOPO (extended exposure) 54% to 47%, whereas 6-week CONV-TOPO treatment upregulated 54% to 59%. EMT marker *TGFB1* showed the same trend (Supplementary Fig. S5). Furthermore, taxane resistance marker *FSCN1* expression increased less after 6-week EE compared with CONV-TOPO treatment (69% to71% for EE-TOPO whereas 69% to 84% for CONV-TOPO). **D,** METRO-TOPO treatment reduces EMT GE in AR^Low^/mCRPC tumors (RNA-seq): RNA-sequencing analysis showed reduction (downregulation) of top EMT markers (*CD55* and *HAS3*) by 6-week EE-TOPO compared with CONV-TOPO treatment.

scRNA-seq analysis identified differential subclonal populations (clusters) representing control (no drug treatment), 6-week EE-TOPO (extended exposure of topotecan), and 6-week CONV-TOPO treatment (Fig. 4A). Further scRNA-seq analysis showed long-term EE-TOPO exposure downregulated expression of EMT markers and “stem-like” subclonal populations in the prostate cancer tumor model (Fig. 4B). We identified nine subclonal populations (clusters) based on scRNA-seq in control, whereas followed by EE-TOPO treatment, three (clusters two, four, and nine) subclonal populations (clusters) disappeared. These clusters include EMT markers (*S100A9*, *ESRP1*, *ASPM*, *EPCAM*, *CLDN7*, *CDH1*, *INPP4B*, *CD70*, *FN1*, *CDH11*) and drug metabolism or resistance markers (*SERPINE1*, *ESM1*, *TOP2A*; Fig. 4B). Further analysis showed top EMT marker *HAS3* expression was downregulated by EE-TOPO treatment (54% to 47%) compared with CONV-TOPO treatment (upregulated 54% to 59%; Fig 4C). Another EMT marker, *TGFB1*, showed similar trends (Supplementary Fig. S7). Taxane-resistance marker *FSCN1* expression increased less following EE-TOPO (69%–71%) compared with CONV-TOPO (69%–84%) treatment (Fig. 4B). RNA-seq analysis for control (no drug treatment), 3-days, and 6-week EE-TOPO versus CONV-TOPO treatment showed more reduction (downregulation) of top EMT markers, such as *CD55* and *HAS3* by EE-TOPO compared with CONV-TOPO treatment (Fig. 4D).

### Metronomic Topotecan Treatment Reduced “Stem-like” Cell Load in AR^Low^/mCRPC/NEPC Prostate Cancer Subtypes

**FIGURE 5:**
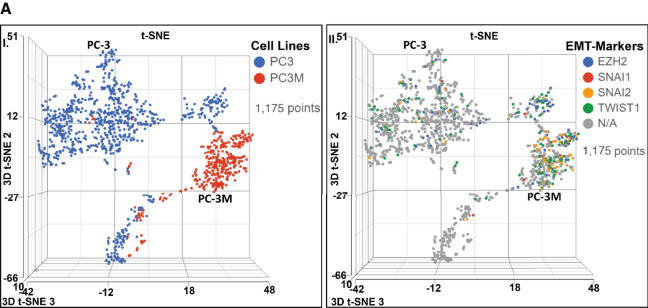

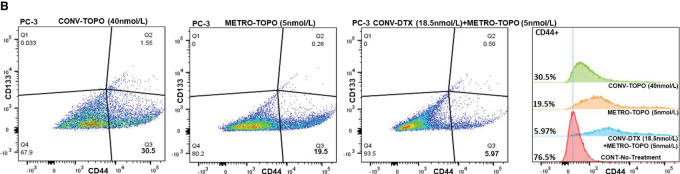

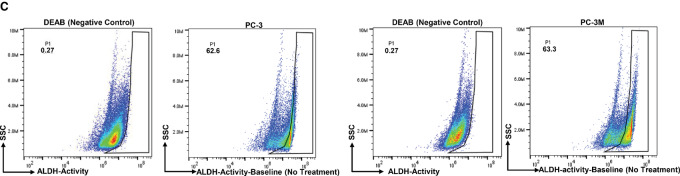

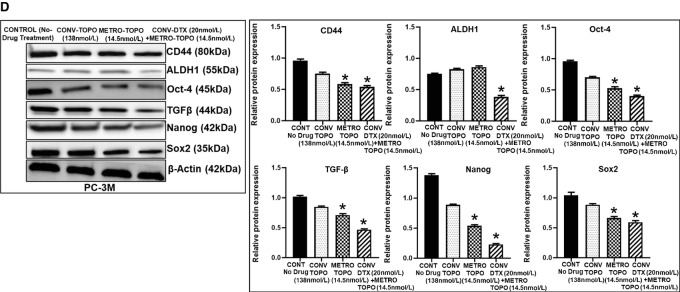

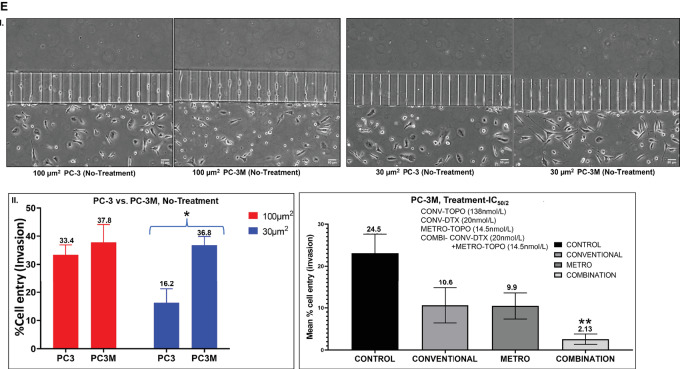
Metronomic topotecan treatment as a single agent and in combination with DTX reduces EMT/stemness in AR^Low^/mCRPC/NEPC clonally derived highly metastatic prostate cancer cell lines. **A,** Single-cell transcriptomics: Identified differential expression of EMT markers in AR^Low^/mCRPC/NEPC (PC-3 vs. PC-3M) cells. scRNA-seq using the Droplet sequencing method (10X Genomics) was performed on the prostate cancer cell lines. Each dot represents a single cell. (**I**) t-SNE plots showing the comparison between the single-cell clusters represented AR^Low^/mCRPC/NEPC (PC-3 vs. PC-3M) cells. (**II**) Top EMT transdifferentiation markers in PC-3 and PC-3M, included *EZH2*, *Snail* (*SNAI1*), *Slug* (*SNAI2*), *TWIST1* genes. *SNAI1* and *SNAl2* are markers for cell invasion. *SNSI1* and *SNSI2* increased CD44^+^ expressing cells in prostate cancer populations. All EMT markers were expressed at higher levels in PC-3M compared with PC-3 cells, which indicated more stemness and cell invasion character for PC-3M cells. **B,** Assessment of posttreated “stem-like” cells (CD44^high^/CD133^high^) population in AR^Low^/mCRPC cell lines by Flowcytometry: prostate cancer cell lines stained with stemness markers (CD44, CD133, and both CD44/133) after CONV-TOPO, METRO-TOPO, and combination (CONV-DTX+METRO-TOPO) treatment. CONV-TOPO, METRO-TOPO and combination (CONV-DTX+METRO-TOPO) treatment reduces CD44^high^ cells 30.5%, 19.5%, and 5.97%, respectively, in AR^Low^/mCRPC (PC-3) cell line. Therefore, METRO-TOPO as a single agent and in combination with DTX reduced higher percentages of “stem-like” CD44^high^ cell population compared with CONV-TOPO treatment. Data for PC-3M and DU145 cells are shown in (Supplementary Fig. S6A and S6B; Table 2). **C,** Baseline ALDH: aldehyde dehydrogenase (ALDH) was assessed using an Aldefluor kit according to the manufacturer's instructions (Stem Cell Technologies).In AR^Low^/mCRPC/NEPCP prostate cancer cell lines (PC-3 vs. PC-3M), ALDH was marginally higher in PC-3M compared with PC-3, indicating the presence of a “stem-like phenotype.” The ALDH inhibitor DEAB was used as a negative control. The cells without inhibitor shifted to the right were considered ALDH+ cells (right). **D,** Immunoblot analysis: EMT proteins were significantly downregulated in METRO-TOPO versus CONV-TOPO in AR^Low^/mCSPC/NEPC (PC-3M) prostate cancer cells. Combination treatment, CONV-DTX+METRO-TOPO exhibited the highest downregulation of EMT proteins compared with other treatments. Posttreatment protein expression downregulation was following orders: CONV-TOPO>CONV-DTX>METRO-TOPO. Beta-actin was used as a control housekeeping gene to normalize the protein expression of other genes (*, *P* ≤ 0.05). **E,** PDMS-based microchannel cell migration assay: This assay allows the study of cancer cell invasion into physically restricted spaces. **I,** Representative images showed that PC-3M cells entered confining microchannels (30 μm^2^) more effectively compared with PC-3 cells, suggesting that PC-3M cells were more invasive. However, the differential percentage of cell entry into partially confined microchannels (100 μm^2^) was not statistically significant between PC-3 and PC-3M cells (video). (**II**) Bar graphs represented the quantification of (**I**). Figure demonstrated that PC-3M was more invasive compared with PC-3 cells (*P* < 0.05). (**III**) Assessed the entry of post-drug exposure PC-3M cells into confining microchannels. Experiments showed that treatment with METRO-TOPO (lower dose) reduced cell invasion more compared with treatment with CONV-TOPO (high dose). Combination (CONV-DTX+METRO-TOPO) treatment showed highest reduction of posttreatment cell invasion compared with other treatments. ANOVA followed by multiple comparisons (**, Bonferroni *P* ≤ 0.01).

AR^Low^/mCRPC/NEPC (PC-3, PC-3M, DU145) were treated with CONV-TOPO, METRO-TOPO, and combination CONV-DTX+METRO-TOPO treatments. Pretreated and posttreated prostate cancer cell lines were stained with stemness markers CD44, CD133, and both CD44/133 antibodies. All cell lines showed a greater reduction in the CD44^high^ (“stem-like”) cell population following METRO-TOPO treatment compared with CONV-TOPO treatment. Furthermore, combination treatment reduced the highest percent of the CD44^high^ cell population (Fig 5B; Supplementary Fig. S8A and S8B; Table 2). Next, we sorted CD44^+^ cells from PC-3M and treated them with CONV-TOPO, CONV-DTX, METRO-TOPO, and combination with METRO-TOPO+CONV-DTX. Cell-sorted PC-3M-CD44^+^ cell populations showed the highest cell survival reduction following combination treatment compared with other treatments (Supplementary Fig. S9A). The caspase 3/7 assay also showed that combination treatment induced the greatest apoptosis (6.22-fold) compared with other treatments (Supplementary Fig. S9B; Supplementary Table S4).

Flow cytometry analysis identified ALDH in PC-3 and PC-3M, indicating the presence of a “stem-like phenotype” (Fig. 5C). We also showed ALDH expression was greater in PC-3M compared with PC-3. To evaluate the effect of treatment on stemness-related or EMT protein expression, we performed posttreatment immunoblotting for all treatments in the PC-3M cell line. EMT proteins (CD44, ALDH1, Oct-4, TGFB, Nanog, and Sox2) were significantly (*P* ≤ 0.05) downregulated following METRO-TOPO compared with CONV-TOPO treatment in PC-3M. Combination treatment exhibited the greatest downregulation (17%–56%) of all EMT proteins compared to other treatments (Fig. 5D; Supplementary Table S2).

### A Microfluidic Screen Showed Metronomic Topotecan Treatment is Potentially Effective Against Cell Invasion in AR^Low^/mCRPC/NEPC Prostate Cancer Subtypes

This experiment allowed us to study the effect of drug and dosing schedule as a single agent and in combination on tumor cell motility through microchannels of dimensions that mimic the size of confining pores and channel-like tracks encountered by migrating cells *in vivo* (36). Figure 5EI–II (and Supplementary Videos) showed that cell entry into confining (*W* × *H* = 3 × 10 μm^2^) microchannels was greater in PC-3M compared with PC-3, suggesting that PC3-M cells are more invasive into mechanically challenging microenvironments. For confined (*W* × *H* = 10 × 10 μm^2^) microchannels, cell entry was greater for PC-3M compared with PC-3 but the difference was not significant.

The effect of drug treatment on PC-3M migration was accessed. PC3-M entry into confining microchannels was markedly suppressed upon TOPO-METRO single-agent and TOPO-METRO+CONV-DTX combination treatment. Of note, combination therapy resulted in a higher reduction in the invasiveness of PC-3M compared with other treatments (Fig. 5EIII).

### Metronomic Topotecan in Combination with DTX Reduced Decreased “Stem-like” Cell Load in Acquired AR^Low^/mCRPC/NEPC^taxane-resistant^ Prostate Cancer Subtypes

**FIGURE 6:**
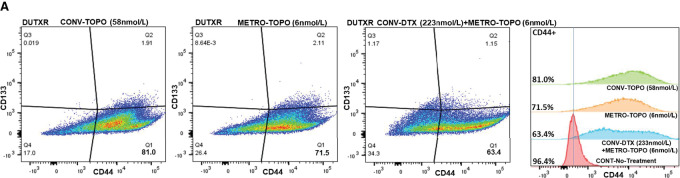

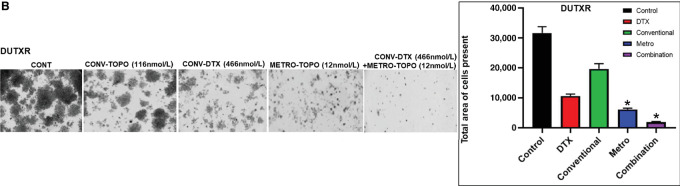

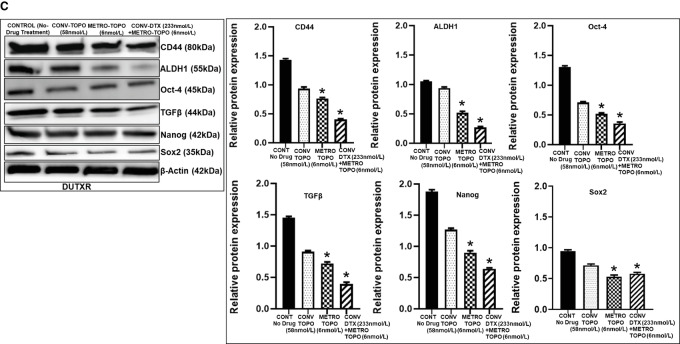

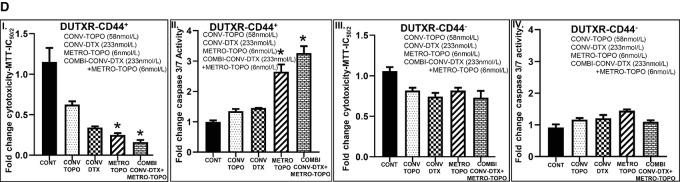

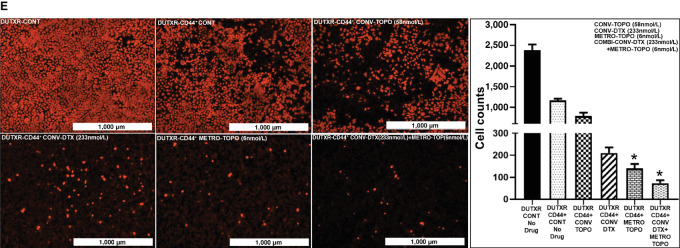
Metronomic topotecan treatment as a single agent and in combination with DTX reduces EMT/stemness in AR^Low^/mCRPC/NEPC clonally derived taxane-resistant prostate cancer subtypes. **A,** Assessment of posttreated “stem-like” cells (CD44^high^/CD133^high^) population in taxane-resistant AR^Low^/mCRPC cell lines by Flow cytometry: DUTXR cells line stained with stemness markers (CD44, CD33, and both CD44/133) after CONV-TOPO, METRO-TOPO, and combination (CONV-DTX+METRO-TOPO) treatments. CONV-TOPO, METRO-TOPO, and combination (CONV-DTX+METRO-TOPO) treatments reduced CD44^high^ cells 81.0%, 71.5%, and 63.4%, respectively, in taxane-resistant AR^Low^ mCRPC DUTXR cells. Therefore, METRO-TOPO as single agent and in combination treatment reduced the highest percentage of “stem like” cell population load (*P* < 0.05; Table 2). **B,** Colony-forming assay: Number and size of colonies were reduced after all treatments compared with control (no-drug treatment). Next, combination treatment with CONV-DTX+METRO-TOPO reduced the number and size of colonies the greatest compared with CONV-TOPO, CONV-DTX, and METRO-TOPO treatments in the DUTXR cell line. Posttreatment colony number and size reduction in the following order: CONV-TOPO>CONV-DTX>METRO-TOPO>Combination (METRO-TOPO+CONV-DTX). Therefore, METRO-TOPO as a single agent or in combination reduced highest number of the colony and size compared with other treatments. Colonies were developed for 21 days (*, *P* ≤ 0.05). **C,** Immunoblot analysis: Proteins representing top EMT markers were downregulated significantly after METRO-TOPO treatment compared with CONV-TOPO treatment in AR^Low^/mCSPC/NEPC^taxane-resistant^ (DUTXR) prostate cancer cell lines. Combination treatment (CONV-DTX+METRO-TOPO) exhibited the highest downregulation of EMT marker proteins compared with other treatments. Posttreatment protein expression downregulation was following orders: CONV-TOPO>CONV-DTX>METRO-TOPO. Beta-actin was used as a control housekeeping gene (*, *P* ≤ 0.05). **D,** Assessment of treatment effect on “stem-like” cells (CD44^high^) population in taxane-resistant AR^Low^/mCRPC cell lines by FACS: DUTXR cell line stained with stemness markers (CD44) and sorted CD44^+^ versus CD44^−^ cells, followed by CONV-TOPO, METRO-TOPO, CONV-DTX, and combination (CONV-DTX+METRO-TOPO) treatments. Next, cell cytotoxicity (MTT) and caspase 3/7 activity (apoptosis) were assessed. (**I**) Cytotoxicity profiling by MTT showed that combination (CONV-DTX+METRO-TOPO) treatment reduced highest cell survival or cell growth compared with other treatments. Posttreatment cell survival or cell growth reduction was following orders: CONV-TOPO>CONV-DTX>METRO-TOPO. (**II**) Combination (CONV-DTX+METRO-TOPO) treatment induced greater apoptosis compared with other treatments in taxane-resistant AR^Low^/mCRPC-DUTXR cell lines. (**III** and **IV**) CD44^−^ cells showed no significant differences for all treatments (*, *P* ≤ 0.05). **E,** Pretreatment and posttreatment microscope imaging: Results showed significantly higher cell death in METRO-TOPO and combination (CONV-DTX+METRO-TOPO) treatments compared with CONV treatment for both the drugs (TOPO and DTX). Images were captured by Cytation5 Cell Imaging Multimode Reader at a 1,000 μm scale. ImageJ analysis showed a significant difference in cell density for CONV versus METRO versus combination TOPO treatments (*, *P* ≤ 0.05).

The DUTXR cell line was treated with CONV-TOPO, METRO-TOPO, and combination CONV-DTX+METRO-TOPO. Pretreated and posttreated cells were stained with stemness marker antibodies CD44 and CD133 individually, and together (as described above), and analyzed by flow cytometry. These results showed a greater reduction in CD44^high^ (“stem-like”) cell populations by METRO-TOPO (81.0%) compared with CONV-TOPO treatments (71.5%). Furthermore, combination treatment showed the greatest reduction (63.4%) of the “stem-like” CD44^high^ cell populations (Fig. 6A; Table 2).

Immunoblotting results demonstrated that EMT proteins were also downregulated significantly in METRO-TOPO compared with CONV-TOPO treatment in AR^Low^/mCSPC/NEPC^taxane-resistant^ (DUTXR) cell lines. Combination treatment (CONV-DTX+METRO-TOPO) exhibited the greatest downregulation (26%–61%) of EMT proteins compared with other treatments (Fig. 6C; Supplementary Table S3).

Finally, cell-sorted DUTXR-CD44^+^ populations were treated with CONV-TOPO, METRO-TOPO, CONV-DTX, and combination (CONV-DTX+METRO-TOPO) treatments. A marked suppression in cell survival was observed in the combination treatment compared with other groups (Fig. 6DII). In addition, the caspase 3/7 assay showed that combination treatment induces the greatest level of apoptosis (3.28-fold) relative to other treatments (Fig. 6CII; Supplementary Table S4), where CD44^−^ cells showed nonsignificant differences for all treatments (Fig. 6CIII–IV; Supplementary Table S4).

Microscopic images revealed CONV-TOPO, METRO-TOPO, CONV-DTX, and combination (CONV-DTX+METRO-TOPO) treatment effect on CD44^+^ sorted cell populations. Results showed significantly higher cell death in METRO-TOPO (6%) and combination treatment (3.2%) compared with CONV-TOPO (54%) and CONV-DTX (31%) treatments (Fig. 6E).

### Metronomic Topotecan in Combination with DTX Reduced Proliferative Capacity in Acquired AR^Low^/mCRPC/NEPC^taxane-resistant^ Prostate Cancer Subtypes

Next, we evaluated the potential effect of CONV-TOPO, CONV-DTX, METRO-TOPO, and combination (METRO-TOPO+CONV-DTX) treatment on the proliferative capacity of the DUTXR cells using the colony-forming assay. TOPO-METRO treatment alone significantly reduced colony number (16.4%) as well as colony size when compared with control or CONV-TOPO (67.0%) or CONV-DTX (33.5%) treatment. Furthermore, colony-forming assay results also showed the combination of METRO-TOPO+CONV-DTX treatments further reduced the colony numbers (9.53%) and size (Fig. 6B).

## Discussion

Drug development for aggressive, lethal treatment-resistant prostate cancer poses a significant challenge with few therapeutic successes (4, 12). We used scRNA-seq and bulk RNA-seq as an approach to demonstrate that EMT and cancer “stemness” signatures are key pathways to developing metastatically aggressive prostate cancer, including castration-resistant and taxane-resistant tumors in EA and AA. As shown in Fig. 1BVI and 1CV, the intratumor heterogeneity within various cell lines was revealed by single-cell clusters (representing subclonal populations) determined by t-SNE or UMAP analysis based on the expression of top biomarkers for each subcluster. Our results showed several clusters which are unique to each prostate cancer cell line, in addition to the shared subclusters possibly representing gene signatures common to the biology of prostate cancer. Furthermore, using a comparative analysis of RNA-seq and scRNA-seq from prostate cancers, we identified overexpression of *VIM*, *AHNAK2*, *ANXA1*, *ANXA2*, *CD109*, *CD44*, *CD59*, *GPRC5A*, *HAS*, *S100A6*, *TGFBR2* in both cohorts. Importantly, the taxane-resistant gene *FSCN1* was also identified as a common gene in both cohorts of prostate cancer cell lines.

We identified “stem-like” cell populations (CD44^+^ and CD44^+^/CD133^+^) in aggressive metastatic prostate cancer subtypes, which was confirmed by the RNA-seq and subclonal overexpression of several EMT markers (*VIM*, *HAS3*, *S100A6*, *ANXA2P2*, *ANXA2*, *ANXA3*, *AHNAK2*, *LOXL2*, *TGFB*, *TGFBR2*, *UCHL1*, *CD44*, *CD55*, and *CD109*). We also identified top DEGs and subclonal populations between the taxane-sensitive and taxane-resistant prostate cancer subtypes. These data provide greater insights into the acquisition of drug resistance and possibly novel treatment targets.

Earlier, we demonstrated that METRO-TOPO single-agent treatment is effective against prostate cancer animal xenograft model and cell lines (22). Here, we have extended our previous findings and have shown that METRO-TOPO is highly synergistic in combination with CONV-DTX. We performed pretreatment versus posttreatment scRNA-seq and RNA-seq analysis, which revealed that METRO-TOPO treatment abrogates stem-like cell types (representing NEPC and EMT phenotypes) in lethal prostate cancer. METRO-TOPO also reduced acquired taxane resistance by downregulating EMT gene and protein and by reducing the “stem-like” CD44^+^ cell population. Furthermore, *in silico* validation with TCGA prostate cancer patient cohort databases established the relevance of top DEGs in patient survival. Using comparative analysis with whole-genome transcriptomics data from patients with prostate cancer, we concluded that METRO-TOPO has the potential to be clinically effective based on the reverse matching of DEGs.

Importantly, we identified *HAS3* as one of the top EMT markers in RNA-seq and scRNA-seq for aggressive prostate cancer that is downregulated by TOPO-METRO treatment. Hyaluronan (HA) is an important constituent of the stem cell niche (34, 35). CD44 is the major HA receptor and EMT marker in prostate cancer. High expression of *HAS3* (4.8-fold upregulation) resulted in the secretion of large amounts of HA bound to CD44^+^ in the “stem-like” cell population and plays a critical role in the development CSCs by regulating cell adhesion, migration, proliferation, differentiation, cancer metastasis, and multidrug-resistant (37, 38). In our study, *HA* was upregulated 4.8-fold in lethal prostate cancer subtypes. We also identified high levels of expression (4.07-fold upregulation) of *EMP1* in our aggressive prostate cancer subtypes which regulates the expression of CD44 to promote stemness (39). Furthermore, CD44 was upregulated upon *TGFB1*-induced EMT (40), and our study reported overexpression of *TGFB1* in aggressive prostate cancer (3.41-fold in GE and ∼2-fold protein). Furthermore, we identified a higher percentage of *TGFB1* overexpressed subclonal population (scRNA-seq) in aggressive taxane-resistant DUTXR compared with taxane-sensitive DU145 prostate cancer subtypes. We reported that METRO-TOPO (6-week EE) treatment reduces *TGFB1* expression in subclonal populations. On the basis of evaluation of TCGA database, the downregulation of TGFB1 is beneficial and associated with increased patient survival.

Previous studies have reported that CD44 expression is greater in prostate cancer, and involved in cancer cell proliferation, invasion, migration, and drug resistance (41). Some studies also suggested that CD44 plays a vital role in cancer stemness, specifically in prostate cancer (42, 43). We observed that CD44^+^ cells (96%) frequency and protein expression were greatest in taxane-resistant mCRPC cell lines. Furthermore, we identified a higher percentage of CD44-overexpressed subclonal population (scRNA-seq) in aggressive taxane-resistant DUTXR compared with taxane-sensitive DU145 prostate cancer subtypes. The ability to reduce stemness offers a potential strategy to treat aggressive and resistant prostate cancer. In our study, METRO-TOPO and combination (METRO-TOPO+CONV-DTX) treatment downregulated CD44^+^ (71.7% and 63.4%) cells along with protein expression (55% and 29%). We also observed high percentages of CD44^+^ cell populations in mCRPC cell lines and METRO-TOPO following single agent and in combination therapy with DTX reduced the amount of CD44^+^ cells significantly after treatment. Furthermore, METRO-TOPO treatment significantly decreased cell invasion and colony formation in aggressive and taxane-resistant forms of prostate cancer. Further studies employing CRISPR knockdown of *HAS3* (and other top genes) are needed to further explore the significance of these novel mechanisms and their impact on cancer stemness and its association with METRO-TOPO treatment-related efficacy.

Here, we have identified *VIM* as a top common EMT marker from RNA-seq and scRNA-seq for aggressive prostate cancer subtypes. Earlier studies also identified its role in invasion and metastasis in prostate cancer (16, 44–46). We also showed that METRO-TOPO (6-week EE) treatment reduces *VIM* expression in subclonal populations.

Furthermore, *SNAI1* plays a critical role in the aggressiveness of prostate cancer by increasing the expression of *CD44* and *vimentin* (47). In our study, we detected greater expression of these markers in the subclonal population (scRNA-seq analysis) and RNA-seq GE (5.42-fold upregulation) of mCRPC/NEPC. *SNAl* is associated with cell proliferation and cell invasion in prostate cancer (41). We observed high expression (scRNA-seq) of *SNAl1* and *SNAl2* in mCRPC (PC-3 and PC-3M) prostate cancer subtypes. Our microfluid-based migration assay showed high cell invasion for these prostate cancer cell lines. Furthermore, METRO-TOPO as a single agent and in combination with DTX significantly reduced cell invasion in the mCRPC cell line model.

Furthermore, *CD55* also promotes prostate cancer cell survival and metastatic lesion formation (48, 49). Recent studies have identified *CD109* as a new marker for invasive breast and prostate carcinoma (50). Our scRNA-seq and RNA-seq analysis agree with this finding. We observed greater expression of *CD55* and *CD109* (4.46- and 3.43-fold upregulation, respectively) in aggressive mCRPC/NEPC and taxane-resistant mCRPC/NEPC subtypes. We also identified a higher percentage of subclonal cell populations in aggressive taxane resistance prostate cancer subtypes overexpressing *CD55* and *CD109*.

Earlier studies reported that *ANXA3*, *ANXA2*, and its pseudogene *ANXA2P2* are overexpressed in various cancers, including prostate cancer, and facilitated transcription of the stemness genes *Nanog*, *Sox2*, and *Oct4* (51–53). *AHNAK2* upregulation is correlated significantly with advanced grades of various cancers and was associated with EMT (54). Our scRNA-seq analysis detected greater expression of *ANXA3*, *ANXA2*, *ANXA2P2*, and *AHNAK2* in aggressive prostate cancer. Our RNA-seq data also corroborated with scRNA-seq finding (4.02-, 4.51-, 4.58-, and 4.26-fold upregulated, respectively) in aggressive mCRPC/NEPC and taxane-resistant mCRPC/NEPC subtypes. We also identified greater protein expression levels of *Nanog*, *Sox2*, and *Oct4* genes in mCRPC/NEPC and taxane-resistant mCRPC/NEPC prostate cancer subtypes. METRO-TOPO downregulated Oct-4 (64%), Sox2 (60%), and Nanog (39%) in mCRPC/NEPC (PC-3M) prostate cancer cell line model. METRO-TOPO also downregulated Oct-4 (41%), Sox2 (56%), and Nanog (49%) in taxane-resistant mCRPC (DUTXR) prostate cancer cell line model. The combination of METRO-TOPO and DTX exhibited similar results. A downregulation of Oct-4 (57%), Sox2 (47%) and Nanog (17%) in mCRPC/NEPC (PC-3M), and downregulation of Oct-4 (28%), Sox2 (61%), and Nanog (35%) in taxane-resistant mCRPC/NEPC (DUTXR) prostate cancer cell line model was observed after combination treatment.

Next, *ALDH1* is another stemness marker in prostate cancer and overexpressed in mCRPC (55). Our studies also showed greater expression of ALDH1 in mCRPC/NEPC and taxane-resistant mCRPC/NEPC prostate cancer subtypes. Similarly, TOPO-METRO as a single agent and combination TOPO-METRO+CONV-DTX treatment downregulated ALDH1 protein (70%, 46%, respectively) in mCRPC/NEPC (PC-3M) cell line and (50%, 26%, respectively) in taxane-resistant mCRPC/NEPC (DUTXR) aggressive prostate cancer subtypes.

Interestingly, RNA-seq and flowcytometry data revealed that EMT markers are enriched in EA compared with AA prostate cancer cell lines. An earlier study showed *KRT80*, *RUNX*, *GATA6*, *SERPINB13*, and *SLC6a14* are important markers for prostate cancer progression (27), whereas we also identified *KRT80*, *RUNX* as well as *GATA2*, *GATA2-AS1* in our study. We also found *KRT20* is upregulated in EA compared with AA cell lines, although in future, we need further explore its significance in prostate cancer progression.

Recent studies identified *FSCN1* as a taxane-resistant marker in several solid tumors, including prostate cancer (56). We identified *FSCN1* as a key upregulated gene (4.71-fold) for taxane resistance in aggressive prostate cancer. Our results also showed that the treatment of METRO-TOPO (6-weeks EE) downregulates *FSCN1* expression in subclonal populations in aggressive mCRPC/NEPC 3D tumor models, whereas CONV-TOPO resulted in increased expression (*P* < 0.05). Furthermore, TCGA patient cohort also revealed lower expression of *FSCN1* associated with better DFS (*P* = 0.00051).

We also identified *C1orf116*, *TUBB2B* are common genes in taxane-resistant prostate cancer subtypes. *C1orf116* is a novel EMT biomarker for prostate cancer, and our study showed 17-fold and 24-fold downregulation of this gene in taxane-resistant prostate cancer subtypes DUTXR and PC-3TXR, respectively. Contrastingly, *TUBB2B* was upregulated in both taxane-resistant prostate cancer cell lines, approximately 12-fold in DUTXR and approximately 43-fold in PC-3TXR. An earlier study revealed downregulation of *C1orf116* is associated with poor prognosis in patients with lung and prostate cancer (57). *TUBB2B* is the major constituent of microtubules, the primary target for taxane-based drugs. *TUBB2B* has also been associated with DTX resistance in prostate cancer (58, 59).

Cell invasion is one of the major characteristics of EMT transition and cancer progression (60). Our microfluidic-based invasion assay supported the reduction in cell invasion potential, followed by METRO-TOPO treatments. Earlier, we demonstrated the increased potency of METRO-TOPO in animal models. However, in this study, our approach focused on *in vitro* model systems to better identify the mechanistic underpinnings of metronomic administration (two-dimensional and 3D tumor models) prostate cancer. Further preclinical validation and single-cell multi-omics strategies using CRISPR-based knockout xenograft models and patient-derived organoids/xenografts will be necessary. Our approach in this study promotes the understanding of subclonal molecular pathways underlying the differential patterns of prostate cancer aggressiveness and drug response among various prostate cancer subtypes. Overall, our study identified novel mechanisms of action that can serve as a pipeline to advance METRO-TOPO as a potent clinical-trial-ready therapeutic option for the management of lethal prostate cancer with stem-like features. These mechanisms will be key in identifying patients with molecular signatures that are sensitive to treatment, and, most importantly, can identify biomarkers that can be used to monitor treatment effectiveness and disease progression.

## Supplementary Material

Supplementary Methods Supplementary Methods

Supplementary Tables 1-7 Supplementary Tables

Supplementary Figure 1 Supplementary Fig. 1 shows Differentially expressed gene signature (DEGs) based on next gene sequencing (mRNA sequencing) for ARLow /mCRPC/NEPC (PC-3, PC-3M, DU145) vs ARHigh /mCSPC (LNCaP, VCAP, 22RV1) prostate cancer (PCa) cell lines and normal prostate cell lines RWPE1 and RWPE2. Volcano plots representing mRNA expression for all cell lines. (blue p<0.05 and gray>0.05) HeatMap representing Differentially Expressed Gene Signature (DEGs) for ARLow/mCRPC/NEPC (PC-3, PC-3M, DU145) vs ARHigh/mCSPC (LNCaP, VCAP, 22RV1) PCa and normal prostate cell lines RWPE1 and RWPE2 (FDR<0.05 and TOP-100).

Supplementary Figure 2 Supplementary Fig. 2 shows Ingenuity pathway analysis (IPA) predictions for European American/ Caucasian American (EA/CA) ARHigh/mCSPC (LNCaP, VCAP, 22RV1), ARLow/mCRPC/NEPC (PC-3, PC-3M, DU145) and African American (AA) ARHigh/mCSPC (MDA-Pca-2b) PCa cell lines as well as normal prostate cell lines RWPE1 and RWPE2.   IPA predicted A) Diseases and biological pathways for development of ARLow/mCRPC/NEPC. Major pathways are cell movement, morbidity, mortality, migration, invasion, survival, viability and development of vasculature- vasculogenesis and angiogenesis. B) Causal network pathway for development of ARLow/mCRPC/NEPC, which include oxidative phosphorylation, p70S6K, unfolded response, TREM1 signaling, NF-kB signaling, ERK5 signaling, IL-8 signaling, BAG2 signaling, Integrin signaling and VEGF signaling. C) IPA predicted HIF1α and EMT as key pathways associated with PCa development in African Americans (AA).

Supplementary Figure 3 Supplementary Fig. 3 shows Differentially Expressed Gene Signatures (DEGs) based on next-gene sequencing (mRNA sequencing) for ARLow/mCRPC/NEPC (PC-3DU145), ARLow/mCSPC/NEPC taxane resistant (DUTXR and PC-3TXR) PCa cell lines. IPA predicted key pathways based on DEGs between Taxane resistant and sensitivity. Fig. 3A. Differentially expressed gene Signature was identified among DEGs for ARLow/mCRPC/NEPC (PC-3DU145), ARLow/mCSPC/NEPC taxane-resistant (DUTXR and PC-3TXR) PCa cell lines. Venn diagrams represent unique and common DEGs for ARLow/mCRPC/NEPC (PC-3, DU145), and ARLow/mCSPC/NEPCtaxane-resistant (DUTXR and PC-3TXR) cell lines. Fig. 3B. IPA predicted key pathways based on DEGs between Taxane resistant and sensitivity. Cluster 2 (IPA predicted activation of PPARA), Cluster 4 (ERK/MAPK signaling, MSP-RON Signaling, upregulation of FOXM1, ESR1, CEBPB, XBP1, and NPM1), Cluster 6 (ATM signaling, and MYC).

Supplementary Figure 4 Supplementary Fig. 4 shows Differentially Expressed Gene Signature (DEGs) based on next-gene sequencing (mRNA sequencing) and single cell RNA sequencing for all PCa cell lines. Differentially expressed gene Signature was identified among DEGs for RNS seq and scRNAseq.

Supplementary Figure 5 Supplementary Fig. 5 shows Ingenuity pathway analysis (IPA) predictions for common DEGs among RNAseq vs. scRNAseq in all PCa cell lines IPA predicted A) Diseases and biological pathways for common DGEs. Major pathways are cell invasion, movement, neoplasia, migration, transformation, metastatic and growth B) Causal network pathway for common DEGS include LY2109761 (TGF-β receptor inhibitor), IGF2R (receptor for both insulin-like growth factor 2), IL11RA (cytokine), SEMG2 (semenogelin proteins), CCR2 (chemokine receptor type 2), TRAF3IP3 (mediates cell growth by modulating the c-Jun N-terminal kinase signal transduction pathway), GGTI-2154 (inhibitor of geranylgeranyltransferase I ), L778123 (inhibitor of FPTase and GGPTase-I), TGFB1 and CXCL5. C) upstream regulators for common DEGs were THBF1, RAF1, AGT, ER receptor, OSM, SLC15A4, JUN, and IL1B D) Canonical pathways for common DEGs were s100 family, Hepatic fibrosis, Rho family, RHOGDI and integrin singling pathway.

Supplementary Figure 6 Supplementary Fig. 6 shows Cytotoxicity Profiling of ARLow/mCRPC/NEPC (PC-3 and PC-3M) and Taxane Resistance ARLow/mCRPC (DUTXR): PC-3, PC-3M and DUTXR cell lines were treated with CONV-TOPO, METRO-TOPO, CONV-DTX and combination (CONV-DTX+METRO-TOPO) treatment, and cell cytotoxicity and caspase3/7 levels ware assed A) Cytotoxicity profiling by MTT showed combination (CONV-DTX+METRO-TOPO) reduces highest cell survival compared with other treatments for all PCa cell lines, CONV-TOPO>CONV-DTX>METRO-TOPO B) Caspase3/7 activity showed combination (CONV-DTX+METRO-TOPO) treatment reduced apoptosis the greatest compared to other treatments in all cell lines. C) Cytation5 images showed treatment effects on the cell lines PC-3M and DUTXR. Results showed significantly higher cell death in METRO compared to CONV treatment for both the cell lines and combination (CONV-DTX+METRO-TOPO) treatment reduced cell death greater for both PCa cell lines. ImageJ analysis showed significant differences in cell density for CONV-TOPO, CONV-DTX, METRO-TOPO, and combination (CONV-DTX+METRO-TOPO) treatment in PC-3, PC-3M, and DUTXR cell lines. (*p ≤ 0.05).

Supplementary Figure 7 Supplementary Fig. 7 shows Single-Cell transcriptomics; Identifies epithelial to mesenchymal transition (EMT) marker TGFB1 in ARLow/mCRPC/NEPC (PC-3 spheroid CONT-No treatment vs, extended exposure-EE and conventional exposure-CONV). Result showed TGFB1 expression decreases after EE compared to CONV treatment, which indicated EE moderated stemness in ARLow/mCRPC/NEPC. Single-cell RNA sequencing used the droplet sequencing method (10X Genomics). Each dot represents a single cell. t-distributed stochastic neighbor embedding (tSNE) plots showing the comparison between the single-cell clusters as shown.

Supplementary Figure 8 Supplementary Fig. 8 shows Flow Cytometry; Prostate cancer cell lines stained with stemness markers (CD44, CD133 and CD44/133) to determine the percent of CD44+, CD133+ and double positive cells among PCa subtypes.  Treatments with CONV-TOPO, METRO-TOPO and CONV-DTX+METRO-TOPO reduced stemness. METRO-TOPO reduced CD44+ cells to a greater extent compared to CONV-TOPO and CONV-DTX treatments, whereas combination treatment with CONV-DTX+METRO-TOPO reduced stemness (CD44+ cells) the most in ARHigh/mCRPC/NEPC. A) PC-3M, B) DU145 cell lines.

Supplementary Figure 9 Supplementary Fig. 9 shows Fluorescent-Activated Cell Sorting CD44High: PC-3M cell line stained with stemness markers (CD44) and sorted CD44+ cells, followed by CONV-TOPO, METRO-TOPO, CONV-DTX, and combination (CONV-DTX+METRO-TOPO) therapy and cell cytotoxicity and caspase3/7 levels assessed A) Cytotoxicity profiling by MTT showed combination (CONV-DTX+METRO-TOPO) reduces cell survival compared with other treatments CONV-TOPO>CONV-DTX>METRO-TOPO B) AR-mCRPC- PC-3M showed combination (CONV-DTX+METRO-TOPO) reduces levels the most of apoptosis compared to other treatments (*p<0.05).

Supplementary Video 1 Supplementary Video 1

Supplementary Video 2 Supplementary Video 2

Supplementary Video 3 Supplementary Video 3

Supplementary Video 4 Supplementary Video 4
