# Supplementary material for: Metronomic Administration of Topotecan Alone and in Combination with Docetaxel Inhibits Epithelial–mesenchymal Transition in Aggressive Variant Prostate Cancers
Source: Cancer Res Commun. 2023 Jul 19;3(7):1286–311. doi: 10.1158/2767-9764.CRC-22-0427 (PMC10355222; doi:10.1158/2767-9764.CRC-22-0427)
Supplement: Supplementary Methods [file crc-22-0427-s01.docx]

**Supplementary Materials and Methods:**

| **Chemicals/Reagents and Instruments** | **Vendor** |
| --- | --- |
| Fetal bovine serum/FBS | Thermo Fisher Scientific (Rockford, IL) |
| Trypsin | Thermo Fisher Scientific (Rockford, IL) |
| F-12K medium | Thermo Fisher Scientific (Rockford, IL) |
| EMEM medium | Thermo Fisher Scientific (Rockford, IL) |
| RPMI-1640 medium | Thermo Fisher Scientific (Rockford, IL) |
| Goat anti-mouse IgG secondary antibody | Thermo Fisher Scientific (Rockford, IL) |
| Topotecan (TOPO) (>99% purity) | 21st Century Global E-Commerce Network (East Sussex, UK) |
| Docetaxel-DTX (98% purity) | Advanced ChemBlocks (Burlingame, CA) |
| Dimethyl sulfoxide/DMSO | BioRad (Hercules CA) |
| ECL western blotting substrate | BioRad (Hercules CA) |
| 3-(4,5-dimethylthiazol-2-yl)-2,5-diphenyltetrazolium bromide/MTT | Sigma-Aldrich (St. Louis, MO) |
| Beta-actin | Sigma-Aldrich (St. Louis, MO) |
| RNAse-A | Sigma-Aldrich (St. Louis, MO) |
| Mouse anti-human antibodies | Cell Signaling Technology (Danvers, MA) |
| Flow cytometry antibodies | Cell Signaling Technology (Danvers, MA) |
| Ghost Dye Red 780 | Cell Signaling Technology (Danvers, MA) |
| CD44 (156-3C11) Mouse mAb  (PE Conjugate) #8724 | Cell Signaling Technology (Danvers, MA) |
| CD133 (A8N6N) Mouse mAb  (Alexa Fluor® 488 Conjugate) #38725 | Cell Signaling Technology (Danvers, MA) |
| Crystal violet | VWR (Radnor, PA) |
| Glassware and plasticware | VWR (Radnor, PA) |
| GenePrint 24 System | Promega |
| Nanodrop-8000 spectrophotometer | Thermo Scientific, USA |
| Agilent 2100 Bioanalyzer | Applied Biosystems, Carlsbad, CA, USA |
| Partek Flow software | Partek, Inc, USA |
| CytoFLEX LX flow cytometer | Beckman Coulter, Indianapolis, Indiana |
| Synergy Neo2 Microplate Reader | BioTek, USA |
| Caspase-Glo 3/7 kit | Promega Madison, WI |
| Agilent Cytation5 Digital  Cell Imaging System | BioTek, USA |
| Aldefluor assay kit | Stem Cell Technologies |
| RIPA Lysis and Extraction Buffer | Thermo Fisher Scientific (Rockford, IL) |
| BSA protein standard kit | Bio-Rad, CA |
| ImageLab software | Hercules CA, USA |

| **Cell lines** | | **Ethnicity** | **Characteristic** | | **Morphology** | **Tissue** | **Disease stage** | **Clinical data** | **Complete Media** |
| --- | --- | --- | --- | --- | --- | --- | --- | --- | --- |
| Human cells | RWPE1 | European American/Caucasian | AR^High^ | Normal | Epithelial | Prostate |  |  | Keratinocyte basal media supplemented with 10ng/ml epidermal growth factor (EGF) |
| Human cells | RWPE2 | European American/Caucasian | AR^High^ | Normal | Epithelial | Prostate |  |  | Keratinocyte basal media supplemented with 10ng/ml EGF |
| Human cells | LNCaP | European American/Caucasian | AR^High^ | mCSPC | Epithelial | Prostate | Left supraclavicular lymph node | From a needle aspiration biopsy of the left supraclavicular lymph node of a 50-year-old Caucasian male (blood type B+) | 10% (v/v) FBS supplemented C4-2B in RPMI1640 |
| Human cells | VCaP | European American/Caucasian | AR^High^ | mCSPC | Epithelial | Prostate | Vertebral metastasis | Vertebral bone metastasis of a patient with hormone refractory prostate cancer. It was passaged as xenografts in mice then cultured in vitro | 10% (v/v) FBS supplemented C4-2B in RPMI1640 |
| Human cells | 22RV1 | European American/Caucasian | AR^High^ | mCSPC | Epithelial | Prostate |  | Human prostate carcinoma epithelial cell line derived from a xenograft that was serially propagated in mice after castration-induced regression and relapse of the parental, androgen-dependent CWR22 xenograft. | 10% (v/v) FBS supplemented C4-2B in RPMI1640 |
| Human cells | C4-2B | European American/Caucasian | AR^High^ | mCRPC | Epithelial-like with thin processes | Prostate | Derivative subline of human prostate cancer LNCaP-derived C4-2 cells. | Human prostatic carcinoma cell line LNCaP was co-inoculated into an athymic male nude mouse with human fibroblasts derived from an osteosarcoma. The nude mouse host was castrated after 8 weeks incubation. A tumor specimen was excised after a total of 12 weeks. | 10% (v/v) FBS supplemented C4-2B in DMEM/F12 (4:1) |
| Human cells | PC-3 | European American/Caucasian | AR^Low^ | mCRPC/NEPC | Epithelial | Prostate | Adenocarcinoma; Grade IV | Established from a bone metastasis | 10% (v/v) FBS supplemented in F-12K |
| Human cells | PC-3M | European American/Caucasian | AR^Low^ | mCRPC/NEPC | Epithelial | Prostate |  | Established from a bone metastasis | 10% (v/v) FBS supplemented in F-12K |
| Human cells | DU145 | European American/Caucasian | AR^Low^ | mCRPC/NEPC | Epithelial | Prostate | Adenocarcinoma (Grade II) | Established from a brain metastasis | 10% (v/v) FBS supplemented in EMEM |
| Human cells | DUTXR | European American/Caucasian | AR^Low^ | mCRPC/NEPC/Tx Resistant | Epithelial | Prostate | Adenocarcinoma (Grade II) | Established from a brain metastasis. Paclitaxel resistance developed by using dose-escalation with taxanes over time. | 10% (v/v) FBS supplemented C4-2B in RPMI1640 |
| Human cells | PC-3TXR | European American/Caucasian | AR^Low^ | mCRPC/NEPC/Tx Resistant | Epithelial | Prostate | Adenocarcinoma; Grade IV | Established from a bone metastasis. Paclitaxel resistance developed by using dose-escalation with taxanes over time. | 10% (v/v) FBS supplemented C4-2B in RPMI1640 |
| Human cells | MDA-Pca-2b | African American | AR^High^ | mCSPC | Epithelial | Prostate |  | Established from a bone metastasis | 10% (v/v) FBS supplemented in F-12K |
| Human cells | RC77T/E | African American | AR^High^ | mCSPC | Epithelial | Prostate | Prostate cancer patient with HPV-16E6E7 | Established from a malignant tumor. The RC-77T/E cells produced tumors in SCID mice. | Keratinocyte basal media supplemented with 10ng/ml EGF |
| Human cells | RC165T | African American | AR^High^ | mCSPC | Epithelial | Prostate |  | Primary benign tissues of African American prostate cancer patients by using telomerase. | Keratinocyte basal media supplemented with 10ng/ml EGF |
| Human cells | RC43T | African American | AR^Low^ | mCRPC | Epithelial | Prostate |  |  | Keratinocyte basal media supplemented with 10ng/ml EGF |

**Human Prostate Cancer Cell Lines**
