# Supplementary material for: Metronomic Administration of Topotecan Alone and in Combination with Docetaxel Inhibits Epithelial–mesenchymal Transition in Aggressive Variant Prostate Cancers
Source: Cancer Res Commun. 2023 Jul 19;3(7):1286–311. doi: 10.1158/2767-9764.CRC-22-0427 (PMC10355222; doi:10.1158/2767-9764.CRC-22-0427)
Supplement: Supplementary Tables 1-7 — Supplementary Tables [file crc-22-0427-s02.docx]

| **Cell** | **Dose** | **CONT** | **CONV-TOPO** | **CONV-DTX** | **METRO-TOPO** | **COMBINATION**  **(CONV-DTX + METRO-TOPO)** |
| --- | --- | --- | --- | --- | --- | --- |
| **DUTXR** | IC_50_ | 1 | 1.84 | 2.08 | 2.79* | 4.78* |
| **PC-3** | IC_50_ | 1 | 1.98 | 2.04 | 2.40 | 2.61* |
| **PC-3M** | IC_50_ | 1 | 1.53 | 3.26 | 4.20* | 5.20* |
| **DUTXR** | IC_50/2_ | 1 | 1.28 | 2.26 | 2.84* | 4.42* |
| **PC-3** | IC_50/2_ | 1 | 1.41 | 1.48 | 1.47* | 2.21 |
| **PC-3M** | IC_50/2_ | 1 | 2.92 | 3.37 | 3.62* | 4.02* |
| **DU145** | IC_50/2_ | 1 | 1.97 | 2.26 | 2.70* | 3.58* |

**Supplementary Table 1. Apoptosis assay:** the level of caspase3/7 enzyme activity was measured following CONV-TOPO, CONV-DTX, METRO-TOPO, and a combination of CONV-DTX + METRO-TOPO 72h treatment. METRO-TOPO treatment exhibited greater apoptosis than CONV-TOPO and CONV-DTX treatment in the AR^Low^/mCRPC/NEPC cell lines (PC-3, PC-3M, DU145) and AR^Low^/mCSPC/NEPC^taxane-resistant^ (DUTXR) cell lines. Further, CONV-DTX + METRO-TOPO treatment showed the greatest levels of apoptosis (caspase3/7 activity) compared to other treatments among all PCa cell lines tested. (**p* ≤ 0.05).

| **GENE/**  **PROTEIN** | **CONT**  (FOLD) | **CONT**  (%) | **CONV-TOPO** (FOLD) | **CONV-TOPO**  (%) | **METRO-TOPO** (FOLD) | **METRO-TOPO**  (%) | **CONV-DTX + METRO-TOPO** (FOLD) | **CONV-DTX + METRO-TOPO** (%) |
| --- | --- | --- | --- | --- | --- | --- | --- | --- |
| **CD44** | 1 | 100 | 0.78 | 78 | 0.61* | 61* | 0.56* | 56* |
| **ALDH1** | 1 | 100 | 0.83 | 83 | 0.70 | 70 | 0.46* | 46* |
| **4-Oct** | 1 | 100 | 0.85 | 85 | 0.64* | 64* | 0.57* | 57* |
| **TGF-β** | 1 | 100 | 0.73 | 73 | 0.55* | 55* | 0.42* | 42* |
| **Sox2** | 1 | 100 | 0.82 | 82 | 0.60* | 60* | 0.47* | 47* |
| **Nanog** | 1 | 100 | 0.64 | 65 | 0.39* | 39* | 0.17* | 17* |

**Supplementary Table 2. Immunoblot analysis;** Proteins representing top DEGs for EMT were significantly downregulated in METRO-TOPO vs CONV-TOPO in the AR^Low^/mCSPC/NEPC (PC-3M) PCa cell line. CONV-DTX+METRO-TOPO exhibited the highest percent of EMT protein downregulation/reduction compared with other treatments: CONV-TOPO>CONV-DTX>METRO-TOPO. Beta Actin was used as a control housekeeping gene (**p* ≤ 0.05).

| **GENE/**  **PROTEIN** | **CONT**  (FOLD) | **CONT**  (%) | **CONV-TOPO** (FOLD) | **CONV-TOPO**  (%) | **METRO-TOPO** (FOLD) | **METRO-TOPO**  (%) | **CONV-DTX + METRO-TOPO** (FOLD) | **CONV-DTX + METRO-TOPO** (%) |
| --- | --- | --- | --- | --- | --- | --- | --- | --- |
| **CD44** | 1 | 100 | 0.67 | 67 | 0.55* | 55* | 0.29* | 29* |
| **ALDH1** | 1 | 100 | 0.92 | 92 | 0.50* | 50* | 0.26* | 26* |
| **4-Oct** | 1 | 100 | 0.56 | 56 | 0.41* | 41* | 0.28* | 28* |
| **TGF-β** | 1 | 100 | 0.64 | 64 | 0.51* | 51* | 0.28* | 28* |
| **Sox2** | 1 | 100 | 0.75 | 75 | 0.56* | 56* | 0.61* | 61* |
| **Nanog** | 1 | 100 | 0.69 | 69 | 0.49* | 49* | 0.35* | 35* |

**Supplementary Table 3. Immunoblot analysis;** Proteins representing top DEGs for EMT were significantly downregulated in METRO-TOPO vs. CONV-TOPO in the AR^Low^/mCSPC/NEPC^taxane0-resistant^ (DUTXR) PCa cell lines. CONV-DTX+METRO-TOPO treatment exhibited the highest percent of EMT protein downregulation/reduction compared with other treatments: CONV-TOPO>CONV-DTX>METRO-TOPO. Beta Actin was used as a control housekeeping gene (*p* ≤ 0.05).

| **Cell** | **Dose** | **CONT** | **CONV-TOPO** | **CONV-DTX** | **METRO-TOPO** | **COMBINATION (CONV-DTX + METRO-TOPO)** |
| --- | --- | --- | --- | --- | --- | --- |
| **DUTXR/44+** | IC_50/2_ | 1 | 1.36 | 1.46 | 2.66* | 3.28* |
| **PC-3M/44+** | IC_50/2_ | 1 | 1.35 | 3.52 | 4.41* | 6.22* |
| **DUTXR/44-** | IC_50/2_ | 1 | 1.27 | 1.32 | 1.58 | 1.19 |

**Supplementary Table 4. Fluorescence-Activated Cell Sorting (FACS) CD44^high^:** DUTXR and PC-3M cell line stained with stemness markers (CD44) and sorted CD44^+^ *vs.* CD44^-^, followed by CONV-TOPO, METRO-TOPO, CONV-DTX and combination (CONV-DTX+METRO-TOPO) treatment and then caspase3/7 level was assessed. AR^Low^/mCSPC/NEPC PC-3M and taxane-resistant AR^Low^/mCRPC DUTXR showed that combination (CONV-DTX+METRO-TOPO) treatment reduced the level of apoptosis the most compared to other treatments. CD44^-^ cells (DUTXR) showed no significant differences for all treatments.

| **Cell line** | **PC-3** | **PC-3M** | **DU145** | **DUTXR** |
| --- | --- | --- | --- | --- |
| Ethnicity | Caucasian | Caucasian | Caucasian | Caucasian |
| Characteristic | Androgen-independent | Androgen-independent | Androgen-independent | Androgen-independent |
| IC_50_-MTT-CONV-TOPO-72 h | 93.5 ± 28 (80.0 nM)* | 276 ± 15 (276.0 nM)* | 7.0 ± 1  (7.0 nM)* | 116 ± 6.15 (116 nM)* |
| IC_50_-MTT-METRO-TOPO-72 h | 9.30 ± 1.50 (10.0 nM)* | 29 ± 3  (29.0 nM)* | 2.0 ± 0.07 (1.0 nM)* | 12 ± 1.43  (12.0 nM)* |
| IC_50_-MTT-CONV-DTX-48 h | 36.8 ± 3.03 (37.0nM)* | 39.68 ± 3.24 (40.0 nM)* | 13.5 ± 0.78 (14.0 nM)* | 466 ± 16.6 (466 nM)* |
| IC_50/2_-MTT-CONV-TOPO-72 h | 40.0 nM | 138 nM | 3.5 nM | 58.0 nM |
| IC_50/2_-MTT-METRO-TOPO-72 h | 5.0 nM | 14.5 nM | 1.0 nM | 6.0 nM |
| IC_50/2_-MTT-CONV-DTX-48 h | 18.5 nM | 20.0 nM | 7.0 nM | 233 nM |

**Supplementary Table 5. *In vitro* Cytotoxicity profiling (IC_50_ and IC_50/2_ values for MTT assays):** Data presented (nM) as mean ± SEM of at least three independent studies (n = 5/study). IC_50_ and IC_50/2 doses_ for each cell line and time point were used for assessing the cytotoxicity effect of these doses in PCa by *In Vitro* Cytotoxicity Assays**,** Caspase-3/7 Activity (apoptosis level), post treatment-Cellular Morphology study. The effect of these drugs and dose on protein expression was also measured by Immunoblotting after post-treatment by using an IC_50/2_ dose for each cell line. Additionally, the effect of these drugs on cell migration was assessed by microfluidic migration assay after post-treatment by using IC_50/2_ dose for PC-3M PCa cell line. The effect of these drugs on cancer stem-like cells (CSCs) was assessed by colony forming assay, Flow Cytometry, and Fluorescence-Activated Cell Sorting assay after post-treatment by using IC_50/2_ dose for each PCa cell line. For CONV treatment drug was added as a one-time single dose on day 0 and METRO treatment was given daily for 3 days (0, 24, and 48 h).

* Rounded number based on previous experiment and analysis. We used these doses for further experiment.

| **Gene Name** | **P-value (DUTXR vs DU145)** | **FDR (DUTXR vs DU145)** | **Fold change (DUTXR vs DU145)** |
| --- | --- | --- | --- |
| **AASS** | 2.40E-03 | 1.42E-02 | 17.10 |
| **ADAM22** | 2.87E-38 | 4.86E-36 | 43.78 |
| **AKR1C2** | 4.75E-03 | 2.51E-02 | 15.59 |
| **AKR1C3** | 5.24E-08 | 9.35E-07 | 45.00 |
| **ALDOC** | 6.90E-09 | 1.43E-07 | 35.80 |
| **ARHGEF40** | 4.11E-09 | 8.75E-08 | 15.77 |
| **ARID5B** | 2.20E-04 | 1.79E-03 | 22.18 |
| **ATP10A** | 6.44E-14 | 2.34E-12 | -23.48 |
| **ATP8A1** | 2.44E-07 | 3.86E-06 | 35.64 |
| **C1orf116** | 5.79E-06 | 6.81E-05 | -17.83 |
| **C3** | 4.43E-03 | 2.37E-02 | -16.96 |
| **CA11** | 1.54E-03 | 9.67E-03 | 16.17 |
| **CAMK4** | 5.91E-03 | 3.00E-02 | 14.15 |
| **CDH1** | 3.19E-13 | 1.09E-11 | -24.44 |
| **CDH3** | 2.54E-32 | 3.11E-30 | -24.33 |
| **CLDN11** | 1.41E-06 | 1.90E-05 | -19.87 |
| **CLIC2** | 2.85E-03 | 1.64E-02 | 18.54 |
| **COLGALT2** | 9.01E-07 | 1.26E-05 | 15.88 |
| **CORO2B** | 3.99E-03 | 2.17E-02 | -15.26 |
| **CRISPLD1** | 3.29E-08 | 6.08E-07 | 29.59 |
| **CROT** | 3.17E-29 | 3.41E-27 | 39.42 |
| **DSC3** | 9.21E-23 | 6.95E-21 | 18.64 |
| **EFEMP1** | 1.57E-46 | 3.48E-44 | -12.52 |
| **EGF** | 5.82E-05 | 5.49E-04 | 34.77 |
| **ELAPOR2** | 3.31E-27 | 3.23E-25 | 15.43 |
| **EPHB3** | 7.55E-07 | 1.07E-05 | 14.92 |
| **EPOR** | 9.57E-06 | 1.08E-04 | 37.14 |
| **F2RL2** | 2.95E-08 | 5.47E-07 | 18.95 |
| **FAM167A** | 2.21E-08 | 4.22E-07 | -28.09 |
| **FAM43A** | 4.13E-03 | 2.24E-02 | 17.86 |
| **FAM83A** | 1.34E-38 | 2.29E-36 | -26.14 |
| **FBN1** | 2.37E-20 | 1.50E-18 | 24.06 |
| **FLRT2** | 7.23E-03 | 3.54E-02 | 15.07 |
| **FOS** | 1.70E-11 | 4.71E-10 | 16.53 |
| **FOXC1** | 1.02E-03 | 6.81E-03 | 14.09 |
| **FOXRED2** | 5.11E-03 | 2.66E-02 | -17.52 |
| **FZD1** | 1.24E-22 | 9.22E-21 | 12.13 |
| **GALNT3** | 3.31E-03 | 1.86E-02 | -12.25 |
| **GCNT3** | 2.12E-08 | 4.06E-07 | 14.56 |
| **GJA3** | 1.56E-03 | 9.76E-03 | 16.13 |
| **GNAO1** | 1.19E-04 | 1.05E-03 | 30.08 |
| **HHIPL2** | 1.83E-05 | 1.93E-04 | 21.59 |
| **ICAM1** | 1.20E-13 | 4.26E-12 | -24.17 |
| **IFI44** | 1.47E-03 | 9.27E-03 | 22.21 |
| **ILDR2** | 5.91E-06 | 6.94E-05 | 21.85 |
| **IRX3** | 5.40E-03 | 2.78E-02 | 15.44 |
| **KAZALD1** | 9.01E-05 | 8.09E-04 | 14.36 |
| **KIF26B** | 4.91E-05 | 4.76E-04 | -20.38 |
| **KRT7** | 1.72E-137 | 2.12E-134 | -91.46 |
| **KRT8** | 5.84E-83 | 3.37E-80 | -155.33 |
| **LAMC2** | 7.51E-41 | 1.35E-38 | -15.94 |
| **LIPH** | 1.16E-04 | 1.02E-03 | 19.66 |
| **LMX1B** | 4.85E-03 | 2.55E-02 | 14.15 |
| **MAL2** | 1.57E-25 | 1.38E-23 | -14.27 |
| **MAP2** | 1.69E-10 | 4.24E-09 | 13.45 |
| **MCOLN2** | 7.16E-03 | 3.52E-02 | 13.38 |
| **MEGF9** | 2.16E-67 | 8.88E-65 | 13.31 |
| **MERTK** | 7.32E-07 | 1.05E-05 | 12.04 |
| **MGLL** | 1.55E-34 | 2.14E-32 | -18.50 |
| **MUC1** | 2.11E-19 | 1.23E-17 | -27.77 |
| **NT5E** | 5.52E-20 | 3.37E-18 | -12.19 |
| **PADI2** | 1.71E-07 | 2.77E-06 | -19.89 |
| **PDGFD** | 3.76E-06 | 4.61E-05 | 16.69 |
| **PDLIM2** | 8.86E-08 | 1.52E-06 | -47.40 |
| **PELI2** | 9.70E-04 | 6.50E-03 | 17.09 |
| **PITPNM3** | 3.95E-07 | 6.00E-06 | -17.85 |
| **PLD1** | 5.11E-05 | 4.92E-04 | 13.26 |
| **PLEKHG1** | 1.99E-03 | 1.21E-02 | 12.71 |
| **PPM1K** | 7.83E-04 | 5.42E-03 | 12.38 |
| **PRUNE2** | 2.45E-10 | 6.03E-09 | 34.30 |
| **RAP1GAP** | 1.77E-08 | 3.43E-07 | 13.07 |
| **RHOU** | 1.88E-06 | 2.46E-05 | 18.57 |
| **RSPO3** | 9.06E-09 | 1.83E-07 | 13.54 |
| **RUNDC3B** | 1.33E-05 | 1.46E-04 | 39.91 |
| **S1PR5** | 1.47E-04 | 1.26E-03 | 14.13 |
| **SAMD5** | 4.83E-07 | 7.18E-06 | 15.28 |
| **SCNN1A** | 1.80E-04 | 1.50E-03 | -35.64 |
| **SDC2** | 1.57E-08 | 3.07E-07 | 78.03 |
| **SELENOP** | 3.29E-04 | 2.54E-03 | 16.27 |
| **SEMA7A** | 2.57E-06 | 3.28E-05 | -24.34 |
| **SESN3** | 6.53E-28 | 6.49E-26 | 46.60 |
| **SLC16A3** | 2.59E-19 | 1.50E-17 | -13.80 |
| **SLC25A40** | 2.39E-52 | 6.66E-50 | 12.51 |
| **SLC43A3** | 5.71E-03 | 2.91E-02 | -15.73 |
| **SPNS2** | 4.94E-03 | 2.60E-02 | -12.82 |
| **SPOCK2** | 3.75E-06 | 4.60E-05 | -40.26 |
| **SRI** | 1.91E-86 | 1.18E-83 | 17.89 |
| **ST14** | 2.54E-06 | 3.26E-05 | -45.83 |
| **SUSD2** | 3.33E-14 | 1.25E-12 | -25.05 |
| **TACSTD2** | 7.78E-14 | 2.82E-12 | -34.59 |
| **TAGLN** | 2.43E-116 | 2.00E-113 | -23.97 |
| **TAS1R3** | 9.95E-05 | 8.87E-04 | 13.75 |
| **TGFBR3** | 4.07E-13 | 1.37E-11 | 23.18 |
| **TGM2** | 5.24E-68 | 2.26E-65 | -34.89 |
| **TKTL1** | 1.96E-03 | 1.19E-02 | 19.20 |
| **TNFAIP3** | 2.93E-10 | 7.12E-09 | -19.37 |
| **TP53TG1** | 2.43E-04 | 1.95E-03 | 13.02 |
| **TRANK1** | 2.35E-03 | 1.39E-02 | 13.93 |
| **TUBB2B** | 1.49E-03 | 9.42E-03 | 12.15 |
| **TXNIP** | 5.68E-19 | 3.22E-17 | 18.97 |
| **ZNF467** | 1.86E-04 | 1.55E-03 | 14.02 |

**Supplementary Table 6A. Top differentially expressed genes (DEGs) for Taxane-Resistant AR^Low^/mCRPC/NEPC (DUTXR) *vs.* mCSPC/AR^low^ (DU145) PCa cell lines.**

Gene expression was assessed by Next-generation RNA sequencing.

| **Gene Name** | **P-value (PC3TXR vs PC3)** | **FDR (PC3TXR vs PC3)** | **Fold change (PC3TXR vs PC3)** |
| --- | --- | --- | --- |
| **ACKR3** | 5.10E-06 | 4.14E-05 | 28.30 |
| **ADAM8** | 4.26E-09 | 5.68E-08 | 138.01 |
| **AFAP1-AS1** | 5.01E-07 | 4.87E-06 | 41.08 |
| **AKT3** | 1.72E-07 | 1.80E-06 | 124.22 |
| **ANGPTL2** | 2.84E-04 | 1.59E-03 | 36.50 |
| **APOBEC3G** | 1.84E-07 | 1.91E-06 | 49.78 |
| **ARMCX6** | 1.66E-05 | 1.22E-04 | 31.85 |
| **AXL** | 3.98E-34 | 2.85E-32 | 63.47 |
| **BMF** | 3.68E-11 | 6.35E-10 | 63.18 |
| **C1orf116** | 1.49E-06 | 1.33E-05 | -24.81 |
| **C5** | 1.10E-08 | 1.39E-07 | -46.04 |
| **CACNA1H** | 6.47E-06 | 5.16E-05 | 31.55 |
| **CCN1** | 4.98E-199 | 3.44E-196 | 27.97 |
| **CDK18** | 2.63E-05 | 1.86E-04 | 35.26 |
| **CEACAM5** | 1.02E-10 | 1.66E-09 | -57.84 |
| **CEBPB** | 6.41E-53 | 8.21E-51 | 34.31 |
| **CLSTN3** | 9.46E-07 | 8.72E-06 | 24.02 |
| **CNIH3** | 9.86E-07 | 9.06E-06 | 29.84 |
| **COL4A1** | 2.23E-19 | 7.72E-18 | 26.29 |
| **COL5A1** | 5.58E-05 | 3.64E-04 | 48.41 |
| **CP** | 2.51E-05 | 1.79E-04 | -24.22 |
| **CPNE2** | 7.42E-05 | 4.70E-04 | 41.64 |
| **CPVL** | 1.33E-08 | 1.66E-07 | 43.80 |
| **CREB3L1** | 2.66E-07 | 2.70E-06 | -85.66 |
| **CSF1** | 1.03E-14 | 2.51E-13 | 34.65 |
| **CYP2S1** | 4.80E-05 | 3.18E-04 | 28.00 |
| **DOK4** | 9.56E-04 | 4.64E-03 | 24.79 |
| **DPP4** | 2.02E-10 | 3.20E-09 | -73.96 |
| **DUSP10** | 6.22E-04 | 3.19E-03 | 25.63 |
| **EEF1A2** | 1.28E-55 | 1.73E-53 | 29.99 |
| **EMP2** | 4.21E-04 | 2.25E-03 | 24.47 |
| **ETNK2** | 2.80E-06 | 2.37E-05 | 24.11 |
| **FBXO32** | 1.66E-21 | 6.75E-20 | 26.57 |
| **FCGRT** | 1.41E-08 | 1.75E-07 | 128.25 |
| **FLNC** | 3.13E-10 | 4.82E-09 | 216.20 |
| **FRMD4A** | 3.33E-04 | 1.83E-03 | 25.78 |
| **FZD2** | 7.50E-10 | 1.10E-08 | 29.60 |
| **GDF15** | 0.00E+00 | 0.00E+00 | 28.02 |
| **GNAL** | 9.86E-07 | 9.06E-06 | 26.88 |
| **GOLGA8A** | 7.07E-12 | 1.32E-10 | -33.14 |
| **GRIN2D** | 6.75E-04 | 3.42E-03 | 27.08 |
| **ICAM1** | 2.29E-25 | 1.13E-23 | 54.84 |
| **IGFBP5** | 2.36E-07 | 2.42E-06 | 159.05 |
| **IL1B** | 2.09E-42 | 2.02E-40 | 83.52 |
| **IL27RA** | 2.27E-06 | 1.95E-05 | 26.75 |
| **INHBA** | 4.99E-15 | 1.27E-13 | 308.24 |
| **ITGB3** | 4.41E-17 | 1.31E-15 | 290.62 |
| **KCNG1** | 1.52E-10 | 2.44E-09 | 91.07 |
| **KIFC3** | 2.59E-09 | 3.55E-08 | 105.28 |
| **KREMEN1** | 9.03E-06 | 7.01E-05 | 53.61 |
| **KRT7** | 5.37E-13 | 1.13E-11 | 374.90 |
| **KRT81** | 7.24E-141 | 3.68E-138 | 76.65 |
| **L1CAM** | 4.76E-06 | 3.88E-05 | 28.59 |
| **LAMP3** | 1.01E-04 | 6.22E-04 | 25.81 |
| **LCN2** | 2.96E-08 | 3.47E-07 | 126.76 |
| **LDOC1** | 1.71E-08 | 2.10E-07 | 72.10 |
| **LY6E** | 3.68E-17 | 1.10E-15 | 39.35 |
| **MALAT1** | 4.14E-54 | 5.34E-52 | -43.52 |
| **MELTF** | 1.95E-13 | 4.25E-12 | 53.77 |
| **MYH15** | 1.01E-04 | 6.26E-04 | -24.50 |
| **NCK2** | 8.02E-07 | 7.48E-06 | 63.87 |
| **NRAS** | 1.98E-07 | 2.05E-06 | -29.17 |
| **NUP210** | 5.64E-11 | 9.52E-10 | 48.23 |
| **OAS2** | 2.81E-08 | 3.32E-07 | -34.72 |
| **OSER1-DT** | 5.86E-04 | 3.03E-03 | 30.45 |
| **PKIG** | 7.71E-15 | 1.89E-13 | 45.46 |
| **PLCXD3** | 1.59E-10 | 2.56E-09 | -91.38 |
| **PRAME** | 1.48E-06 | 1.32E-05 | 46.98 |
| **PTGES** | 2.35E-06 | 2.01E-05 | 82.85 |
| **PTGFR** | 7.93E-07 | 7.42E-06 | -27.97 |
| **RGS14** | 2.36E-04 | 1.35E-03 | 24.48 |
| **RN7SL1** | 2.86E-63 | 4.62E-61 | -24.77 |
| **RNA18SN1** | 0.00E+00 | 0.00E+00 | -58.80 |
| **RNA18SN2** | 0.00E+00 | 0.00E+00 | -58.80 |
| **RNA18SN3** | 0.00E+00 | 0.00E+00 | -58.80 |
| **RNA18SN4** | 0.00E+00 | 0.00E+00 | -58.80 |
| **RNA18SN5** | 0.00E+00 | 0.00E+00 | -58.80 |
| **RPLP0P2** | 8.01E-04 | 3.98E-03 | 24.09 |
| **RTN4RL2** | 3.53E-31 | 2.20E-29 | 46.94 |
| **RUNX2** | 3.56E-05 | 2.44E-04 | 53.33 |
| **S100A2** | 5.29E-32 | 3.45E-30 | -29.65 |
| **S100A9** | 5.69E-06 | 4.58E-05 | 29.17 |
| **S100P** | 1.80E-12 | 3.59E-11 | 24.04 |
| **SCIN** | 3.96E-05 | 2.68E-04 | -46.65 |
| **SHH** | 2.47E-08 | 2.95E-07 | 49.11 |
| **SIRPB1** | 6.51E-11 | 1.09E-09 | 91.93 |
| **SLC1A3** | 6.22E-20 | 2.25E-18 | 32.14 |
| **SLC43A3** | 3.52E-05 | 2.42E-04 | 29.60 |
| **SLC44A2** | 5.92E-12 | 1.12E-10 | 35.00 |
| **SPINT1** | 2.17E-19 | 7.52E-18 | 27.50 |
| **SRPX** | 5.54E-08 | 6.28E-07 | 37.36 |
| **SSTR5-AS1** | 2.22E-08 | 2.67E-07 | -112.73 |
| **SYNPO** | 6.97E-11 | 1.16E-09 | 50.91 |
| **TALAM1** | 9.28E-07 | 8.58E-06 | -67.98 |
| **TGFB2** | 1.25E-57 | 1.78E-55 | 25.42 |
| **TGM2** | 1.46E-36 | 1.12E-34 | 759.10 |
| **TINAGL1** | 1.40E-25 | 7.04E-24 | 50.28 |
| **TMC6** | 2.39E-10 | 3.75E-09 | 30.84 |
| **TMED7-TICAM2** | 3.95E-07 | 3.90E-06 | 25.33 |
| **TNS4** | 2.78E-11 | 4.91E-10 | -117.57 |
| **TNXB** | 1.37E-21 | 5.58E-20 | -48.86 |
| **TSC22D3** | 1.46E-10 | 2.35E-09 | 51.44 |
| **TSPAN33** | 2.05E-05 | 1.49E-04 | 42.48 |
| **TTN** | 3.82E-07 | 3.79E-06 | -32.84 |
| **TUBB2B** | 1.94E-08 | 2.36E-07 | 41.48 |
| **TUBB4A** | 7.11E-07 | 6.71E-06 | 115.08 |
| **TYMP** | 5.21E-05 | 3.41E-04 | 31.06 |
| **WWC3** | 7.83E-08 | 8.65E-07 | 77.78 |

**Supplementary Table 6B. Top differentially expressed genes (DEGs) for Taxane-Resistant AR^Low^/mCRPC/NEPC (PC-3TXR) *vs.* mCSPC/AR^low^ (PC-3) PCa cell lines.**

Gene expression was assessed by Next-generation RNA sequencing.

| **Gene Expression in RNA sequencing (mCRPC/NEPC/AR^Low^ vs. mCSPC/AR^High^) FDR<0.05.** | **Gene Expression in Single Cell RNA sequencing (mCRPC/NEPC/AR^Low^ vs. mCSPC/AR^High^) FDR<0.05.** | **Common DEGs among RNA sequencing and Single Cell RNA sequencing (mCRPC/NEPC/AR^Low^ vs. mCSPC/AR^High^) FDR<0.05.** |
| --- | --- | --- |
| PYGL | ZYX | ACSL4 |
| TBX3 | BCAT1 | AHNAK2 |
| ANXA2 | HAS3 | ANXA1 |
| FSCN1 | HMGA1 | ANXA2 |
| ITGB4 | IER3 | ARNTL2 |
| ANXA2P2 | IGF2BP2 | ASPH |
| JCAD | IL18 | CCDC88A |
| AHNAK2 | ASPH | CD109 |
| STEAP2 | ITGA3 | CD44 |
| CCDC88A | ITGA6 | CD59 |
| PAM | TMSB4X | CDKN2B |
| IDH1 | KLK4 | COTL1 |
| HID1 | GSTP1 | DENND3 |
| WWTR1 | KRT7 | DUSP5 |
| MYOF | AREG | EMP1 |
| HAS3 | AR | EPHA2 |
| SLC16A3 | APP | FMNL2 |
| ARNTL2 | LAMA4 | FRMD6 |
| RHOU | APOBEC3C | FSCN1 |
| MTCL1 | LGALS1 | GPAT3 |
| DUSP5 | LGALS3 | GPRC5A |
| SMAD3 | LMO7 | HAS3 |
| ARFGEF3 | LPAR1 | HMGA1 |
| PLCD3 | TMEFF2 | HTRA1 |
| PHLDA1 | KRT19 | IER3 |
| VIM | CD55 | ITGA3 |
| DHRS13 | GPRC5A | ITGB4 |
| IQANK1 | FXYD5 | JCAD |
| BDH1 | CD59 | KDELR3 |
| EPB41L2 | CD24 | KLHL5 |
| TGFA | CDA | LAMC2 |
| IER3 | COTL1 | LIF |
| PPP3CA | CRIP2 | MALL |
| TM7SF2 | CTSC | MAOA |
| CERK | UCHL1 | MET |
| GPRC5A | DCBLD2 | MSN |
| FLNA | DKK1 | MSRB3 |
| AJM1 | DMKN | MXRA7 |
| CREB3L4 | DNTTIP1 | MYOF |
| PLP2 | GPR153 | NKX3-1 |
| NTN4 | CAV1 | NT5E |
| MET | ETV4 | NTN4 |
| MYO6 | C19orf33 | OPN3 |
| MANSC1 | FCGR2A | PAM |
| KLHL5 | FHL2 | PHLDA1 |
| MITF | FOLH1 | PLAUR |
| MAOA | FOSL1 | PLCD3 |
| ZYX | FSCN1 | PLP2 |
| AFAP1 | FST | PLPP1 |
| ASPH | FSTL1 | PPL |
| CHST11 | VIM | PPP3CA |
| SARM1 | CAPN2 | PTPN14 |
| ACSL4 | MET | PYGL |
| ITGA3 | MIR4435-2HG | RHOF |
| HTRA1 | CD44 | RND3 |
| MXRA7 | MSMP | S100A6 |
| FHDC1 | ADGRF1 | SERPINE2 |
| RBM47 | PPP1R14B-AS1 | SLC16A3 |
| ADCY7 | PRDM8 | SPART |
| TRIP10 | PRSS3 | STEAP2 |
| EPHA2 | PYGL | TBX3 |
| S100A6 | RBP4 | TGFA |
| PRAF2 | RHOF | TGFBR2 |
| DYRK3 | S100A2 | TIMP2 |
| GPAT3 | S100A4 | TRIP10 |
| TGFBR2 | AGR2 | TSPAN5 |
| LIF | S100A6 | VIM |
| FMNL2 | SERPINE2 | WWTR1 |
| RHOF | SHISA3 | ZNF385B |
| LOXL2 | SULF2 | ZYX |
| ESRP1 | SLCO4A1 |  |
| DENND3 | SPTBN1 |  |
| EFHD2 | SSTR5-AS1 |  |
| CDKN2B | ABI3BP |  |
| PLAUR | ABCC3 |  |
| SH3KBP1 | S100A16 |  |
| ITGA2 | AHNAK2 |  |
| ZNF428 | PLP2 |  |
| MSN | ANTXR2 |  |
| MLKL | NR2F1 |  |
| PTK2B | ANXA1 |  |
| CAB39L | NES |  |
| PLPP1 | TG |  |
| MCTP1 | NRP1 |  |
| CD59 | NTN4 |  |
| EMP1 | WWTR1 |  |
| STEAP1 | NPAS2 |  |
| SIGIRR | NEFL |  |
| HMGA1 | PALM2-AKAP2 |  |
| SPART | MYOF |  |
| MSRB3 | TIMP2 |  |
| ZNF185 | MSN |  |
| PLEKHA2 | PHLDA1 |  |
| NKX3-1 | TM4SF1 |  |
| LAMC2 | PKP1 |  |
| STYK1 | TFF1 |  |
| POLR3G | ANXA2 |  |
| IPW | ALDH1A3 |  |
| TNFRSF12A | SPRY4 |  |
| SERPINE2 | TFCP2 |  |
| IKBIP | EDIL3 |  |
| CAPG | PLAT |  |
| CD109 | RBMS1 |  |
| ARG2 | PPP3CA |  |
| PTPN14 | FXYD3 |  |
| ANXA1 | HSD17B2 |  |
| RAB17 | PNMA1 |  |
| BMP6 | PLCXD3 |  |
| TMEM25 | MALL |  |
| PALM2AKAP2 | MIR99AHG |  |
| ZNF726 | RND3 |  |
| SBK1 | EPHA2 |  |
| FUT1 | PDLIM1 |  |
| NABP1 | ITGB4 |  |
| SEMA7A | PXDN |  |
| TTLL11 | PLCD3 |  |
| FRMD6 | DNAH5 |  |
| ERBB3 | SRGN |  |
| HEG1 | PPFIA2 |  |
| TP53INP1 | HMGA2 |  |
| NT5E | F2R |  |
| MALL | CHRM3 |  |
| CACFD1 | PTPN14 |  |
| OPN3 | PLAU |  |
| KDELR3 | AKAP12 |  |
| ZNF91 | EIF4EBP3 |  |
| ZNF385B | PLEKHH2 |  |
| PLA2G6 | HEY1 |  |
| RNF208 | CD70 |  |
| TSPAN5 | KRT75 |  |
| RND3 | NCAM2 |  |
| ELK3 | ITPRIPL2 |  |
| PPL | DPP4 |  |
| TIMP2 | TSPAN5 |  |
| CD44 | CCDC88A |  |
| FLJ20021 | SCRN1 |  |
| CCDC80 | TMEM156 |  |
| LINC00342 | AC005064.1 |  |
| CBS | GPRC5C |  |
| C1QL1 | CYTOR |  |
| COTL1 | RAB34 |  |
| HLA-A | SYNE4 |  |
| PGM2L1 | DOCK11 |  |
|  | BHLHE41 |  |
|  | PPARG |  |
|  | SYNJ2 |  |
|  | LINC00632 |  |
|  | IGFBP6 |  |
|  | CREB3L1 |  |
|  | ERRFI1 |  |
|  | AC010198.2 |  |
|  | TTN |  |
|  | PTPRB |  |
|  | SPON2 |  |
|  | BX640514.2 |  |
|  | NT5E |  |
|  | HPN |  |
|  | MDK |  |
|  | MXRA7 |  |
|  | MT1G |  |
|  | PRUNE2 |  |
|  | C5orf46 |  |
|  | SLC16A3 |  |
|  | DUSP5 |  |
|  | DUSP6 |  |
|  | SPTSSB |  |
|  | HSPG2 |  |
|  | FBN2 |  |
|  | PPL |  |
|  | COLCA1 |  |
|  | TENM4 |  |
|  | CDKN2B |  |
|  | CD68 |  |
|  | SMIM3 |  |
|  | UTS2B |  |
|  | DSC3 |  |
|  | NR3C1 |  |
|  | AKR1B1 |  |
|  | SEMA3D |  |
|  | UPP1 |  |
|  | OSBPL10 |  |
|  | ETV5 |  |
|  | INF2 |  |
|  | BHLHE40 |  |
|  | CMTM7 |  |
|  | FUT11 |  |
|  | KCNN4 |  |
|  | NKX3-1 |  |
|  | KLHL5 |  |
|  | SYNE1 |  |
|  | GUCY1A2 |  |
|  | PGM5 |  |
|  | NNMT |  |
|  | SOWAHC |  |
|  | RDH10 |  |
|  | LOXL1-AS1 |  |
|  | MAOA |  |
|  | DBNDD2 |  |
|  | PAM |  |
|  | APCDD1L-DT |  |
|  | FMNL2 |  |
|  | SERPINB5 |  |
|  | SP8 |  |
|  | LRMDA |  |
|  | AC015712.1 |  |
|  | PLIN2 |  |
|  | SEMA6A |  |
|  | PPP1R18 |  |
|  | ANKRD29 |  |
|  | SNHG18 |  |
|  | SYT1 |  |
|  | PHF19 |  |
|  | HHEX |  |
|  | PROCR |  |
|  | RAB31 |  |
|  | TGFBR2 |  |
|  | PTGES |  |
|  | COL6A2 |  |
|  | C17orf67 |  |
|  | AC093673.1 |  |
|  | MB |  |
|  | FCHO1 |  |
|  | EMP3 |  |
|  | TRIM58 |  |
|  | ANXA3 |  |
|  | RAC2 |  |
|  | STEAP2 |  |
|  | EFNB1 |  |
|  | KRT81 |  |
|  | TBX3 |  |
|  | TNS3 |  |
|  | CTHRC1 |  |
|  | IGFBP4 |  |
|  | TOX2 |  |
|  | CRABP2 |  |
|  | CD109 |  |
|  | PMP22 |  |
|  | KCTD12 |  |
|  | ARHGAP6 |  |
|  | CLMP |  |
|  | OSMR |  |
|  | MAML2 |  |
|  | PHLDB2 |  |
|  | CAV2 |  |
|  | MST1R |  |
|  | LTBP1 |  |
|  | EMP1 |  |
|  | FHL1 |  |
|  | ZBTB16 |  |
|  | B3GNT3 |  |
|  | FRMD6 |  |
|  | SH3TC2-DT |  |
|  | HSPB6 |  |
|  | HTRA1 |  |
|  | PLPP3 |  |
|  | SELENOP |  |
|  | NAV2 |  |
|  | GALNT5 |  |
|  | WFDC2 |  |
|  | NAALADL2 |  |
|  | CCN3 |  |
|  | GUCY1A1 |  |
|  | LAMC2 |  |
|  | INHBB |  |
|  | RUNX1 |  |
|  | TRGC1 |  |
|  | TMEM255B |  |
|  | EPHA3 |  |
|  | AVPI1 |  |
|  | ICAM1 |  |
|  | B3GNT5 |  |
|  | CCN1 |  |
|  | AC020916.1 |  |
|  | MMP13 |  |
|  | ANXA10 |  |
|  | CD27-AS1 |  |
|  | AP001816.1 |  |
|  | BCAR3 |  |
|  | MME |  |
|  | LUZP2 |  |
|  | JAG1 |  |
|  | LAMB3 |  |
|  | AMOTL2 |  |
|  | PAPSS2 |  |
|  | MUC1 |  |
|  | LY6K |  |
|  | AC027031.2 |  |
|  | DAB2 |  |
|  | CYP1B1 |  |
|  | G0S2 |  |
|  | RAB25 |  |
|  | LMO3 |  |
|  | MYEOV |  |
|  | SCAT8 |  |
|  | OBSL1 |  |
|  | ARNTL2 |  |
|  | GJB2 |  |
|  | DENND3 |  |
|  | AC083855.2 |  |
|  | SIDT1 |  |
|  | KDELR3 |  |
|  | STEAP3 |  |
|  | GEM |  |
|  | LINC02197 |  |
|  | CNRIP1 |  |
|  | IFI27 |  |
|  | RTL8B |  |
|  | SAMD4A |  |
|  | MUC5B |  |
|  | AXL |  |
|  | ACSL4 |  |
|  | CAVIN1 |  |
|  | IFITM1 |  |
|  | GPAT3 |  |
|  | KCNK15 |  |
|  | VEGFC |  |
|  | SH3TC2 |  |
|  | PRICKLE1 |  |
|  | TGFB2 |  |
|  | FAM133A |  |
|  | AL589182.1 |  |
|  | HOXA13 |  |
|  | LNP1 |  |
|  | LCN2 |  |
|  | PKIA |  |
|  | MUC3A |  |
|  | PLD1 |  |
|  | HGF |  |
|  | LURAP1L |  |
|  | PLPP1 |  |
|  | EHF |  |
|  | C1QTNF1 |  |
|  | PSMB8 |  |
|  | LIF |  |
|  | DLGAP1-AS2 |  |
|  | JCAD |  |
|  | ARSJ |  |
|  | WDR72 |  |
|  | FAM131B |  |
|  | CCDC144NL-AS1 |  |
|  | REG4 |  |
|  | ARHGAP29-AS1 |  |
|  | OPN3 |  |
|  | LIN7A |  |
|  | CLIP4 |  |
|  | EREG |  |
|  | PRKACB |  |
|  | KIFC3 |  |
|  | AC016831.1 |  |
|  | GRAMD1B |  |
|  | TGFA |  |
|  | WFDC3 |  |
|  | MISP |  |
|  | PART1 |  |
|  | PLCE1 |  |
|  | ALDH3B1 |  |
|  | AC010327.4 |  |
|  | TRIB2 |  |
|  | FAM83A |  |
|  | NCEH1 |  |
|  | LINC00920 |  |
|  | RNF182 |  |
|  | TRIM9 |  |
|  | AFF3 |  |
|  | PLAUR |  |
|  | IFI16 |  |
|  | FAM111B |  |
|  | MAP1LC3A |  |
|  | APLN |  |
|  | MSRB3 |  |
|  | UBE2L6 |  |
|  | CEMIP |  |
|  | ST3GAL6 |  |
|  | AL365181.3 |  |
|  | PGAM2 |  |
|  | CT83 |  |
|  | SEMA4A |  |
|  | STXBP5L |  |
|  | AL354707.1 |  |
|  | SLIT2 |  |
|  | MMEL1 |  |
|  | ZEB1 |  |
|  | CPM |  |
|  | TRIP10 |  |
|  | KLHDC9 |  |
|  | AC104667.2 |  |
|  | ALOX15 |  |
|  | GRHL2 |  |
|  | VSTM5 |  |
|  | AIFM2 |  |
|  | CLCF1 |  |
|  | MYL9 |  |
|  | C15orf48 |  |
|  | SLC30A4 |  |
|  | KIF1A |  |
|  | PROM2 |  |
|  | CSAG3 |  |
|  | CXCL2 |  |
|  | IGFBP5 |  |
|  | TACSTD2 |  |
|  | NRN1 |  |
|  | LINC01515 |  |
|  | PTPRH |  |
|  | CCDC28B |  |
|  | SNTB1 |  |
|  | PLEK2 |  |
|  | CACNA1A |  |
|  | KRT80 |  |
|  | NPY1R |  |
|  | TC2N |  |
|  | SPART |  |
|  | LCP1 |  |
|  | ARL4C |  |
|  | MMP1 |  |
|  | AC127526.5 |  |
|  | TMEM125 |  |
|  | EPAS1 |  |
|  | APOL6 |  |
|  | TGM2 |  |
|  | KYNU |  |
|  | TNXB |  |
|  | LUCAT1 |  |
|  | ZNF736 |  |
|  | FGF2 |  |
|  | DPF3 |  |
|  | TMC5 |  |
|  | SFTA3 |  |
|  | CFAP69 |  |
|  | METTL7A |  |
|  | NMB |  |
|  | MIR31HG |  |
|  | SLC26A5 |  |
|  | TIAM1 |  |
|  | SCARA5 |  |
|  | OAS2 |  |
|  | ZNF385B |  |
|  | AL161431.1 |  |
|  | ITGA1 |  |
|  | GPM6A |  |
|  | AP003900.1 |  |
|  | C1orf21 |  |
|  | ACOX2 |  |
|  | MEIS2 |  |
|  | SERPINA1 |  |
|  | CLDN1 |  |
|  | AKT3 |  |
|  | SPINK1 |  |
|  | TOX3 |  |
|  | KIAA1324 |  |
|  | NFATC2 |  |
|  | SIRPA |  |
|  | BX284668.5 |  |
|  | STRIP2 |  |
|  | MB21D2 |  |
|  | HSD17B8 |  |
|  | NXN |  |
|  | LINC00513 |  |
|  | FLNC |  |
|  | PON1 |  |
|  | PLAG1 |  |
|  | LAMA5-AS1 |  |
|  | MX1 |  |
|  | TFPI2 |  |
|  | LINC02780 |  |
|  | HIF1A-AS3 |  |
|  | TNS4 |  |
|  | SLCO1B3 |  |
|  | KITLG |  |
|  | SYCE3 |  |
|  | ZNF655 |  |
|  | AZGP1 |  |
|  | PGM5-AS1 |  |
|  | MMP14 |  |
|  | PLSCR4 |  |
|  | AP001636.3 |  |
|  | NRXN1 |  |
|  | LINC02577 |  |
|  | PRSS8 |  |
|  | CASZ1 |  |
|  | KCNQ3 |  |
|  | ARSD |  |
|  | UGT2B15 |  |
|  | SEMA3A |  |
|  | HERC3 |  |
|  | NETO1 |  |
|  | AP003498.2 |  |
|  | ETS1 |  |
|  | RAB38 |  |
|  | CBLN2 |  |
|  | TMEM173 |  |
|  | AP000527.1 |  |
|  | RORC |  |
|  | HMGCS2 |  |
|  | ITGA9-AS1 |  |
|  | RNF212 |  |
|  | RSPO3 |  |
|  | TPTE |  |
|  | AP004609.3 |  |
|  | ZNF385D |  |
|  | SFTA1P |  |
|  | GASK1B |  |
|  | KLK1 |  |
|  | ADA2 |  |
|  | SPARC |  |
|  | IL27RA |  |
|  | IFI44L |  |
|  | POPDC3 |  |
|  | PCDH11Y |  |
|  | MT1M |  |
|  | TNFAIP2 |  |
|  | VCX3B |  |
|  | DRAIC |  |
|  | GPR158 |  |
|  | SERPIND1 |  |
|  | IGFBP2 |  |
|  | GAS2 |  |
|  | LINC02055 |  |
|  | ZNF737 |  |
|  | AL024508.2 |  |
|  | PARP6 |  |
|  | RIMS1 |  |
|  | ZNF682 |  |
|  | EDA |  |
|  | GPT |  |
|  | VIL1 |  |
|  | DNASE2B |  |
|  | AC138761.1 |  |
|  | COCH |  |
|  | MAPK8IP2 |  |
|  | EPPK1 |  |
|  | S100A14 |  |
|  | TMEM132D-AS1 |  |
|  | GPX3 |  |
|  | UBASH3B |  |
|  | BTBD11 |  |
|  | NEXN |  |
|  | FAM110B |  |
|  | NDRG2 |  |
|  | PRR15L |  |
|  | SLITRK6 |  |
|  | IL1RL1 |  |
|  | SLC44A4 |  |
|  | SERTAD4-AS1 |  |
|  | AC034213.1 |  |
|  | GPX2 |  |
|  | AC079062.1 |  |
|  | SCEL |  |
|  | GRIN3A |  |
|  | MIR4458HG |  |
|  | CCDC74A |  |
|  | CGB2 |  |
|  | SYNPO |  |
|  | S100A9 |  |
|  | LINP1 |  |
|  | HOXC6 |  |
|  | NOX5 |  |
|  | SV2A |  |
|  | VSIR |  |
|  | CD74 |  |
|  | TFF3 |  |
|  | UNC13A |  |
|  | TMEM215 |  |
|  | LINC01213 |  |
|  | PLXDC2 |  |
|  | LINC01980 |  |
|  | COL9A3 |  |
|  | NLGN1 |  |
|  | MAGEA2 |  |
|  | LINC01666 |  |
|  | PLA2G4F |  |
|  | VGF |  |
|  | HBQ1 |  |
|  | CXCL8 |  |
|  | BMP4 |  |
|  | AP000842.3 |  |
|  | ACPP |  |
|  | NAPSA |  |
|  | LINC01836 |  |
|  | FBP1 |  |
|  | ZNF727 |  |
|  | POTEF |  |
|  | TNFAIP3 |  |
|  | FAM198B-AS1 |  |
|  | CRABP1 |  |
|  | RINL |  |
|  | LINC01234 |  |
|  | AC239800.2 |  |
|  | SCHLAP1 |  |
|  | NOSTRIN |  |
|  | RLN1 |  |
|  | ZIC1 |  |
|  | AC010789.1 |  |
|  | STEAP4 |  |
|  | GALNT8 |  |
|  | DOCK10 |  |
|  | TGFBI |  |
|  | CD40 |  |
|  | AC113383.1 |  |
|  | GADD45G |  |
|  | NOS3 |  |
|  | LRRN1 |  |
|  | PRLR |  |
|  | EHD2 |  |
|  | ACKR3 |  |
|  | ALDH3B2 |  |
|  | SCG3 |  |
|  | HGD |  |
|  | LINC01146 |  |
|  | CSF1 |  |
|  | AL135910.1 |  |
|  | IRX3 |  |
|  | BST2 |  |
|  | KIF12 |  |
|  | SH3RF2 |  |
|  | MYBPC1 |  |
|  | ZMYND12 |  |
|  | NEFH |  |
|  | SCUBE2 |  |
|  | DOCK8 |  |
|  | MUC16 |  |
|  | PTP4A1 |  |
|  | SHD |  |
|  | ALB |  |
|  | COLEC12 |  |
|  | EFEMP1 |  |
|  | CACNB2 |  |
|  | TFF2 |  |
|  | CELF2 |  |
|  | AC005920.1 |  |
|  | TSPEAR-AS2 |  |
|  | LINC01687 |  |
|  | LINC02057 |  |
|  | SFTA2 |  |
|  | SPOCD1 |  |
|  | EPHA7 |  |
|  | DDC |  |
|  | POTEI |  |
|  | DERL3 |  |
|  | SLAMF7 |  |
|  | BX004987.1 |  |
|  | ABCC2 |  |
|  | CPLX2 |  |
|  | ID4 |  |
|  | P2RY6 |  |
|  | NR4A3 |  |
|  | LGSN |  |
|  | SMOC1 |  |
|  | PEG3 |  |
|  | AL022068.1 |  |
|  | DNAH6 |  |
|  | NPY |  |
|  | MSMB |  |

**Supplementary Table 7. Common and Unique differentially expressed genes (DEGs) among RNA sequencing and single cell RNA sequencing for all PCa cell lines.**

Gene expression was assessed by Next-generation RNA sequencing in all PCa cell lines. Fold change cut-off value for RNAseq was > 2 and Single-cell RNA sequencing using the droplet sequencing method (10X Genomics) was performed on all PCa cell lines. Each dot represents a single cell. Contaminated (doublet) cells were not included.
