# Supplementary material for: Metronomic Administration of Topotecan Alone and in Combination with Docetaxel Inhibits Epithelial–mesenchymal Transition in Aggressive Variant Prostate Cancers
Source: Cancer Res Commun. 2023 Jul 19;3(7):1286–311. doi: 10.1158/2767-9764.CRC-22-0427 (PMC10355222; doi:10.1158/2767-9764.CRC-22-0427)
Supplement: Supplementary Figure 1 — Supplementary Fig. 1 shows Differentially expressed gene signature (DEGs) based on next gene sequencing (mRNA sequencing) for ARLow /mCRPC/NEPC (PC-3, PC-3M, DU145) vs ARHigh /mCSPC (LNCaP, VCAP, 22RV1) prostate cancer (PCa) cell lines and normal prostate cell lines RWPE1 and RWPE2. Volcano plots representing mRNA expression for all cell lines. (blue p<0.05 and gray>0.05) HeatMap representing Differentially Expressed Gene Signature (DEGs) for ARLow/mCRPC/NEPC (PC-3, PC-3M, DU145) vs ARHigh/mCSPC (LNCaP, VCAP, 22RV1) PCa and normal prostate cell lines RWPE1 and RWPE2 (FDR<0.05 and TOP-100). [file crc-22-0427-s03.pptx]

## Slide 1
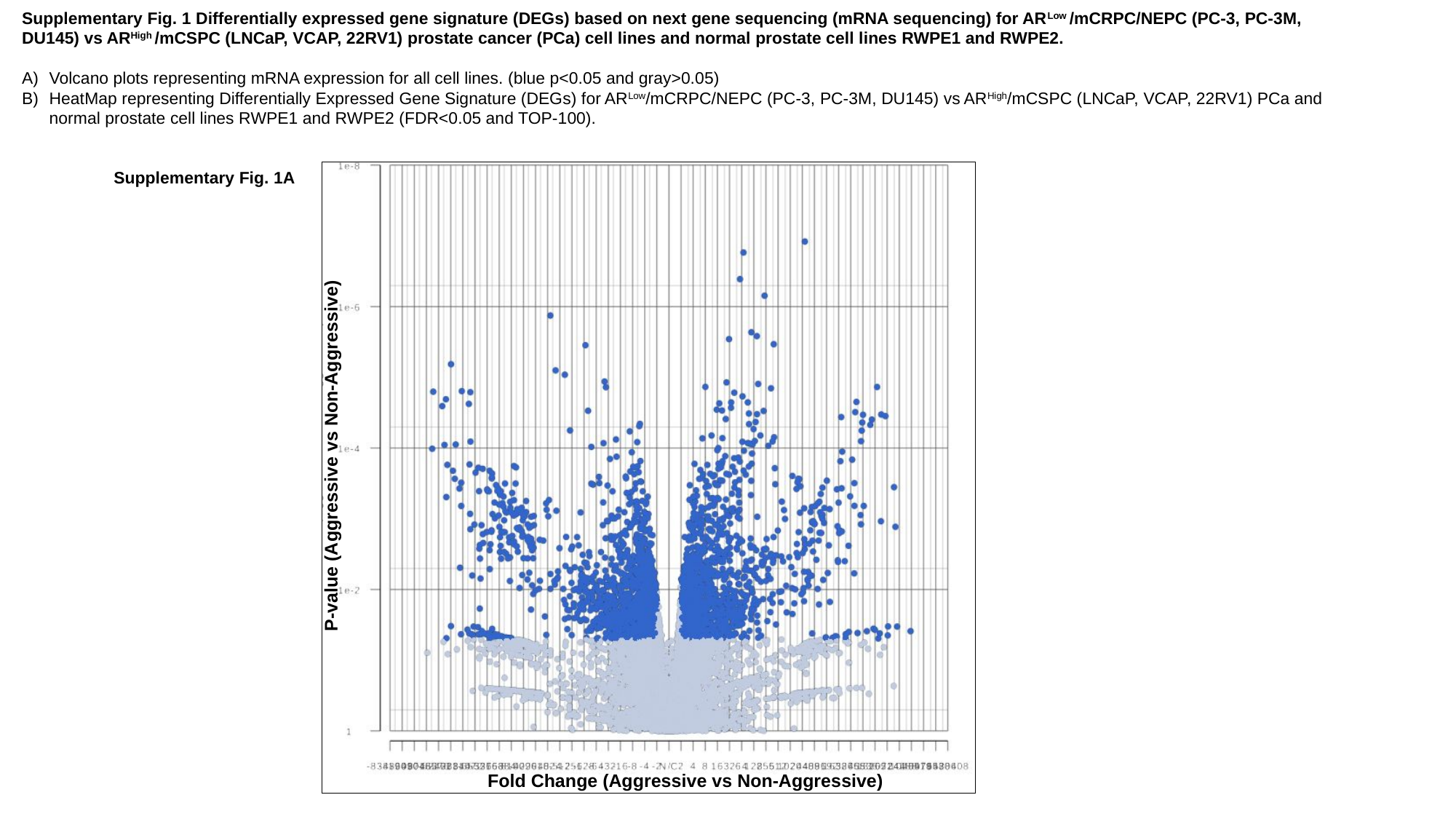

Supplementary Fig. 1 Differentially expressed gene signature (DEGs) based on next gene sequencing (mRNA sequencing) for ARLow /mCRPC/NEPC (PC-3, PC-3M, DU145) vs ARHigh /mCSPC (LNCaP, VCAP, 22RV1) prostate cancer (PCa) cell lines and normal prostate cell lines RWPE1 and RWPE2.
Volcano plots representing mRNA expression for all cell lines. (blue p<0.05 and gray>0.05)
HeatMap representing Differentially Expressed Gene Signature (DEGs) for ARLow/mCRPC/NEPC (PC-3, PC-3M, DU145) vs ARHigh/mCSPC (LNCaP, VCAP, 22RV1) PCa and normal prostate cell lines RWPE1 and RWPE2 (FDR<0.05 and TOP-100).
Supplementary Fig. 1A
P-value (Aggressive vs Non-Aggressive)
Fold Change (Aggressive vs Non-Aggressive)

## Slide 2
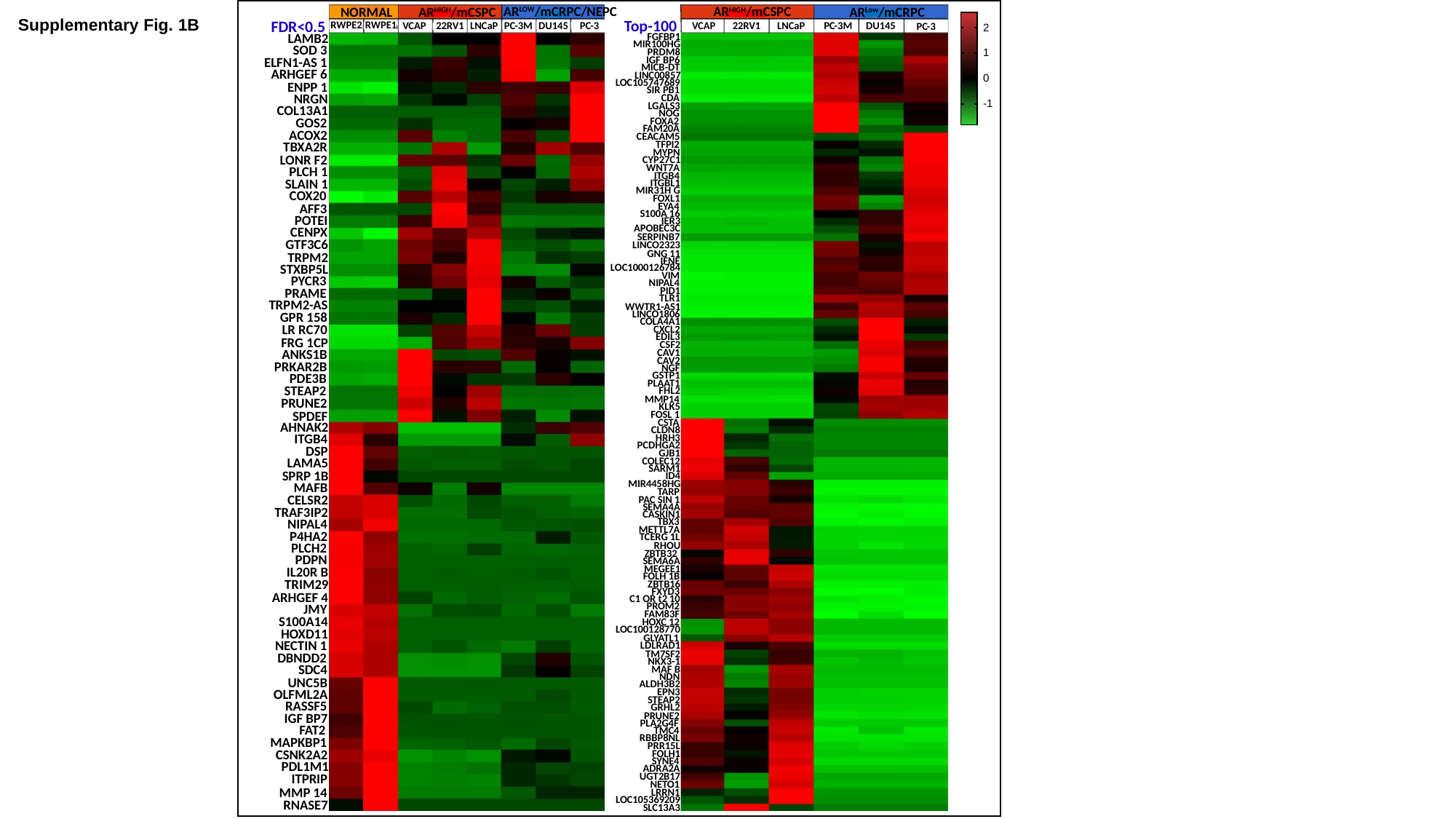

ARLOW/mCRPC/NEPC
ARHIGH/mCSPC
NORMAL
RWPE2
RWPE1
DU145
PC-3M
22RV1
PC-3
VCAP
LNCaP
LAMB2
SOD 3
ELFN1-AS 1
ARHGEF 6
ENPP 1
NRGN
COL13A1
GOS2
ACOX2
TBXA2R
LONR F2
PLCH 1
SLAIN 1
COX20
AFF3
POTEI
CENPX
GTF3C6
TRPM2
STXBP5L
PYCR3
PRAME
TRPM2-AS
GPR 158
LR RC70
FRG 1CP
ANKS1B
PRKAR2B
PDE3B
STEAP2
PRUNE2
SPDEF
AHNAK2
ITGB4
DSP
LAMA5
SPRP 1B
MAFB
CELSR2
TRAF3IP2
NIPAL4
P4HA2
PLCH2
PDPN
IL20R B
TRIM29
ARHGEF 4
JMY
S100A14
HOXD11
NECTIN 1
DBNDD2
SDC4
UNC5B
OLFML2A
RASSF5
IGF BP7
FAT2
MAPKBP1
CSNK2A2
PDL1M1
ITPRIP
MMP 14
RNASE7
ARHIGH/mCSPC
ARLow/mCRPC
DU145
PC-3M
LNCaP
22RV1
VCAP
PC-3
FGFBP1
MIR100HG
PRDM8
IGF BP6
MICB-DT
LINC00857
LOC105747689
SIR PB1
CDA
LGALS3
NOG
FOXA2
FAM20A
CEACAM5
TFPI2
MYPN
CYP27C1
WNT7A
ITGB4
ITGBL1
MIR31H G
FOXL1
EYA4
S100A 16
IER3
APOBEC3C
SERPINB7
LINCO2323
GNG 11
IFNE
LOC1000126784
VIM
NIPAL4
PID1
TLR1
WWTR1-AS1
LINCO1806
COLA4A1
CXCL2
EDIL3
CSF2
CAV1
CAV2
NGF
GSTP1
PLAAT1
FHL2
MMP14
KLK5
FOSL 1
CSTA
CLDN8
HRH3
PCDHGA2
GJB1
COLEC12
SARM1
ID4
MIR4458HG
TARP
PAC SIN 1
SEMA4A
CASKIN1
TBX3
METTL7A
TCERG 1L
RHOU
ZBTB32
SEMA6A
MEGEE1
FOLH 1B
ZBTB16
FXYD3
C1 OR t2 10
PROM2
FAM83F
HOXC 12
LOC100128770
GLYATL1
LDLRAD1
TM7SF2
NKX3-1
MAF B
NDN
ALDH3B2
EPN3
STEAP2
GRHL2
PRUNE2
PLA2G4F
TMC4
RBBP8NL
PRR15L
FOLH1
SYNE4
ADRA2A
UGT2B17
NETO1
LRRN1
LOC105369209
SLC13A3
Supplementary Fig. 1B
Top-100
FDR<0.5
