# Supplementary material for: Metronomic Administration of Topotecan Alone and in Combination with Docetaxel Inhibits Epithelial–mesenchymal Transition in Aggressive Variant Prostate Cancers
Source: Cancer Res Commun. 2023 Jul 19;3(7):1286–311. doi: 10.1158/2767-9764.CRC-22-0427 (PMC10355222; doi:10.1158/2767-9764.CRC-22-0427)
Supplement: Supplementary Figure 2 — Supplementary Fig. 2 shows Ingenuity pathway analysis (IPA) predictions for European American/ Caucasian American (EA/CA) ARHigh/mCSPC (LNCaP, VCAP, 22RV1), ARLow/mCRPC/NEPC (PC-3, PC-3M, DU145) and African American (AA) ARHigh/mCSPC (MDA-Pca-2b) PCa cell lines as well as normal prostate cell lines RWPE1 and RWPE2. IPA predicted A) Diseases and biological pathways for development of ARLow/mCRPC/NEPC. Major pathways are cell movement, morbidity, mortality, migration, invasion, survival, viability and development of vasculature- vasculogenesis and angiogenesis. B) Causal network pathway for development of ARLow/mCRPC/NEPC, which include oxidative phosphorylation, p70S6K, unfolded response, TREM1 signaling, NF-kB signaling, ERK5 signaling, IL-8 signaling, BAG2 signaling, Integrin signaling and VEGF signaling. C) IPA predicted HIF1α and EMT as key pathways associated with PCa development in African Americans (AA). [file crc-22-0427-s04.pptx]

## Slide 1
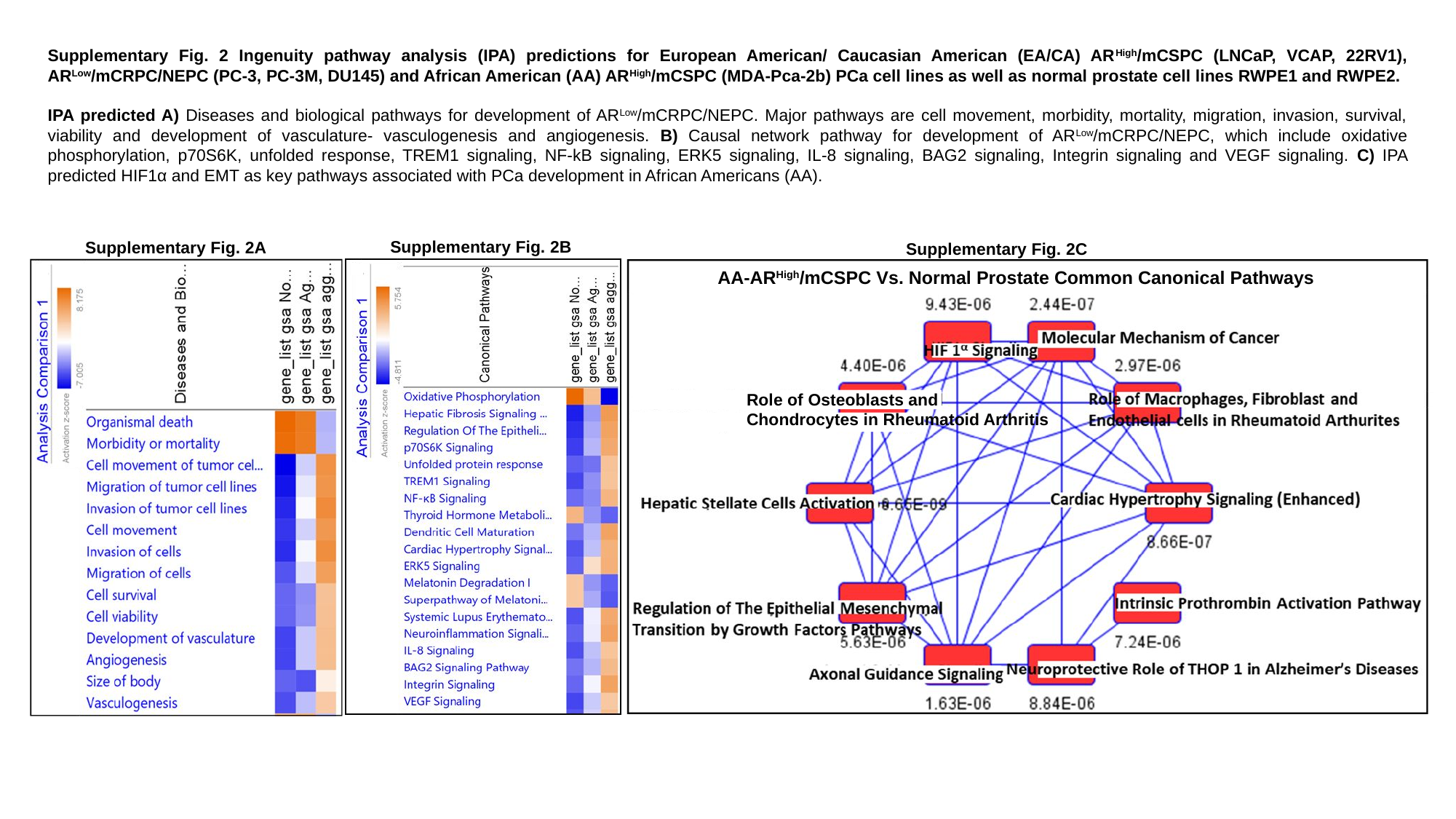

Supplementary Fig. 2 Ingenuity pathway analysis (IPA) predictions for European American/ Caucasian American (EA/CA) ARHigh/mCSPC (LNCaP, VCAP, 22RV1), ARLow/mCRPC/NEPC (PC-3, PC-3M, DU145) and African American (AA) ARHigh/mCSPC (MDA-Pca-2b) PCa cell lines as well as normal prostate cell lines RWPE1 and RWPE2.
IPA predicted A) Diseases and biological pathways for development of ARLow/mCRPC/NEPC. Major pathways are cell movement, morbidity, mortality, migration, invasion, survival, viability and development of vasculature- vasculogenesis and angiogenesis. B) Causal network pathway for development of ARLow/mCRPC/NEPC, which include oxidative phosphorylation, p70S6K, unfolded response, TREM1 signaling, NF-kB signaling, ERK5 signaling, IL-8 signaling, BAG2 signaling, Integrin signaling and VEGF signaling. C) IPA predicted HIF1α and EMT as key pathways associated with PCa development in African Americans (AA).
Supplementary Fig. 2B
Supplementary Fig. 2A
Supplementary Fig. 2C
AA-ARHigh/mCSPC Vs. Normal Prostate Common Canonical Pathways
Role of Osteoblasts and Chondrocytes in Rheumatoid Arthritis
