# Supplementary material for: Metronomic Administration of Topotecan Alone and in Combination with Docetaxel Inhibits Epithelial–mesenchymal Transition in Aggressive Variant Prostate Cancers
Source: Cancer Res Commun. 2023 Jul 19;3(7):1286–311. doi: 10.1158/2767-9764.CRC-22-0427 (PMC10355222; doi:10.1158/2767-9764.CRC-22-0427)
Supplement: Supplementary Figure 3 — Supplementary Fig. 3 shows Differentially Expressed Gene Signatures (DEGs) based on next-gene sequencing (mRNA sequencing) for ARLow/mCRPC/NEPC (PC-3DU145), ARLow/mCSPC/NEPC taxane resistant (DUTXR and PC-3TXR) PCa cell lines. IPA predicted key pathways based on DEGs between Taxane resistant and sensitivity. Fig. 3A. Differentially expressed gene Signature was identified among DEGs for ARLow/mCRPC/NEPC (PC-3DU145), ARLow/mCSPC/NEPC taxane-resistant (DUTXR and PC-3TXR) PCa cell lines. Venn diagrams represent unique and common DEGs for ARLow/mCRPC/NEPC (PC-3, DU145), and ARLow/mCSPC/NEPCtaxane-resistant (DUTXR and PC-3TXR) cell lines. Fig. 3B. IPA predicted key pathways based on DEGs between Taxane resistant and sensitivity. Cluster 2 (IPA predicted activation of PPARA), Cluster 4 (ERK/MAPK signaling, MSP-RON Signaling, upregulation of FOXM1, ESR1, CEBPB, XBP1, and NPM1), Cluster 6 (ATM signaling, and MYC). [file crc-22-0427-s05.pptx]

## Slide 1
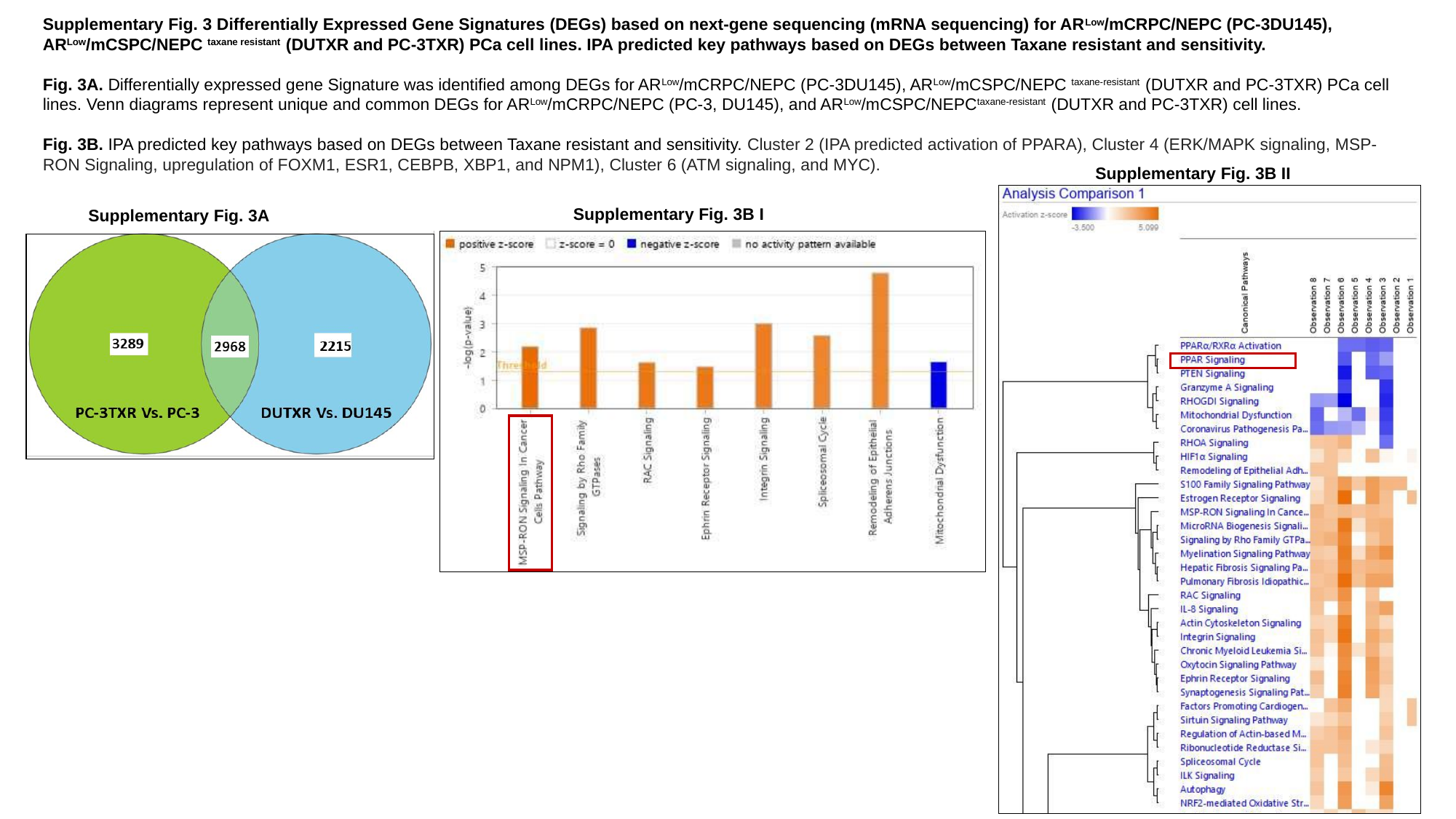

Supplementary Fig. 3 Differentially Expressed Gene Signatures (DEGs) based on next-gene sequencing (mRNA sequencing) for ARLow/mCRPC/NEPC (PC-3DU145), ARLow/mCSPC/NEPC taxane resistant (DUTXR and PC-3TXR) PCa cell lines. IPA predicted key pathways based on DEGs between Taxane resistant and sensitivity.
Fig. 3A. Differentially expressed gene Signature was identified among DEGs for ARLow/mCRPC/NEPC (PC-3DU145), ARLow/mCSPC/NEPC taxane-resistant (DUTXR and PC-3TXR) PCa cell lines. Venn diagrams represent unique and common DEGs for ARLow/mCRPC/NEPC (PC-3, DU145), and ARLow/mCSPC/NEPCtaxane-resistant (DUTXR and PC-3TXR) cell lines.
Fig. 3B. IPA predicted key pathways based on DEGs between Taxane resistant and sensitivity. Cluster 2 (IPA predicted activation of PPARA), Cluster 4 (ERK/MAPK signaling, MSP-RON Signaling, upregulation of FOXM1, ESR1, CEBPB, XBP1, and NPM1), Cluster 6 (ATM signaling, and MYC).
Supplementary Fig. 3B II
Supplementary Fig. 3B I
Supplementary Fig. 3A
