# Supplementary material for: Metronomic Administration of Topotecan Alone and in Combination with Docetaxel Inhibits Epithelial–mesenchymal Transition in Aggressive Variant Prostate Cancers
Source: Cancer Res Commun. 2023 Jul 19;3(7):1286–311. doi: 10.1158/2767-9764.CRC-22-0427 (PMC10355222; doi:10.1158/2767-9764.CRC-22-0427)
Supplement: Supplementary Figure 4 — Supplementary Fig. 4 shows Differentially Expressed Gene Signature (DEGs) based on next-gene sequencing (mRNA sequencing) and single cell RNA sequencing for all PCa cell lines. Differentially expressed gene Signature was identified among DEGs for RNS seq and scRNAseq. [file crc-22-0427-s06.pptx]

## Slide 1
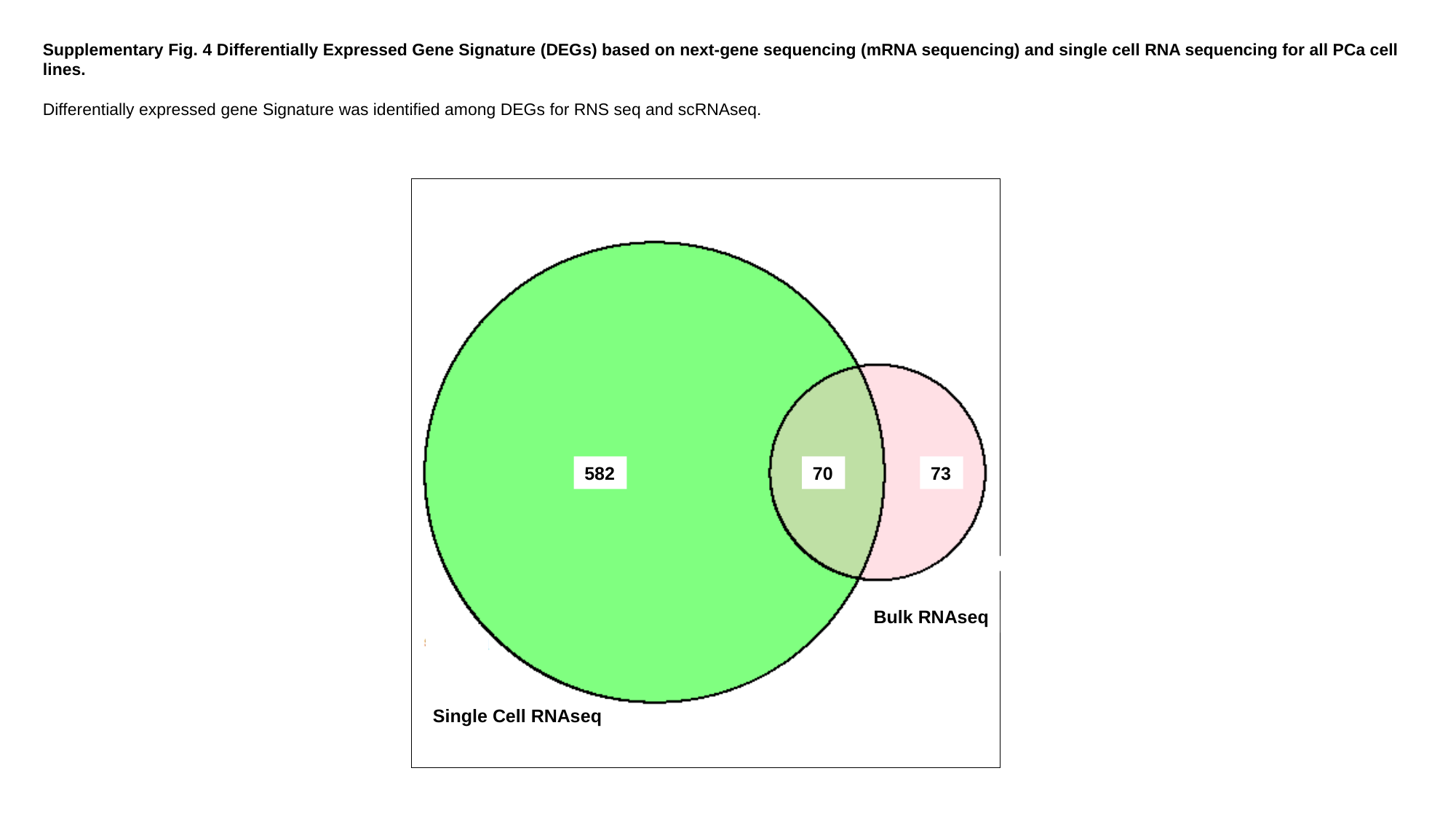

Supplementary Fig. 4 Differentially Expressed Gene Signature (DEGs) based on next-gene sequencing (mRNA sequencing) and single cell RNA sequencing for all PCa cell lines.
Differentially expressed gene Signature was identified among DEGs for RNS seq and scRNAseq.
582
70
73
Bulk RNAseq
Single Cell RNAseq
