# Supplementary material for: Metronomic Administration of Topotecan Alone and in Combination with Docetaxel Inhibits Epithelial–mesenchymal Transition in Aggressive Variant Prostate Cancers
Source: Cancer Res Commun. 2023 Jul 19;3(7):1286–311. doi: 10.1158/2767-9764.CRC-22-0427 (PMC10355222; doi:10.1158/2767-9764.CRC-22-0427)
Supplement: Supplementary Figure 5 — Supplementary Fig. 5 shows Ingenuity pathway analysis (IPA) predictions for common DEGs among RNAseq vs. scRNAseq in all PCa cell lines IPA predicted A) Diseases and biological pathways for common DGEs. Major pathways are cell invasion, movement, neoplasia, migration, transformation, metastatic and growth B) Causal network pathway for common DEGS include LY2109761 (TGF-β receptor inhibitor), IGF2R (receptor for both insulin-like growth factor 2), IL11RA (cytokine), SEMG2 (semenogelin proteins), CCR2 (chemokine receptor type 2), TRAF3IP3 (mediates cell growth by modulating the c-Jun N-terminal kinase signal transduction pathway), GGTI-2154 (inhibitor of geranylgeranyltransferase I ), L778123 (inhibitor of FPTase and GGPTase-I), TGFB1 and CXCL5. C) upstream regulators for common DEGs were THBF1, RAF1, AGT, ER receptor, OSM, SLC15A4, JUN, and IL1B D) Canonical pathways for common DEGs were s100 family, Hepatic fibrosis, Rho family, RHOGDI and integrin singling pathway. [file crc-22-0427-s07.pptx]

## Slide 1
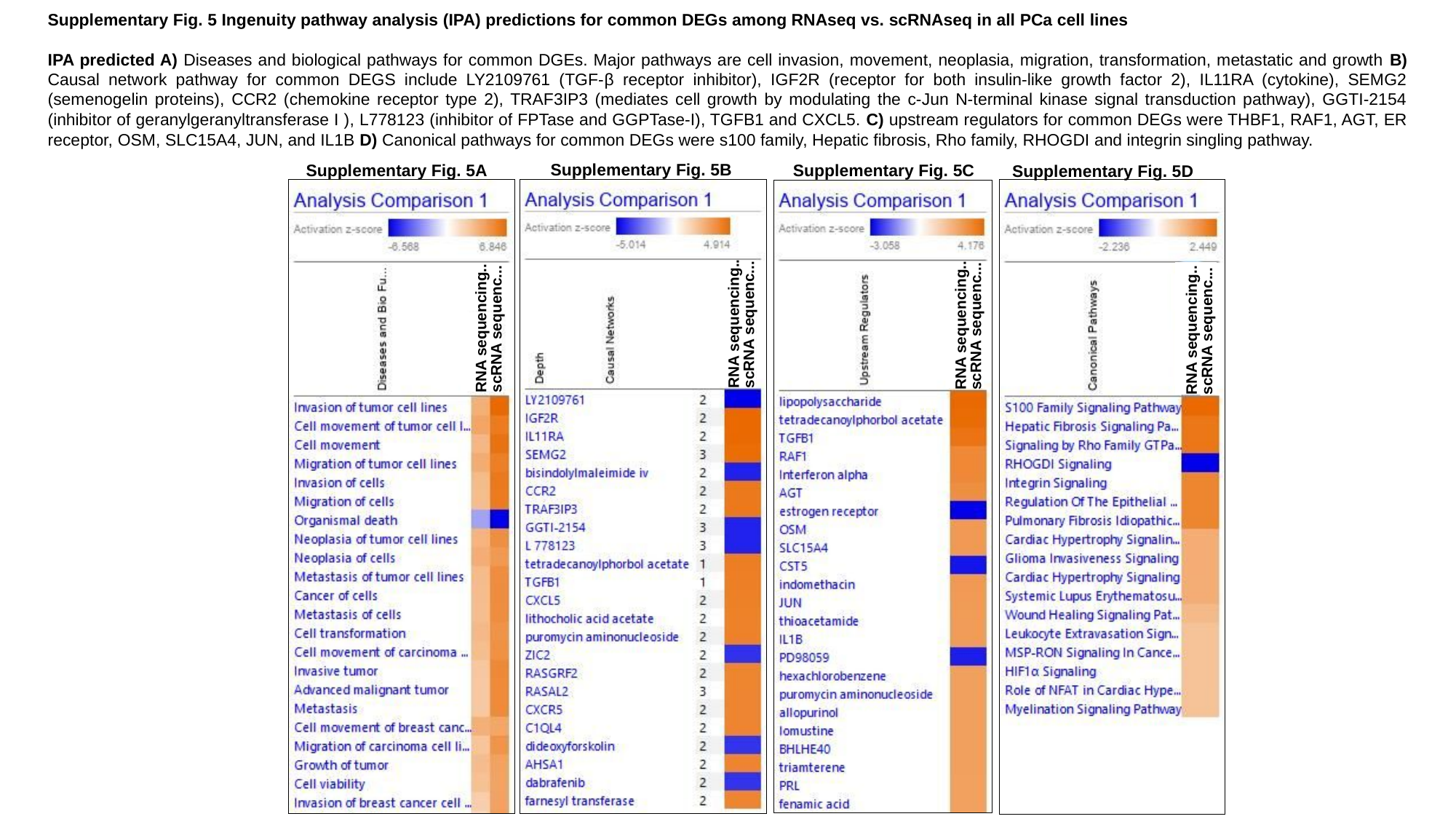

Supplementary Fig. 5 Ingenuity pathway analysis (IPA) predictions for common DEGs among RNAseq vs. scRNAseq in all PCa cell lines
IPA predicted A) Diseases and biological pathways for common DGEs. Major pathways are cell invasion, movement, neoplasia, migration, transformation, metastatic and growth B) Causal network pathway for common DEGS include LY2109761 (TGF-β receptor inhibitor), IGF2R (receptor for both insulin-like growth factor 2), IL11RA (cytokine), SEMG2 (semenogelin proteins), CCR2 (chemokine receptor type 2), TRAF3IP3 (mediates cell growth by modulating the c-Jun N-terminal kinase signal transduction pathway), GGTI-2154 (inhibitor of geranylgeranyltransferase I ), L778123 (inhibitor of FPTase and GGPTase-I), TGFB1 and CXCL5. C) upstream regulators for common DEGs were THBF1, RAF1, AGT, ER receptor, OSM, SLC15A4, JUN, and IL1B D) Canonical pathways for common DEGs were s100 family, Hepatic fibrosis, Rho family, RHOGDI and integrin singling pathway.
Supplementary Fig. 5B
Supplementary Fig. 5A
Supplementary Fig. 5C
Supplementary Fig. 5D
RNA sequencing..
scRNA sequenc...
RNA sequencing..
scRNA sequenc...
RNA sequencing..
scRNA sequenc...
RNA sequencing..
scRNA sequenc...
