# Supplementary material for: Metronomic Administration of Topotecan Alone and in Combination with Docetaxel Inhibits Epithelial–mesenchymal Transition in Aggressive Variant Prostate Cancers
Source: Cancer Res Commun. 2023 Jul 19;3(7):1286–311. doi: 10.1158/2767-9764.CRC-22-0427 (PMC10355222; doi:10.1158/2767-9764.CRC-22-0427)
Supplement: Supplementary Figure 6 — Supplementary Fig. 6 shows Cytotoxicity Profiling of ARLow/mCRPC/NEPC (PC-3 and PC-3M) and Taxane Resistance ARLow/mCRPC (DUTXR): PC-3, PC-3M and DUTXR cell lines were treated with CONV-TOPO, METRO-TOPO, CONV-DTX and combination (CONV-DTX+METRO-TOPO) treatment, and cell cytotoxicity and caspase3/7 levels ware assed A) Cytotoxicity profiling by MTT showed combination (CONV-DTX+METRO-TOPO) reduces highest cell survival compared with other treatments for all PCa cell lines, CONV-TOPO>CONV-DTX>METRO-TOPO B) Caspase3/7 activity showed combination (CONV-DTX+METRO-TOPO) treatment reduced apoptosis the greatest compared to other treatments in all cell lines. C) Cytation5 images showed treatment effects on the cell lines PC-3M and DUTXR. Results showed significantly higher cell death in METRO compared to CONV treatment for both the cell lines and combination (CONV-DTX+METRO-TOPO) treatment reduced cell death greater for both PCa cell lines. ImageJ analysis showed significant differences in cell density for CONV-TOPO, CONV-DTX, METRO-TOPO, and combination (CONV-DTX+METRO-TOPO) treatment in PC-3, PC-3M, and DUTXR cell lines. (*p ≤ 0.05). [file crc-22-0427-s08.pptx]

## Slide 1
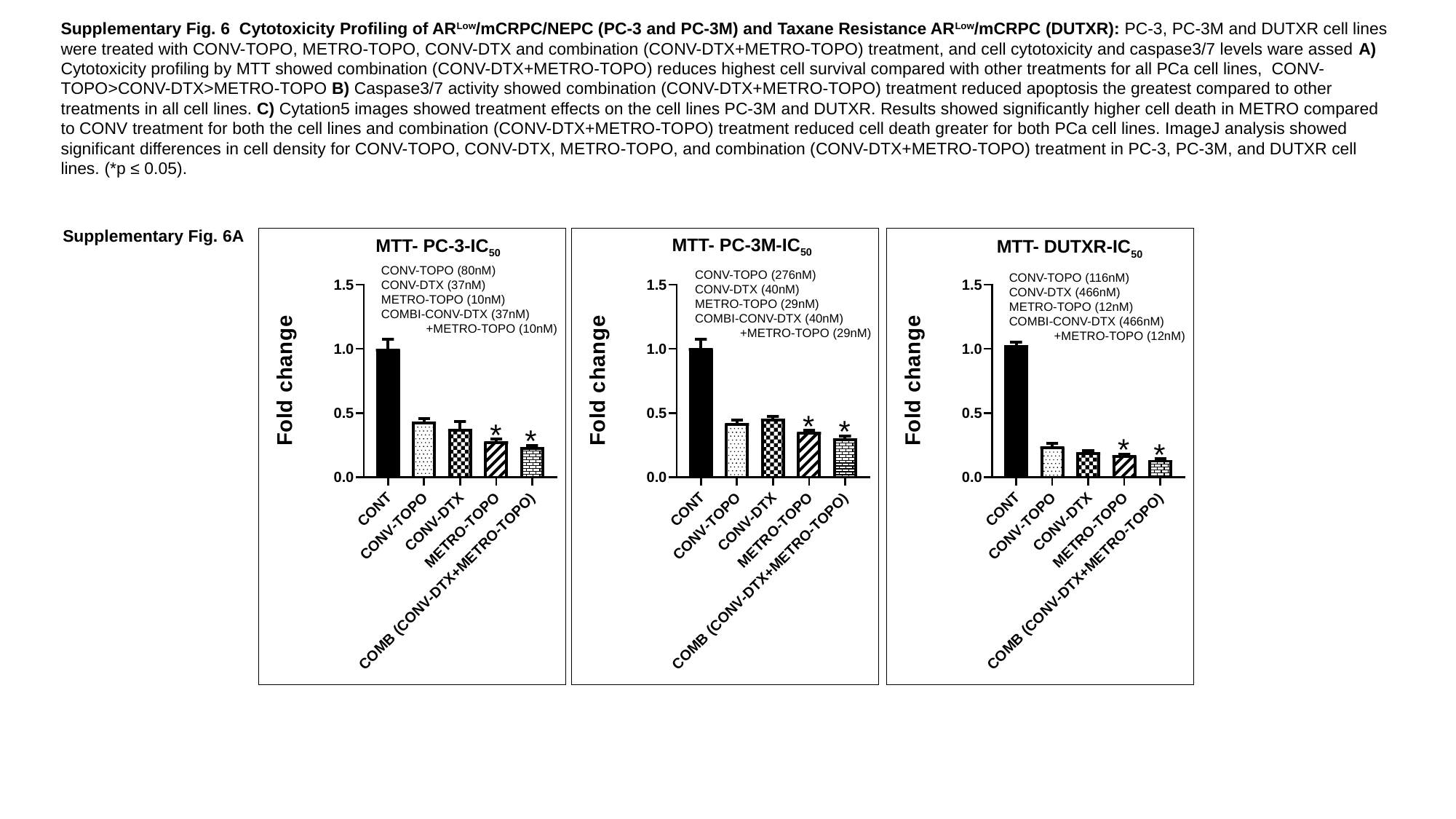

Supplementary Fig. 6 Cytotoxicity Profiling of ARLow/mCRPC/NEPC (PC-3 and PC-3M) and Taxane Resistance ARLow/mCRPC (DUTXR): PC-3, PC-3M and DUTXR cell lines were treated with CONV-TOPO, METRO-TOPO, CONV-DTX and combination (CONV-DTX+METRO-TOPO) treatment, and cell cytotoxicity and caspase3/7 levels ware assed A) Cytotoxicity profiling by MTT showed combination (CONV-DTX+METRO-TOPO) reduces highest cell survival compared with other treatments for all PCa cell lines, CONV-TOPO>CONV-DTX>METRO-TOPO B) Caspase3/7 activity showed combination (CONV-DTX+METRO-TOPO) treatment reduced apoptosis the greatest compared to other treatments in all cell lines. C) Cytation5 images showed treatment effects on the cell lines PC-3M and DUTXR. Results showed significantly higher cell death in METRO compared to CONV treatment for both the cell lines and combination (CONV-DTX+METRO-TOPO) treatment reduced cell death greater for both PCa cell lines. ImageJ analysis showed significant differences in cell density for CONV-TOPO, CONV-DTX, METRO-TOPO, and combination (CONV-DTX+METRO-TOPO) treatment in PC-3, PC-3M, and DUTXR cell lines. (*p ≤ 0.05).
Supplementary Fig. 6A
MTT- PC-3M-IC50
MTT- PC-3-IC50
MTT- DUTXR-IC50
CONV-TOPO (80nM)
CONV-DTX (37nM)
METRO-TOPO (10nM)
COMBI-CONV-DTX (37nM)
 +METRO-TOPO (10nM)
CONV-TOPO (276nM)
CONV-DTX (40nM)
METRO-TOPO (29nM)
COMBI-CONV-DTX (40nM)
 +METRO-TOPO (29nM)
CONV-TOPO (116nM)
CONV-DTX (466nM)
METRO-TOPO (12nM)
COMBI-CONV-DTX (466nM)
 +METRO-TOPO (12nM)
*
*
*
*
*
*

## Slide 2
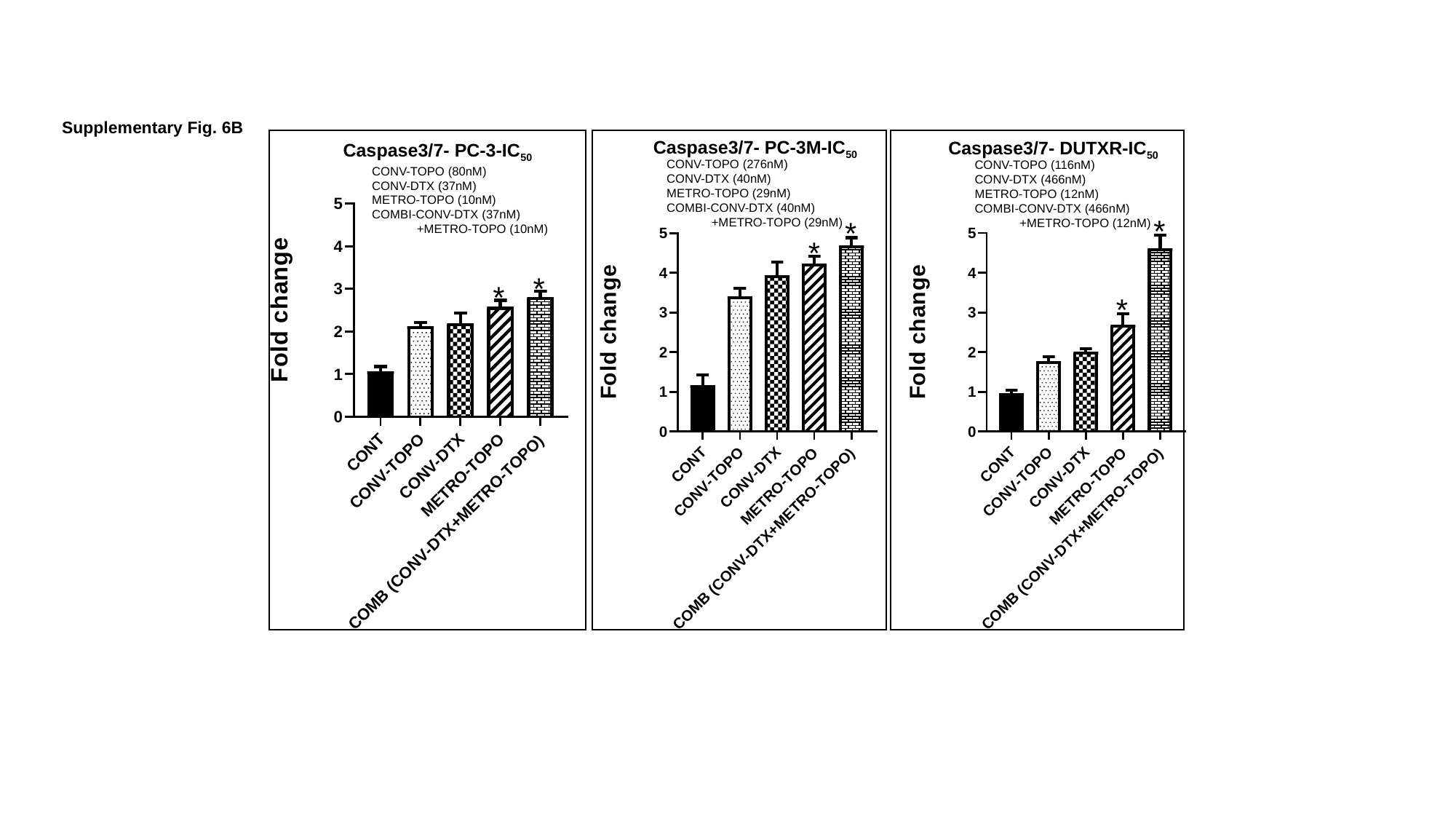

Supplementary Fig. 6B
Caspase3/7- PC-3M-IC50
Caspase3/7- DUTXR-IC50
Caspase3/7- PC-3-IC50
CONV-TOPO (276nM)
CONV-DTX (40nM)
METRO-TOPO (29nM)
COMBI-CONV-DTX (40nM)
 +METRO-TOPO (29nM)
CONV-TOPO (116nM)
CONV-DTX (466nM)
METRO-TOPO (12nM)
COMBI-CONV-DTX (466nM)
 +METRO-TOPO (12nM)
CONV-TOPO (80nM)
CONV-DTX (37nM)
METRO-TOPO (10nM)
COMBI-CONV-DTX (37nM)
 +METRO-TOPO (10nM)
*
*
*
*
*
*

## Slide 3
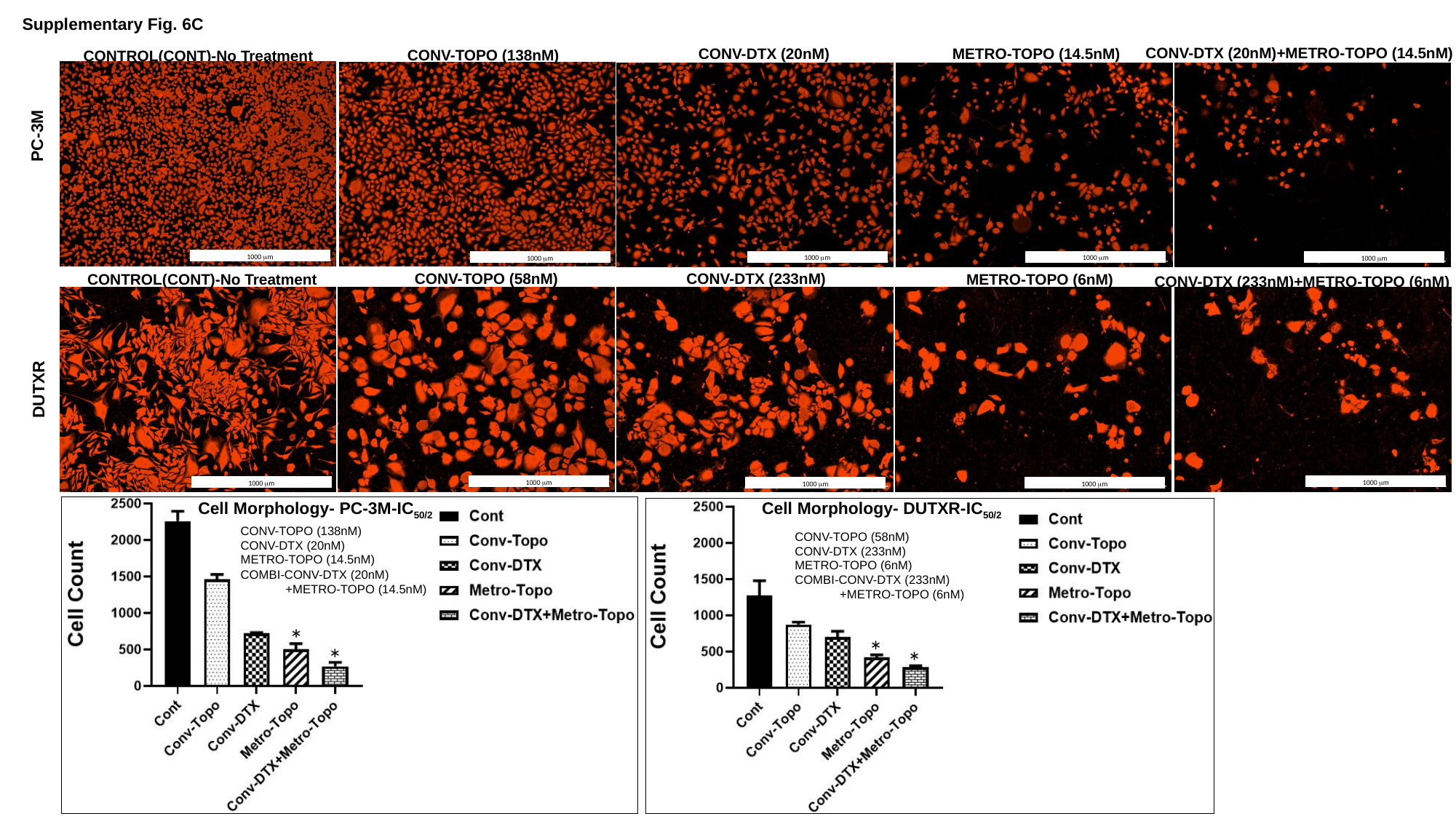

Supplementary Fig. 6C
CONV-DTX (20nM)+METRO-TOPO (14.5nM)
METRO-TOPO (14.5nM)
CONV-DTX (20nM)
CONV-TOPO (138nM)
CONTROL(CONT)-No Treatment
PC-3M
CONV-TOPO (58nM)
CONV-DTX (233nM)
CONTROL(CONT)-No Treatment
METRO-TOPO (6nM)
CONV-DTX (233nM)+METRO-TOPO (6nM)
DUTXR
Cell Morphology- DUTXR-IC50/2
Cell Morphology- PC-3M-IC50/2
1000 mm
1000 mm
1000 mm
1000 mm
1000 mm
1000 mm
1000 mm
1000 mm
1000 mm
1000 mm
CONV-TOPO (138nM)
CONV-DTX (20nM)
METRO-TOPO (14.5nM)
COMBI-CONV-DTX (20nM)
 +METRO-TOPO (14.5nM)
CONV-TOPO (58nM)
CONV-DTX (233nM)
METRO-TOPO (6nM)
COMBI-CONV-DTX (233nM)
 +METRO-TOPO (6nM)
*
*
*
*
