# Supplementary material for: Metronomic Administration of Topotecan Alone and in Combination with Docetaxel Inhibits Epithelial–mesenchymal Transition in Aggressive Variant Prostate Cancers
Source: Cancer Res Commun. 2023 Jul 19;3(7):1286–311. doi: 10.1158/2767-9764.CRC-22-0427 (PMC10355222; doi:10.1158/2767-9764.CRC-22-0427)
Supplement: Supplementary Figure 7 — Supplementary Fig. 7 shows Single-Cell transcriptomics; Identifies epithelial to mesenchymal transition (EMT) marker TGFB1 in ARLow/mCRPC/NEPC (PC-3 spheroid CONT-No treatment vs, extended exposure-EE and conventional exposure-CONV). Result showed TGFB1 expression decreases after EE compared to CONV treatment, which indicated EE moderated stemness in ARLow/mCRPC/NEPC. Single-cell RNA sequencing used the droplet sequencing method (10X Genomics). Each dot represents a single cell. t-distributed stochastic neighbor embedding (tSNE) plots showing the comparison between the single-cell clusters as shown. [file crc-22-0427-s09.pptx]

## Slide 1
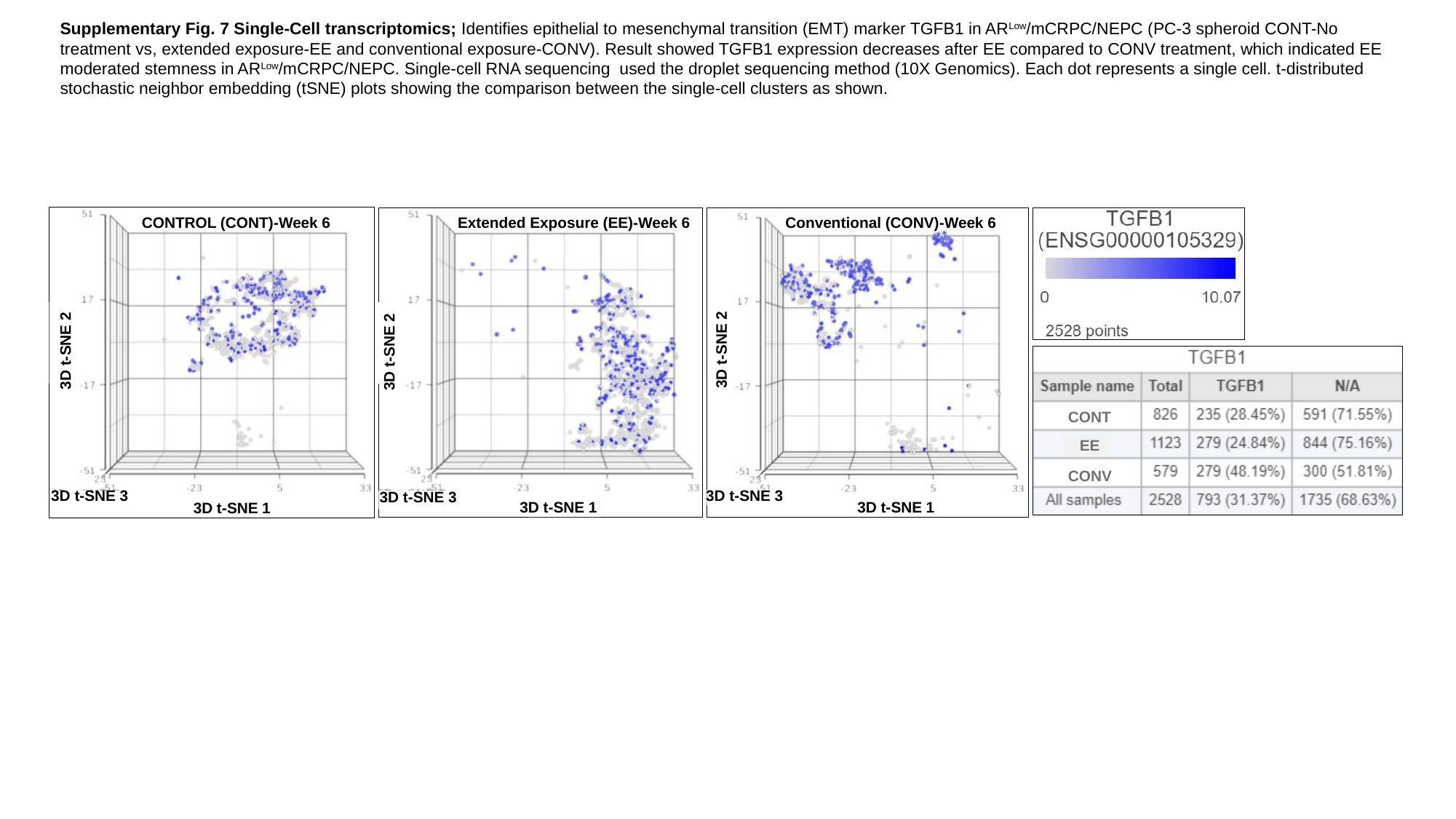

Supplementary Fig. 7 Single-Cell transcriptomics; Identifies epithelial to mesenchymal transition (EMT) marker TGFB1 in ARLow/mCRPC/NEPC (PC-3 spheroid CONT-No treatment vs, extended exposure-EE and conventional exposure-CONV). Result showed TGFB1 expression decreases after EE compared to CONV treatment, which indicated EE moderated stemness in ARLow/mCRPC/NEPC. Single-cell RNA sequencing used the droplet sequencing method (10X Genomics). Each dot represents a single cell. t-distributed stochastic neighbor embedding (tSNE) plots showing the comparison between the single-cell clusters as shown.
CONTROL (CONT)-Week 6
Extended Exposure (EE)-Week 6
Conventional (CONV)-Week 6
CONT
EE
CONV
3D t-SNE 2
3D t-SNE 2
3D t-SNE 2
3D t-SNE 3
3D t-SNE 3
3D t-SNE 3
3D t-SNE 1
3D t-SNE 1
3D t-SNE 1
