# Supplementary material for: Metronomic Administration of Topotecan Alone and in Combination with Docetaxel Inhibits Epithelial–mesenchymal Transition in Aggressive Variant Prostate Cancers
Source: Cancer Res Commun. 2023 Jul 19;3(7):1286–311. doi: 10.1158/2767-9764.CRC-22-0427 (PMC10355222; doi:10.1158/2767-9764.CRC-22-0427)
Supplement: Supplementary Figure 8 — Supplementary Fig. 8 shows Flow Cytometry; Prostate cancer cell lines stained with stemness markers (CD44, CD133 and CD44/133) to determine the percent of CD44+, CD133+ and double positive cells among PCa subtypes. Treatments with CONV-TOPO, METRO-TOPO and CONV-DTX+METRO-TOPO reduced stemness. METRO-TOPO reduced CD44+ cells to a greater extent compared to CONV-TOPO and CONV-DTX treatments, whereas combination treatment with CONV-DTX+METRO-TOPO reduced stemness (CD44+ cells) the most in ARHigh/mCRPC/NEPC. A) PC-3M, B) DU145 cell lines. [file crc-22-0427-s10.pptx]

## Slide 1
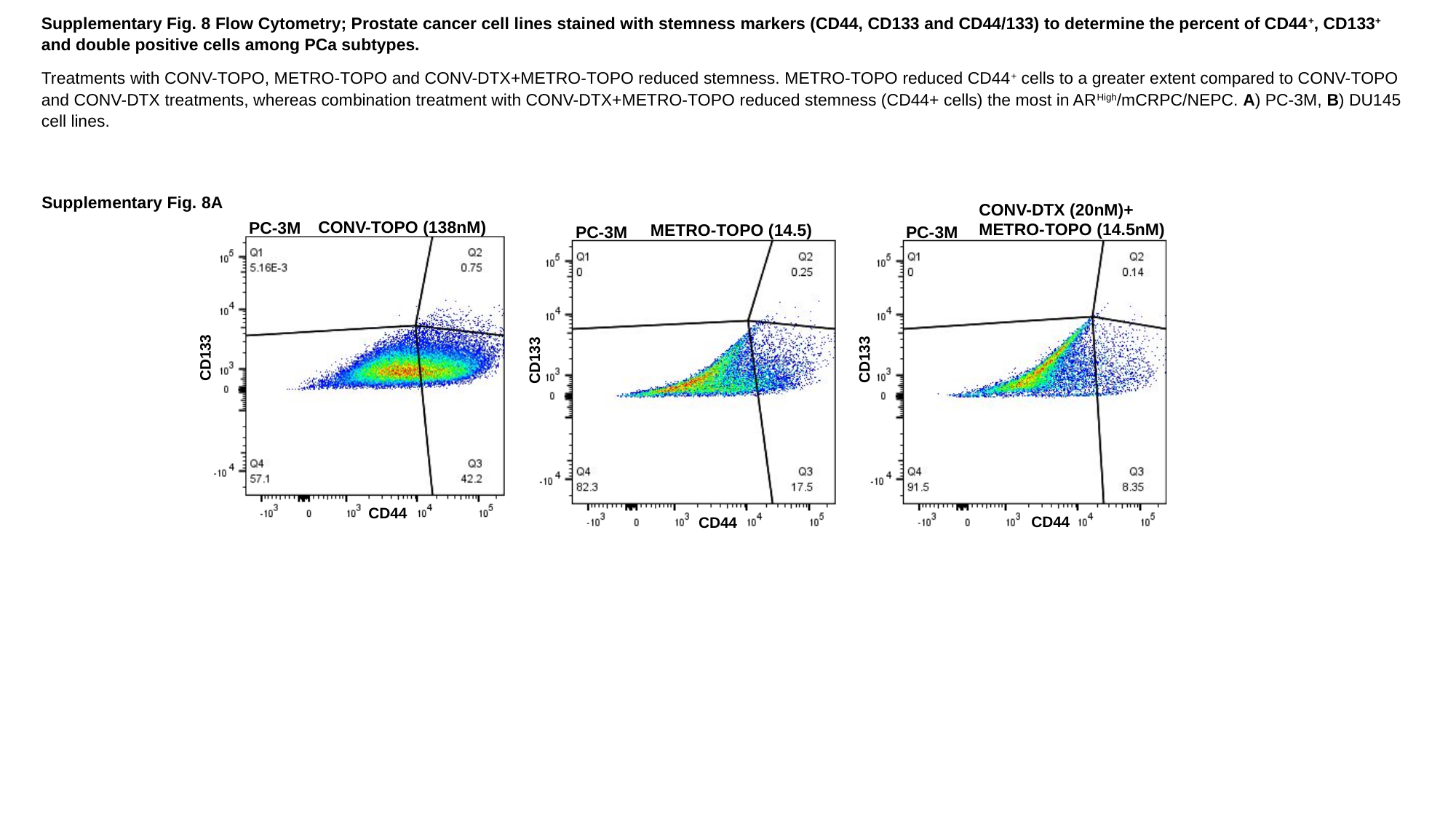

Supplementary Fig. 8 Flow Cytometry; Prostate cancer cell lines stained with stemness markers (CD44, CD133 and CD44/133) to determine the percent of CD44+, CD133+ and double positive cells among PCa subtypes.
Treatments with CONV-TOPO, METRO-TOPO and CONV-DTX+METRO-TOPO reduced stemness. METRO-TOPO reduced CD44+ cells to a greater extent compared to CONV-TOPO and CONV-DTX treatments, whereas combination treatment with CONV-DTX+METRO-TOPO reduced stemness (CD44+ cells) the most in ARHigh/mCRPC/NEPC. A) PC-3M, B) DU145 cell lines.
Supplementary Fig. 8A
CONV-DTX (20nM)+
METRO-TOPO (14.5nM)
CONV-TOPO (138nM)
PC-3M
METRO-TOPO (14.5)
PC-3M
PC-3M
CD133
CD133
CD133
CD44
CD44
CD44

## Slide 2
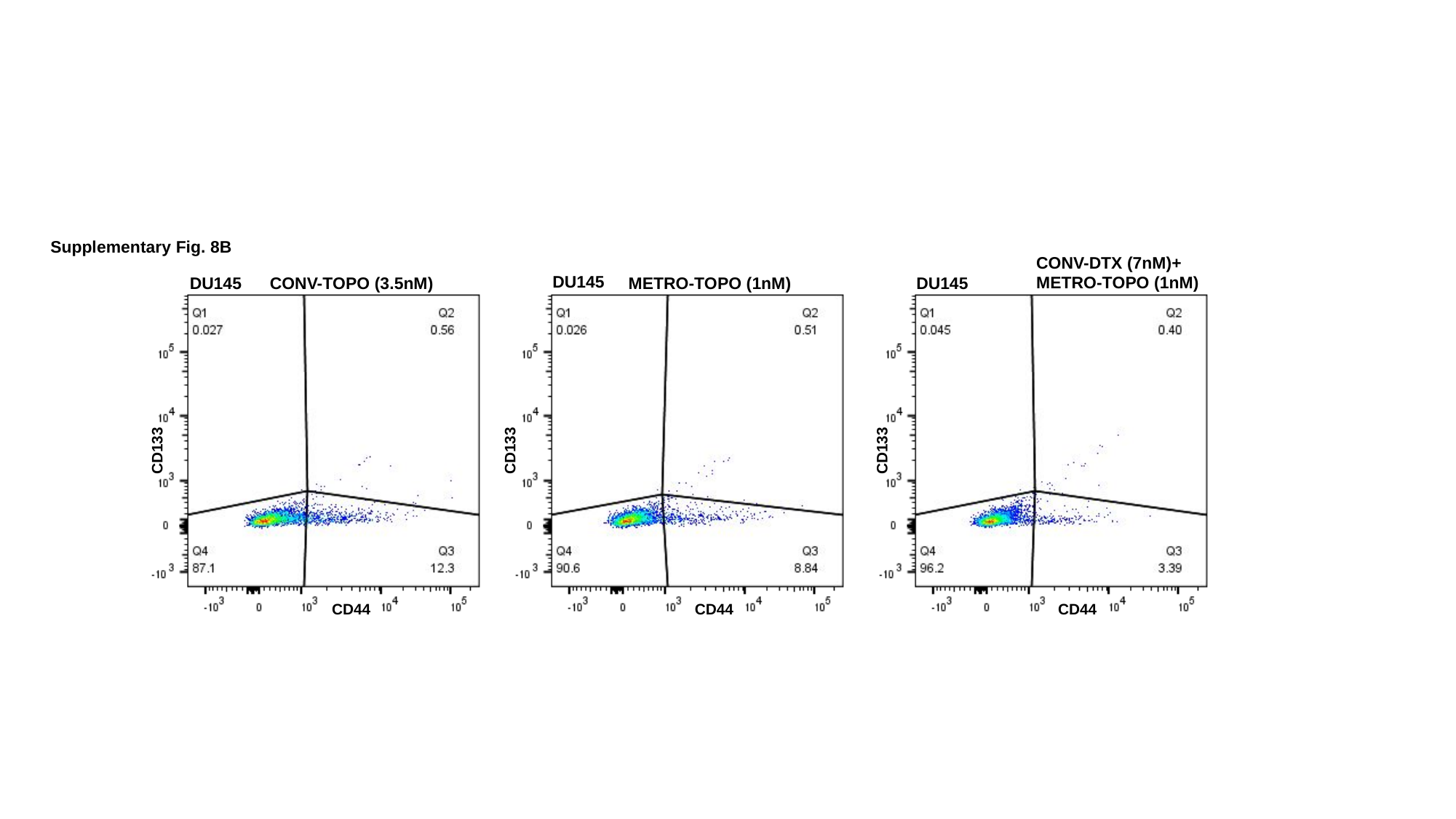

Supplementary Fig. 8B
CONV-DTX (7nM)+
METRO-TOPO (1nM)
DU145
METRO-TOPO (1nM)
CONV-TOPO (3.5nM)
DU145
DU145
CD44
CD44
CD44
CD133
CD133
CD133
