# Supplementary material for: Metronomic Administration of Topotecan Alone and in Combination with Docetaxel Inhibits Epithelial–mesenchymal Transition in Aggressive Variant Prostate Cancers
Source: Cancer Res Commun. 2023 Jul 19;3(7):1286–311. doi: 10.1158/2767-9764.CRC-22-0427 (PMC10355222; doi:10.1158/2767-9764.CRC-22-0427)
Supplement: Supplementary Figure 9 — Supplementary Fig. 9 shows Fluorescent-Activated Cell Sorting CD44High: PC-3M cell line stained with stemness markers (CD44) and sorted CD44+ cells, followed by CONV-TOPO, METRO-TOPO, CONV-DTX, and combination (CONV-DTX+METRO-TOPO) therapy and cell cytotoxicity and caspase3/7 levels assessed A) Cytotoxicity profiling by MTT showed combination (CONV-DTX+METRO-TOPO) reduces cell survival compared with other treatments CONV-TOPO>CONV-DTX>METRO-TOPO B) AR-mCRPC- PC-3M showed combination (CONV-DTX+METRO-TOPO) reduces levels the most of apoptosis compared to other treatments (*p<0.05). [file crc-22-0427-s11.pptx]

## Slide 1
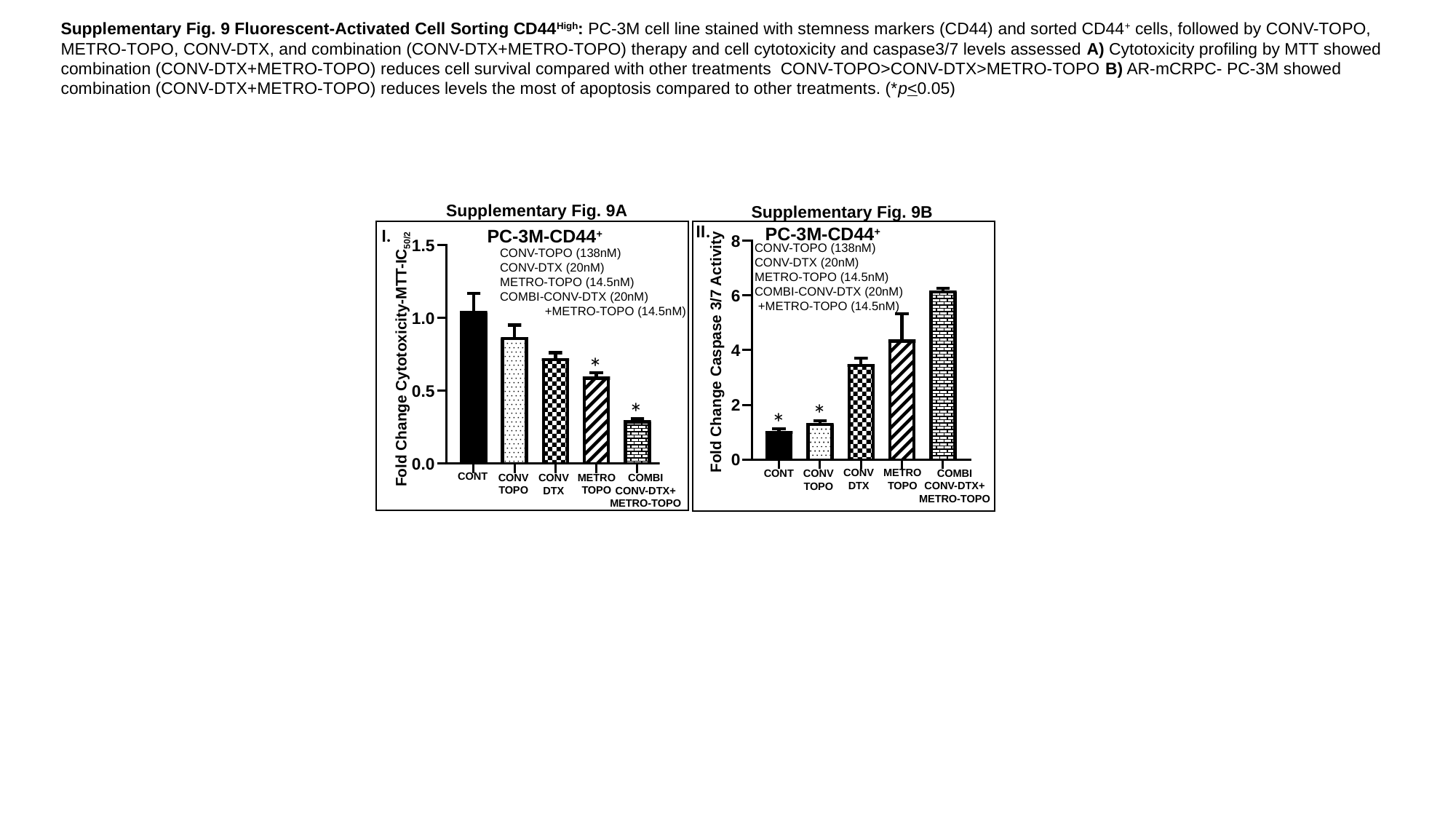

Supplementary Fig. 9 Fluorescent-Activated Cell Sorting CD44High: PC-3M cell line stained with stemness markers (CD44) and sorted CD44+ cells, followed by CONV-TOPO, METRO-TOPO, CONV-DTX, and combination (CONV-DTX+METRO-TOPO) therapy and cell cytotoxicity and caspase3/7 levels assessed A) Cytotoxicity profiling by MTT showed combination (CONV-DTX+METRO-TOPO) reduces cell survival compared with other treatments CONV-TOPO>CONV-DTX>METRO-TOPO B) AR-mCRPC- PC-3M showed combination (CONV-DTX+METRO-TOPO) reduces levels the most of apoptosis compared to other treatments. (*p<0.05)
Supplementary Fig. 9A
Supplementary Fig. 9B
PC-3M-CD44+
Fold Change Cytotoxicity-MTT-IC50/2
CONT
CONV
TOPO
METRO
TOPO
CONV
DTX
COMBI
CONV-DTX+
METRO-TOPO
II.
PC-3M-CD44+
CONV
DTX
METRO
TOPO
COMBI
CONV-DTX+
METRO-TOPO
CONT
CONV
TOPO
Fold Change Caspase 3/7 Activity
I.
CONV-TOPO (138nM)
CONV-DTX (20nM)
METRO-TOPO (14.5nM)
COMBI-CONV-DTX (20nM)
 +METRO-TOPO (14.5nM)
CONV-TOPO (138nM)
CONV-DTX (20nM)
METRO-TOPO (14.5nM)
COMBI-CONV-DTX (20nM)
 +METRO-TOPO (14.5nM)
*
*
*
*
